# Heteromeric clusters of ubiquitinated ER-shaping proteins drive ER-phagy

**DOI:** 10.1038/s41586-023-06090-9

**Published:** 2023-05-24

**Authors:** Hector Foronda, Yangxue Fu, Adriana Covarrubias-Pinto, Hartmut T. Bocker, Alexis González, Eric Seemann, Patricia Franzka, Andrea Bock, Ramachandra M. Bhaskara, Lutz Liebmann, Marina E. Hoffmann, Istvan Katona, Nicole Koch, Joachim Weis, Ingo Kurth, Joseph G. Gleeson, Fulvio Reggiori, Gerhard Hummer, Michael M. Kessels, Britta Qualmann, Muriel Mari, Ivan Dikić, Christian A. Hübner

**Affiliations:** 1 Institute of Human Genetics, Jena University Hospital, Friedrich Schiller University, Jena, Germany; 2 Institute of Biochemistry II, Goethe University School of Medicine, Frankfurt am Main, Germany; 3 Institute for Biochemistry I, Jena University Hospital, Friedrich Schiller University, Jena, Germany; 4 Buchmann Institute for Molecular Life Sciences, Goethe University Frankfurt, Frankfurt am Main, Germany; 5 Department of Theoretical Biophysics, Max Planck Institute of Biophysics, Frankfurt am Main, Germany; 6 Institute of Neuropathology, RWTH Aachen University Hospital, Aachen, Germany; 7 Department of Neurosciences, Rady Children’s Institute for Genomic Medicine Howard Hughes Medical Institute, University of California, San Diego, La Jolla, CA USA; 8 Department of Biomedical Sciences of Cells and Systems, University of Groningen, University Medical Center Groningen, Groningen, The Netherlands; 9 Department of Biomedicine, Aarhus University, Aarhus C, Denmark; 10 Aarhus Institute of Advanced Studies (AIAS), Aarhus University, Aarhus C, Denmark; 11 Institute of Biophysics, Goethe University Frankfurt, Frankfurt am Main, Germany; 12 Center for Rare Diseases, Jena University Hospital, Friedrich Schiller University, Jena, Germany; 13 Present Address: Blink AG, Jena, Germany; 14 Present Address: Institute for Human Genetics and Genomic Medicine, Medical Faculty, RWTH Aachen University, Aachen, Germany

**Keywords:** Autophagy, Neurodegeneration, Ubiquitylation, Endoplasmic reticulum

## Abstract

Membrane-shaping proteins characterized by reticulon homology domains play an important part in the dynamic remodelling of the endoplasmic reticulum (ER). An example of such a protein is FAM134B, which can bind LC3 proteins and mediate the degradation of ER sheets through selective autophagy (ER-phagy)^1^. Mutations in *FAM134B* result in a neurodegenerative disorder in humans that mainly affects sensory and autonomic neurons^2^. Here we report that ARL6IP1, another ER-shaping protein that contains a reticulon homology domain and is associated with sensory loss^3^, interacts with FAM134B and participates in the formation of heteromeric multi-protein clusters required for ER-phagy. Moreover, ubiquitination of ARL6IP1 promotes this process. Accordingly, disruption of *Arl6ip1* in mice causes an expansion of ER sheets in sensory neurons that degenerate over time. Primary cells obtained from *Arl6ip1*-deficient mice or from patients display incomplete budding of ER membranes and severe impairment of ER-phagy flux. Therefore, we propose that the clustering of ubiquitinated ER-shaping proteins facilitates the dynamic remodelling of the ER during ER-phagy and is important for neuronal maintenance.

## Abstract (web summary)

The membrane-shaping protein ARL6IP1 is involved in the selective degradation of the endoplasmic reticulum, and this process depends on its ubiquitination and interaction with other membrane-shaping proteins such as FAM134B.

## Main

In a previous study^2^, we identified *FAM134B* loss-of-function mutations in patients with autosomal recessive hereditary sensory and autonomic neuropathy (HSAN)^2^. This disorder is characterized by the degeneration of sensory and autonomic neurons that leads to numbness and the inability to feel pain. These symptoms in turn cause severe injuries and tissue damage^2^. Our studies further showed that FAM134B is an ER-resident membrane-shaping protein that can bind LC3 proteins and mediate the engulfment of parts of ER sheets by autophagosomes and their subsequent lysosomal degradation^1^. The neurodegeneration that occurs in humans is also observed in *Fam134b*-deficient mice^1^. Together with the consequences observed following the disruption of the functional counterpart of FAM134B in yeast (Atg40)^4^, these results suggest that the role of ER-phagy in cell viability is evolutionarily conserved.

Within the past decade, several other ER-resident membrane-shaping proteins with central reticulon homology domains (RHDs) have been associated with similar neurodegenerative disorders, including ATL1, ATL3, REEP1, REEP2, SPAST, RTN2, ARL6IP1 and LNPK^5–7^. Mutations in *ARL6IP1* cause SPG61, a neurodegenerative disorder characterized by progressive leg spasticity (hereditary spastic paraplegia (HSP)) in combination with loss of sensory and pain perception, thus overlapping with typical symptoms of HSAN^2,3,8^. The underlying mechanisms, however, remained largely elusive. Here we show that membrane-embedded clusters of ubiquitinated ARL6IP1 and FAM134B are required for effective ER remodelling and ER-phagy, defects of which result in severe neurodegeneration.

## Degeneration of neurons in *Arl6ip1* knockout mice

**Fig. 1:**
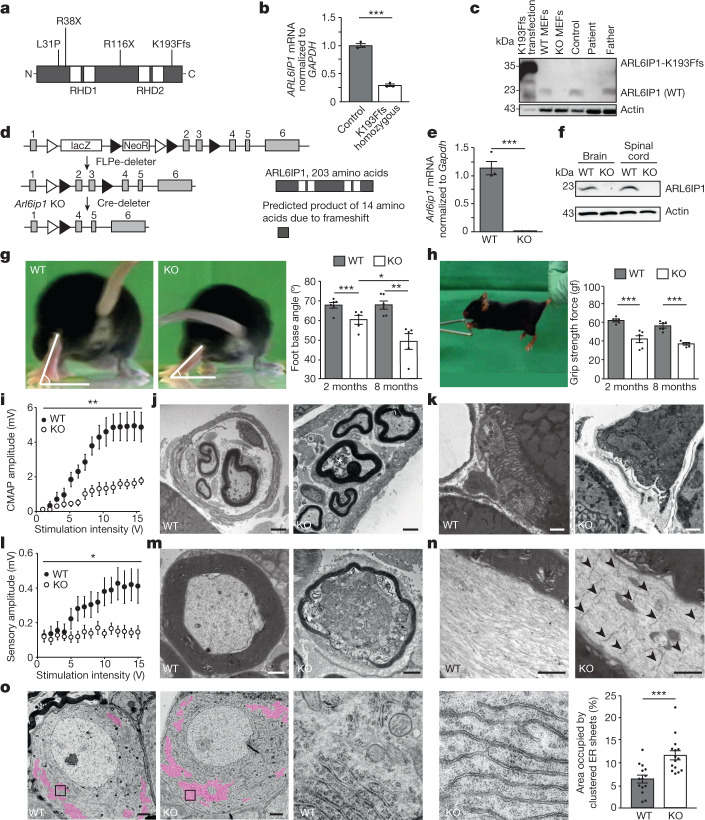
Neurodegeneration with ER sheet expansion in *Arl6ip1* KO mice. **a**, Schematic of disease-associated ARL6IP1 variants. **b**, No *ARL6IP1* transcripts were detected in fibroblasts obtained from a patient carrying a homozygous mutation of *ARL6IP1*^*K193Ffs*^ (qPCR with three replicates, two-sided unpaired Student’s *t-*test, *P* = 0.0001). **c**, No variant protein was detected in cells from the patient (two experiments). **d**, *Arl6ip1* KO strategy. Frt sites, white triangles; *loxP* sites, black triangles. Predicted 14 amino acid product for the KO allele. **e**, No *Arl6ip1* transcripts were detected in KO MEFs (qPCR with three replicates, two-sided unpaired Student’s t-test, *P* = 0.0007). **f**, Absence of ARL6IP1 in KO tissue lysates (two experiments). **g**, Decreasing foot base angle in KO mice (*n* = 6 WT mice and *n* = 5 KO mice; two-sided unpaired Student’s *t*-test, 2 month-old WT versus KO, *P* = 0.0007; 8-month-old WT versus KO, *P* = 0.0016; KO 2 months versus 8 months, *P* = 0.038). **h**, Diminished forelimb grip strength in 2-month-old (*P* = 0.0004) and 8-month-old (*P* = 0.0001) KO mice (*n* = 6 WT and KO mice each; two-sided unpaired Student’s *t*-test). **i**, Decreased compound muscle action potentials (CMAPs) in KO mice (*n* = 6 WT mice and *n* = 7 KO mice; repeated-measures analysis of variance (ANOVA), *F* = 18.6, *P* = 0.0015). **j**, TEM of intact WT and degenerating KO intramuscular nerve fibre (indicated by the asterisk). **k**, TEM of intact WT and degenerating KO motor end plate. **l**, Decreased sensory amplitudes in KO mice (*n* = 6 WT mice and *n* = 7 KO mice; repeated-measures ANOVA, *F* = 6.08, *P* = 0.0314). **m**, TEM of transversely cut intact WT and degenerating KO sciatic nerve axon. **n**, TEM of longitudinally cut sciatic nerve axons with ladder-like transverse ER sheet expansions (arrowheads) in KO but not WT mice. For **i**–**n**, analyses were performed using 6-month-old mice. **o**, TEM images (left) and quantification (right) show ER sheet expansions in lumbar spinal ganglion neurons in 12-month-old KO mice. ER sheet areas are coloured and higher magnifications are indicated. *n* = 100 cells from *n* = 3 WT mice and *n* = 177 cells from *n* = 3 KO mice; two-sided unpaired Student’s *t*-test, *P* = 0.0006. Data shown as the mean ± s.e.m. Scale bars, 1 µm (**j**,**m**,**n**), 2 µm (**k**) or 2.5 µm (**o**). Source Data

**Extended Data Fig. 1:**
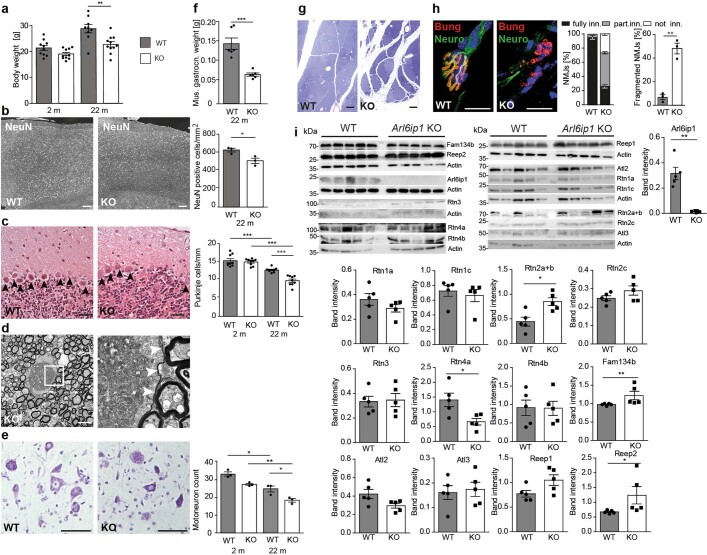
Phenotype of *Arl6ip1* KO mice. **a)** The body weight of KO mice is reduced (2 months: 15 WT and 15 KO mice; 22 months: 9 WT and 11 KO mice; 2-sided unpaired Student’s t-test: p = 0.0034). **b)** Cortical neurons are decreased in aged KO mice. Cross sections of the motor cortex of 22-month-old WT and KO mice with NeuN-labelled neurons (1 exp.; 3 mice per genotype; 2-sided unpaired Student’s t-test: p = 0.0312). Scale bars: 50 µm. **c)** Progressive Purkinje cell loss in KO mice. Representative images of the Purkinje cell layer of HE-stained sagittal cerebellum sections of 22-month-old WT and KO mice (1 exp.; n = 3 mice with 3 sections each per genotype; 2-sided unpaired Student’s t-test: WT 2 versus WT 22 months, p = 0.0002; KO 2 versus KO 22 months p = 0.0001, KO versus WT 22 months p = 0.0001). Scale bars: 100 µm. **d)** Swollen corticospinal axon in the lumbar spinal cord of a 6-month-old KO mouse (1 exp.). TEM of a horizontal spinal cord section. Scale bar: 5 µm. The higher magnification shows the accumulation of granular electron-dense material, defective organelles and tubulofilamentous material. The axon is surrounded by a distended myelin sheath (arrowheads). Scale bar: 1 µm. **e)** Progressive α-motoneuron loss in the thoracic spinal cord of KO mice. Nissl-stained horizontal sections (1 exp.; 3 mice per genotype; 2-sided unpaired Student’s t-test: WT 22 versus KO 22 months p = 0.0322, KO 2 versus KO 22 months p = 0.0018, WT 2 versus WT 22 months p = 0.0154). **f)** The Musculus gastrocnemius mass is reduced in 2-month-old KO mice (1 exp.; 3 WT and 3 KO mice with 2 samples each (left and right); 2-sided unpaired Student’s t-test: p = 0.001). **g)** Grouped atrophic skeletal muscle fibres in 20-week-old KO mice. Toluidine-blue stained semi-thin sections (1 exp.). Scale bar: 100 µm. **h)** Fragmentation and innervation loss of neuromuscular junctions (NMJ) in the Musculus gastrocnemius of 2-month-old KO mice stained with α-bungarotoxin (red), neurofilament 200 (green) and Hoechst 33258 (1 exp.; 3 mice per genotype). 2-sided unpaired Student’s t-test for fragmentation (p = 0.0012). **i)** The abundance of some ER proteins with RHDs is changed in brain lysates of 5-month-old KO mice (1 exp. with 5 samples per genotype; 2-sided Mann-Whitney-U-test: Rtn2a+b p = 0.0317, Rtn4a p = 0.0317, Fam134b p = 0.0079, Reep2 p = 0.0159). Quantitative data are shown as mean ± SEM. Source Data

To resolve the pathophysiology of the ARL6IP1-related disorder, we studied fibroblasts obtained from a patient with SPG61. This patient harboured the homozygous carboxy-terminal frameshift mutation *ARL6IP1* c.577–580delAAAC (NCBI Nucleotide database identifier NM_015161.3; K193Ffs variant) (Fig. 1a). Fibroblasts obtained from the patient’s father, who was unaffected and a heterozygous carrier, and from an unrelated healthy individual (as a control) were also analysed. In silico analysis indicated that this frameshift mutation is predicted to result in the replacement of the 11 amino acids of the C terminus by 36 alternative residues^3^. Suggesting nonsense-mediated decay in vivo, *ARL6IP1* transcripts were absent in the patient’s cells, as assessed by real-time PCR with exon spanning primers annealing 5′ end of the deletion (Fig. 1b). Immunoblot analyses of lysates from cells transfected with a plasmid encoding the K193Ffs variant produced a band with a slightly higher molecular weight (Fig. 1c). No variant protein was detected in fibroblasts obtained from the patient’s father or from the patient when using an antibody directed against the cytoplasmic loop of ARL6IP, a result that is in agreement with nonsense-mediated decay (Fig. 1c). Because the *ARL6IP1* c.577–580delAAAC variant represents a knockout (KO) allele, we generated *Arl6ip1* KO mice to model the ARL6IP1-associated disorder (Fig. 1d). KO of the gene was confirmed by both quantitative PCR (qPCR) of RNA isolated from mouse embryonic fibroblasts (MEFs) (Fig. 1e) and immunoblot analyses of MEFs and tissue lysates (Fig. 1c,f). Compared with wild-type (WT) mice, KO animals did not gain appropriate body weight (Extended Data Fig. 1a). Consistent with the CNS-related phenotypes reported for some patients^8,9^, the weight of the brain in *Arl6ip1* KO mice was significantly decreased (0.46 g in WT mice compared with 0.39 g in KO mice at 6 months of age; *n* = 3, unpaired Student’s *t*-test, *P* = 0.016). Moreover, cortical neuron (Extended Data Fig. 1b) and Purkinje cell (Extended Data Fig. 1c) counts were reduced. As a correlate of the HSP-related gait disorder, we measured the foot base angle of the hind paw at the moment when the toe was lifted^10^ and found that it decreased with age in KO mice (Fig. 1g). Moreover, the grip strength of the upper limbs was reduced in KO mice (Fig. 1h). As reported for deceased patients with HSP, we observed that some axons connecting cortical and spinal cord motor neurons were swollen and full of dysfunctional organelles (Extended Data Fig. 1d). Because some patients develop muscle hypotonia and weakness, we also quantified spinal cord motor neurons, which progressively decreased in KO mice (Extended Data Fig. 1e). In agreement, the electrophysiological analysis showed a severe reduction in compound muscle action potential amplitudes (Fig. 1i). Consistent with neurogenic muscle atrophy, we found reduced musculus gastrocnemius mass (Extended Data Fig. 1f), degenerating grouped skeletal muscle fibres (Extended Data Fig. 1g) as well as degenerating intramuscular nerve fibres (Fig. 1j) and motor end plates (Fig. 1k and Extended Data Fig. 1h) in KO animals. Immunoblot analyses of brain protein lysates showed that the abundance of some ER-resident proteins with RHDs was altered in KO mice (Extended Data Fig. 1i). Sensory fibres were also degenerated, as evidenced by the substantial loss of sensory amplitudes in electrophysiological analyses of peripheral nerves (Fig. 1l). This result correlates with sensory loss and loss of pain perception in patients^3,8^. The ultrastructural analysis of peripheral nerves also showed swollen axons full of dysfunctional organelles and tubulofilamentous material (Fig. 1m), and ladder-like expansions of transverse ER sheet structures (Fig. 1n). This result is similar to that reported previously for mice mutant for both ALT1 and REEP1 (ref. ^11^). An analysis of cell bodies of peripheral sensory neurons (dorsal root ganglia (DRG)) uncovered a substantial expansion of ER sheets in *Arl6ip1* KO mice (Fig. 1o). Because of these observations and similar phenotypes in patients, we propose that ARL6IP1 plays a part in FAM134B-dependent ER-phagy.

## ARL6IP1 is part of ER-phagy complexes

**Fig. 2:**
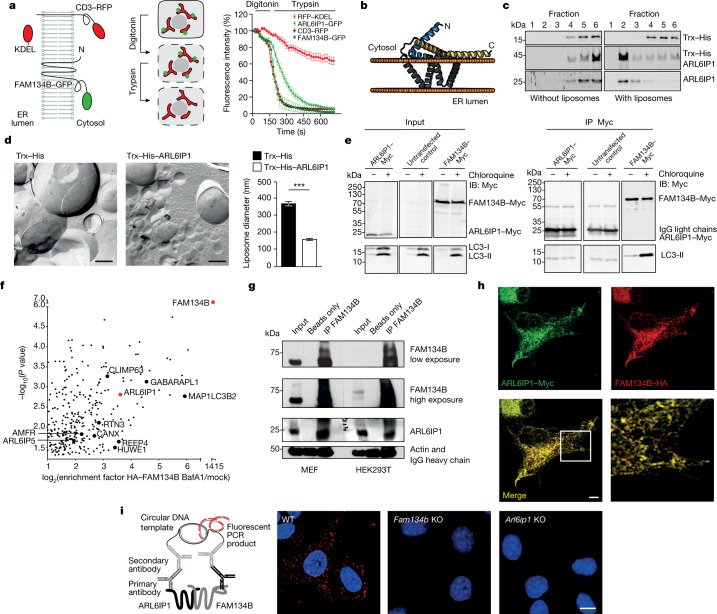
FAM134B interacts with ARL6IP1. **a**, Left, cytosolic location of the ARL6IP1 C terminus. Middle, COS-7 cells transiently expressing RFP-tagged or GFP-tagged ER proteins subjected to fluorescence protease protection assays by sequential administration of digitonin to permeabilize plasma but not intracellular membranes and then trypsin. Right, C-terminal fluorescent tags of ARL6IP1, FAM134B or CD3 are destroyed by trypsin, whereas luminal RFP–KDEL is protected (three experiments with *n* = 29 (CD3–RFP), 16 (RFP–KDEL), 15 (FAM134B–GFP) and 22 (ARL6IP1–GFP) cells). **b**, Three-dimensional model of ARL6IP1 built using AlphaFold2 showing the relative organization of key structural elements (grey and yellow) and their relative orientation in a model bilayer (orange beads). **c**, Recombinant Trx–His–ARL6IP1 and untagged ARL6IP1 float with lipid membranes in sucrose density gradients to the liposome fraction 2 (immunoblot analysis, *n* = 2). **d**, Left, TEM images of freeze-fractured incubations of liposomes with Trx–His or recombinant Trx–His–ARL6IP1. Right, mean liposome diameters are decreased with Trx–His–ARL6IP1 (1 experiment with *n* = 1,817 (Trx–His; average 380 nm) and 1,824 (Trx–His–ARL6IP1; average 150 nm) vesicles analysed; two-sided Mann–Whitney *U*-test, *P* = 0.0001). **e**, HEK293T cells were transfected with the indicated constructs and immunoblotted (IB). Pull-down with anti-Myc coupled beads shows that LC3-II co-precipitates with FAM134B–Myc but not ARL6IP1–Myc (*n* = 1). **f**, The FAM134B interactome in U2OS cells includes ARL6IP1 (single-sided volcano plot). Notable hits with a log_2_(enrichment factor) > 1 and –log_10_(*P* value) > 1.3 are highlighted (one-sided paired Student’s *t*-test with three biological replicates). **g**, Co-precipitation of endogenous ARL6IP1 and FAM134B from MEFs and from HEK293T cells (*n* = 1). IP, immunoprecipitation. **h**, Overexpressed ARL6IP1–Myc and FAM134B–HA co-localize in MEFs. **i**, Proximity ligation assays suggest a proximity of less than 40 nm between endogenous ARL6IP1 and FAM134B. Specificity was confirmed by the absence of signals in the respective KO MEFs. Quantitative data are shown as the mean ± s.e.m. Scale bars, 200 nm (**d**) or 5 µm (**h**,**i**). Source Data

**Extended Data Fig. 2:**
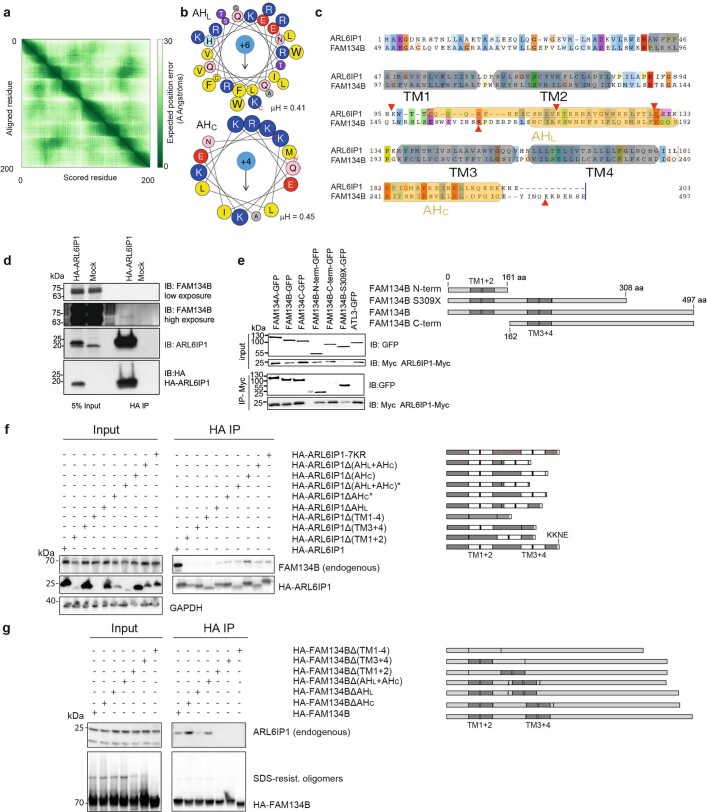
ARL6IP1 and FAM134B share structural features and interact. **a)** Predicted alignment error of the relative organization of key structural elements of ARL6IP1 shown in Fig. 2b. **b)** Helical wheel representation of two cytosolic helical segments with characteristics of amphipathic helices with a large hydrophobic moment and net positive charge (blue circle)^52^ . **c)** Pairwise sequence alignment of ARL6IP1 and FAM134B indicates preserved RHDs, i.e. two helical hairpins TM1+2 and TM3+4 (grey), and two amphipathic helices, AH_L_ and AH_C_ (yellow). Both proteins harbour several predicted ubiquitination sites (red triangles). **d)** Doxycycline induced HA-ARL6IP1 interacts with endogenous FAM134B in U2OS cells (1 exp.). **e)** ARL6IP1 co-precipitates with all known members of the FAM134 family of proteins (1 exp.). The interaction with FAM134B requires the N-terminal part of FAM134B with its first RHD and is independent of its C-terminal coiled-coil domain. Cells were transfected with Myc-ARL6IP1 and the indicated GFP-tagged FAM134B deletion constructs. ATL3, another ER-protein characterised by a RHD, served as a negative control. **f)** The central part of ARL6IP1 with both helical hairpins is involved in the interaction with FAM134B (1 exp.). HEK293T cells were transfected with the indicated HA-tagged ARL6IP1 deletion constructs. WT and variant proteins were pulled down and the endogenous binding partner FAM134B was detected. The deletion sites are indicated as black bars. The replaced lysines for the ARL6IP1-7KR variant are indicated by red bars. Variants marked with * lack the terminal KKNE signal (white bar). **g)** The central part of FAM134B with both helical hairpins is required for the interaction with ARL6IP1 (1exp.). HEK293T cells were transfected with the indicated HA-tagged FAM134B deletion constructs. The deletion sites are indicated as black lines. WT and variant proteins were pulled down and the endogenous binding partner ARL6IP1 detected.

On the basis of previous data^12^ and results of fluorescence protease protection assays, we concluded that ARL6IP1 is characterized by the presence of RHD-like structural elements. That is, two long hydrophobic regions (transmembrane helical hairpins TM1+2 and TM3+4) separated by an accessible linker segment with both the amino and C termini facing the cytoplasm (Fig. 2a). The predicted structural model (produced using AlphaFold) of ARL6IP1 contains two membrane-embedded helical hairpins (TM1+2 and TM3+4) with two amphipathic helices (Fig. 2b and Extended Data Fig. 2a–c). Purified ARL6IP1 bound to liposomes in vitro (Fig. 2c) and increased the proportion of smaller liposomes (Fig. 2d), similar to FAM134B^1,13,14^. Despite predicted putative LC3-interacting regions (LIRs) in the N terminus or the cytoplasmic loop between RHDs, and in contrast to FAM134B, ARL6IP1 did not bind LC3 proteins (Fig. 2e), which suggested that ARL6IP1 is not an ER-phagy receptor on its own. However, ARL6IP1 was detected as an interaction partner in both a yeast two-hybrid screen for FAM134B-binding proteins^1^ and in a proteomics analysis of FAM134B interactors (Fig. 2f). This result indicates that ARL6IP1 may be indirectly linked to the autophagy machinery through FAM134B. A sequence alignment analysis showed that both proteins are closely related and share all the signature membrane remodelling elements (Extended Data Fig. 2c). The co-precipitation of endogenous FAM134B and ARL6IP1 in MEFs or HEK293T cells (Fig. 2g) or endogenous FAM134B and expressed haemagglutinin (HA)-tagged ARL6IP1 (HA–ARL6IP1) in U2OS cells (Extended Data Fig. 2d) confirmed the interaction between both proteins. In agreement, ARL6IP1–Myc and FAM134B–HA co-localized to the same ER regions in MEFs (Fig. 2h). The co-localization results from MEFs was supported by data from proximity ligation assays using antibodies directed against endogenous FAM134B and ARL6IP1 (Fig. 2i). Using different deletion variants of ARL6IP1 and FAM134B, it became evident that their central parts containing the RHDs are required for the interaction between both proteins (Extended Data Fig. 2e–g). Notably, tagged FAM134A and FAM134C, the two homologues of FAM134B, also co-immunoprecipitated with ARL6IP1 in HEK293T cells (Extended Data Fig. 2e).

**Fig. 3:**
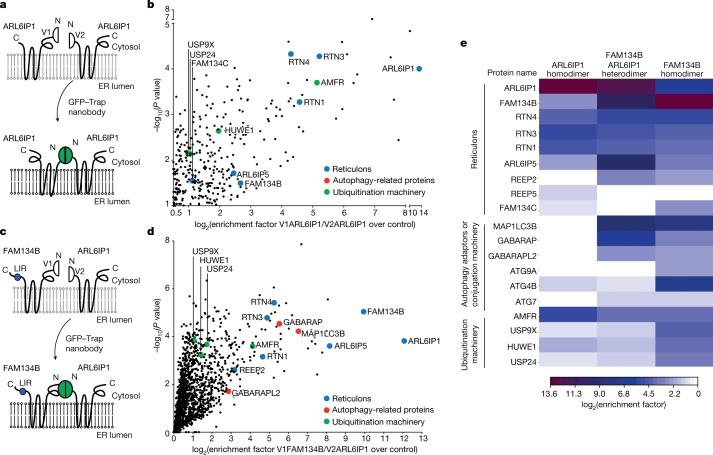
ARL6IP1 supports the formation of a multi-receptor complex with FAM134B and other ER-shaping proteins. **a**, ARl6IP1 N-terminally tagged with the non-fluorescent N-terminal (V1) and ARl6IP1 N-terminally tagged with the C-terminal (V2) fragment of the Venus protein exhibit fluorescence after interaction. **b**, Single-sided Volcano plot of the label-free interactome of ARL6IP1 homodimers revealed that FAM134B, FAM134C and other RHD-containing ER proteins (blue) and proteins of the ubiquitination machinery (green) are binding partners. Only notable hits with log_2_(*P* value) > 1 and –log_10_(*P* value) > 1.3 are labelled in colour (one-sided paired Student’s *t*-test with three biological replicates). **c**, FAM134B N-terminally tagged with the non-fluorescent N-terminal (V1) and ARL6IP1 N-terminally tagged with the C-terminal (V2) fragment of the Venus protein exhibit fluorescence after interaction. **d**, Single-sided volcano plot of the label-free interactome for V1-FAM134B–V2-ARL6IP1 heterodimers include autophagy-related proteins LC3B and GABARAPL2 (red) and FAM134B as known binding partners. Notable hits with log_2_(*P* value) > 1 and –log_10_(*P* value) > 1.3 are labelled in colour (one-sided paired Student’s *t*-test with three biological replicates). Red dots indicate autophagy-related proteins. **e**, Heatmap of log_2_ enrichment of V1-ARL6IP1–V2-ARL6IP1, V1-FAM134B–V2-ARL6IP1 or V1-FAM134B–V2-FAM134B over mock from notable hits indicated in **b** and **d**.

**Extended Data Fig. 3:**
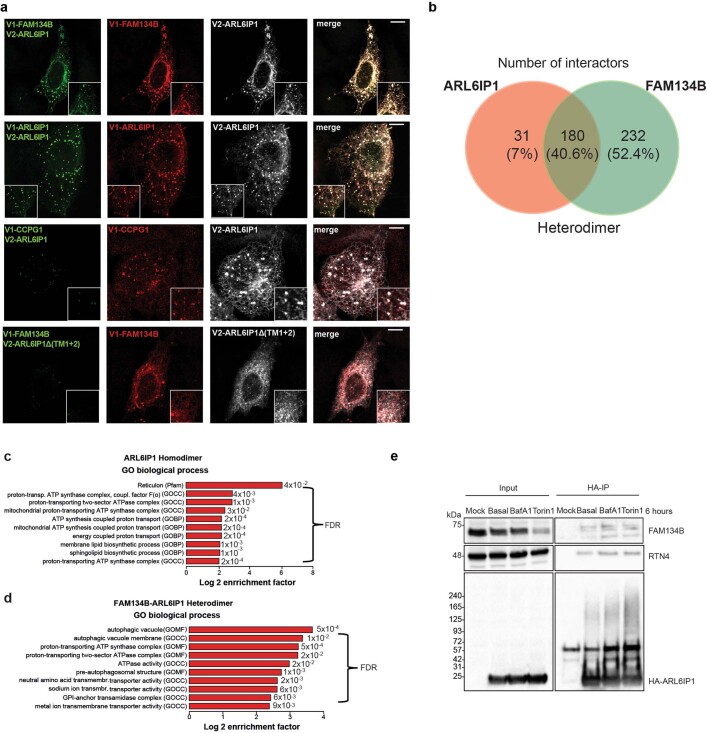
ARL6IP1 forms heterodimers with FAM134B but does not bind LC3B on its own. **a)** V1-ARL6IP1/V2-ARL6IP1 homodimers and V1-FAM134B/V2-ARL6IP1 heterodimers are formed in transiently expressing U2OS cells as evidenced by the Venus fluorescence. Co-transfection of V2-ARL6IP1 with V1-CCPG1, another ER resident protein, which does not interact with ARL6IP1, or a construct encoding an ARL6IP1 variant devoid of the first helical hairpin (V2-ARL6IP1Δ(TM1+2)), which is required for the interaction between FAM134B and ARL6IP1, does not result in heteromerisation as evidenced by the lack of the Venus fluorescence. The single channel images represent immunostaining for either FAM134B, ARL6IP1, and CCPG1 or the Venus signal. 1 experiment. Scale bars: 10 µm. **b)** Venn diagram of interactors of ARL6IP1 and FAM134B homo- and heterodimers, respectively. Numbers represent the identified peptides significantly enriched in three IP and mass spectrometry replicates for each condition. **c,d)** Annotation enrichment analysis of the interactome of V1-ARL6IP1/V2-ARL6IP1 homodimers and V1-FAM134B/V2-ARL6IP1 heterodimers. Bars represent the significantly enriched gene ontology biological process (GOBP), the gene ontology cellular components (GOCC), the gene ontology molecular function (GOMF), and the domain enrichment (Pfam). The Benjamini-Hochberg FDR value is included (right side of the bars). **e)** Western blot analysis showing that RTN4, another ER-shaping protein characterised by the presence of a RHD, and FAM134B co-precipitate with HA-tagged ARL6IP1 (1 exp.).

We next used biomolecular complementation affinity purification (BiCAP)^15^ to study the interactions of ARL6IP1 and FAM134B in vivo (Fig. 3a,c). For this purpose, ARL6IP1 and FAM134B were linked to the V1 or V2 segment of the fluorescent Venus protein. The Venus signals for V1–ARL6IP1 and V2–ARL6IP1 homodimers and for V1–FAM134B and V2–ARL6IP1 heterodimers were distributed along the ER. By contrast, no Venus signal was observed for the non-canonical ER-phagy receptor CCPG1 (ref. ^16^) or for the ARL6IP1 variant lacking TM1 and TM2 (Extended Data Fig. 3a), which did not co-precipitate with FAM134B (Extended Data Fig. 2f). To characterize the functional relevance of this interaction, we immunoprecipitated homodimers and heterodimers and analysed interacting proteins by liquid chromatography and mass spectrometry (LC–MS)^17^ (Fig. 3b,d,e). Within all interacting proteins, 7% exclusively interacted with ARL6IP1 homodimers, 52.4% exclusively with FAM134B homodimers and approximately 40% with both (Extended Data Fig. 3b). Among the top ten gene ontology terms identified for interaction partners of ARL6IP1 homodimers were ER structural components (Extended Data Fig. 3c) such as FAM134B and FAM134C as well as the RHD proteins RTN1, RTN3 and RTN4 (Fig. 3b,e and Extended Data Fig. 3c,e). Notably, the main non-neuronal RTN4 isoform RTN4B was previously identified to interact with FAM134C and to have a role in autophagy^18^. Components of the autophagic vesicle formation machinery such as LC3B and GABARAPL2 were only identified as binding partners of ARL6IP1–FAM134B heterodimers but not ARL6IP1 homodimers (Fig. 3b,d,e and Extended Data Fig. 3c,d). Both ARL6IP1 and FAM134B homodimers interacted with components of the ubiquitination machinery (Fig. 3b,d,e). This included different E3 ligases, such as the ER-resident E3 ligase AMFR (also known as gp78) and HUWE1, and the deubiquitinating enzymes USP9X and USP24. Taken together, our data suggest that heterocomplexes of ARL6IP1 and FAM134B are part of ER-phagy receptor clusters.

## Ubiquitination promotes LC3B binding of FAM134B

**Fig. 4:**
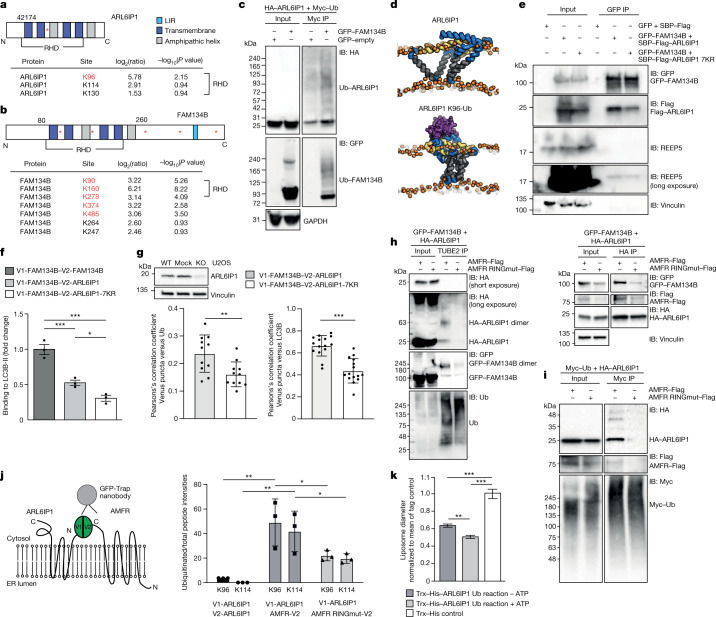
LC3B binding to FAM134B–ARL6IP1 complexes depends on ARL6IP1 ubiquitination. **a**,**b**, Position and log_2_(fold changes) of ubiquitinated lysine residues identified by LC–MS in ARL6IP1 (cells expressing V1-ARL6IP1–V2-ARL6IP1 or V1-FAM134B–V2-ARL6IP1) (**a**) and FAM134B (cells expressing V1-FAM134B–V2-FAM134B or V1-FAM134B–V2-ARL6IP1) (**b**). Significant sites are indicated in red (–log_10_(*P* value) > 1.3, one-sided unpaired Student’s *t*-test). **c**, Anti-Myc IP from HEK293T cells co-expressing HA–ARL6IP1, GFP–FAM134B and Myc–ubiquitin (Ub) confirms ARL6IP1 and FAM134B ubiquitination (one experiment). **d**, Snapshots from coarse-grained molecular dynamics simulations showing the most populated conformations of non-ubiquitinated and ubiquitinated ARL6IP1 (ubiquitin purple) in 1-palmitoyl-2-oleoyl-glycero-3-phosphocholine (POPC 16:0/18:1) bilayers (orange). **e**, Interaction with FAM134B but not endogenous REEP5 is promoted by ARL6IP1 ubiquitination (two experiments). **f**, LC3B co-precipitation with V1-FAM134B–V2-ARL6IP1-7KR is reduced compared with V1-FAM134B–V2-ARL6IP1 (three experiments; one-way ANOVA, *F* = 46; Holm–Sidak’s post-test, FAM134B–FAM134B versus FAM134B–ARL6IP1, *P* = 0.0014; FAM134B–FAM134B versus FAM134B–ARL6IP1-7KR, *P* = 0.0002; FAM134B–ARL6IP1 versus FAM134B–ARL6IP1-7KR, *P* = 0.0242). **g**, Pearson’s correlation analysis for ubiquitin-positive and LC3B-positive Venus puncta for V1-FAM134B–V2-ARL6IP1 and V1-FAM134B–V2-ARL6IP1-7KR in *ARL6IP1* KO U2OS cells (1 experiment with *n* = 11 (ubiquitin) and 15 (LC3B) cells; two-sided unpaired Student’s *t*-test, *P* = 0.006 (ubiquitin) and *P* = 0.0001 (LC3B)). **h**, Left, ubiquitinated ARL6IP1 is detected after TUBE2 pull-down after co-expression of GFP–FAM134B and HA–ARL6IP1 with AMFR–Flag but not AMFR-RINGmut–Flag. Right, ARL6IP1–FAM134B interaction is promoted by AMFR but not AMFR-RINGmut–Flag (*n* = 1). **i**, Myc pull-down after co-expression of Myc–ubiquitin–HA–ARL6IP1 with AMFR–Flag or AMFR-RINGmut–Flag confirms ARL6IP1 ubiquitination with active AMFR (*n* = 1). **j**, Schematic (left) and quantification (right) of LC–MS of GFP pull-downs of co-expressed V1-ARL6IP1 and AMFR-V2 or AMFR-RINGmut-V2: AMFR ubiquitinates ARL6IP1 at K96 and K114 (one-sided unpaired Student’s *t*-test: V1-ARL6IP1–V2-ARL6IP1 versus V1-ARL6IP1–AMFR-V2 K96, *P* = 0.0075; K114, *P* = 0.006; V1-ARL6IP1–AMFR-V2 versus V1-ARL6IP1–AMFR-RINGmut-V2 K96, *P* = 0.038; K114, *P* = 0.044). **k**, In vitro ubiquitination of Trx–His–ARL6IP1 with AMFR decreases the mean liposome diameter in TEM of freeze-fractured liposomes by about 20%. Incubations without ATP served as control (2 experiments with *n* = 393, 346 and 223 vesicles analysed (left to right); two-sided Kruskal–Wallis test with Dunn’s post-hoc test: Trx–His–ARL6IP1 and ATP–Trx–His–ARL6IP1 + ATP, *P* = 0.0024; Trx–His–ARL6IP1 – ATP and control, *P* < 0.0001; Trx–His–ARL6IP1 + ATP and control, *P* < 0.0001). Quantitative data shown as the mean ± s.e.m. Source Data

**Extended Data Fig. 4:**
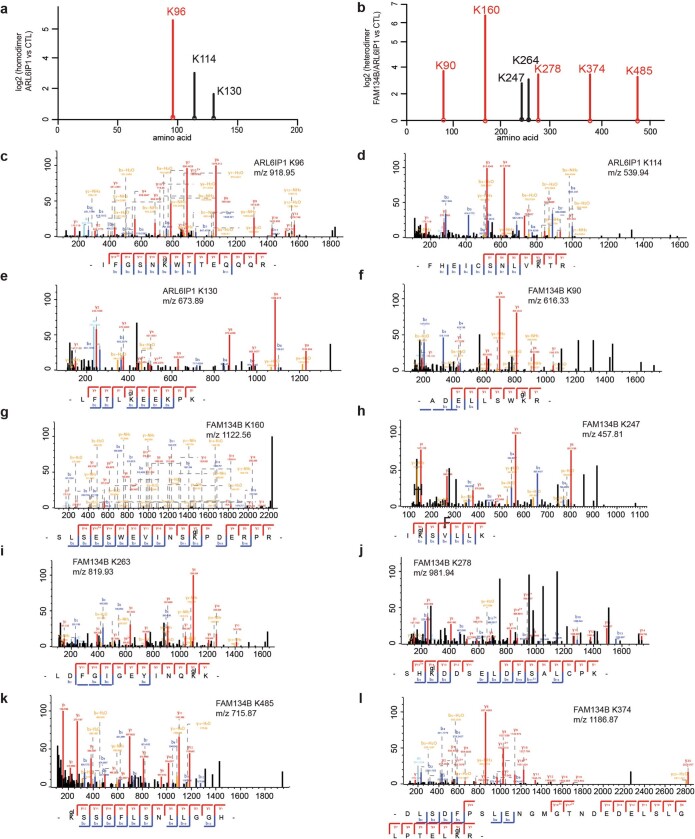
Mass spectrometry analysis of ubiquitinated lysines in ARL6IP1 and FAM134B proteins. **a,b)** Localization of the identified lysines in ARL6IP1 and FAM134B versus Log2 enrichment over mock. Significantly modified lysines (−Log10 >1.3) are highlighted in red. **c–e**) Spectra of modified lysines of ARL6IP1 peptides (K96, K114 and K130) found in ARL6IP1 homo- or heterodimers with FAM134B. **f–l)** Spectra of modified lysines of FAM134B peptides, K90, K160, K374, K278 and K485 found in FAM134B in the heterodimer complex with ARL6IP1.

As AMFR mediates the ubiquitination of FAM134B^17^, we proposed that ubiquitination may also participate in the regulation of ARL6IP1. The LC–MS analysis identified several ubiquitinated lysine residues within ARL6IP1 in both ARL6IP1 homodimers and ARL6IP1–FAM134B heterodimers (Fig. 4a and Extended Data Fig. 4a,c–e). Most of the residues were located close to the RHDs of ARL6IP1, with K96 being significantly ubiquitinated and K114 and K130 potentially ubiquitinated (Fig. 4a). FAM134B exhibited an even higher number of ubiquitinated lysine residues, namely K90, K160, K278, K374 and K485 and potentially K247 and K264, which are also located close to its RHDs^17^ (Fig. 4b and Extended Data Fig. 4b,f–l). Co-immunoprecipitation assays following the overexpression of HA–ARL6IP1 and Myc–ubiquitin in HEK293T cells confirmed that a significant amount of ARL6IP1 is ubiquitinated in the presence of FAM134B (Fig. 4c).

**Extended Data Fig. 5:**
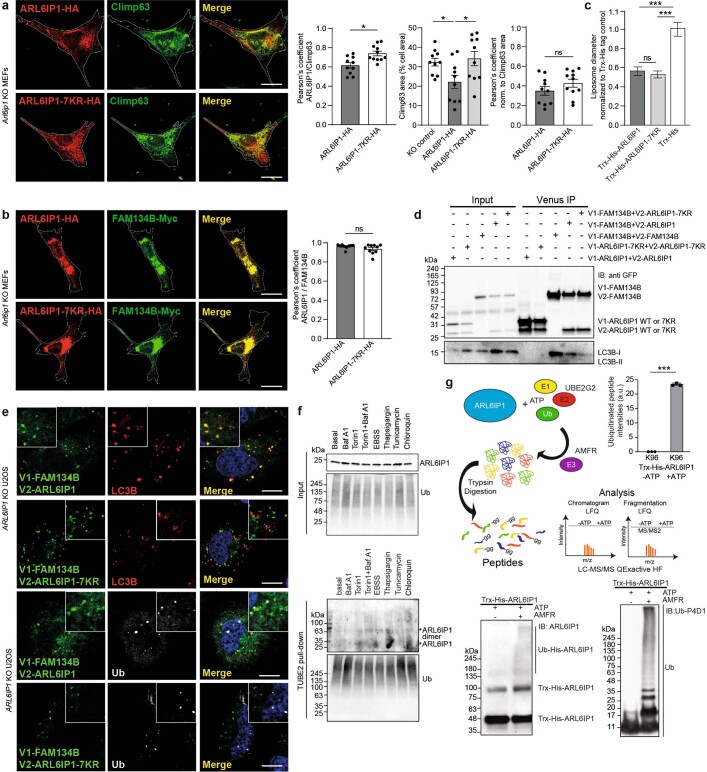
ER-fragmentation (ER-phagy) is promoted by ubiquitination of ARL6IP1. **a)** Co-localisation of either ARL6IP1-HA or ARL6IP1-7KR-HA with Climp63 in *Alr6ip1* KO MEFs (1 exp.; 10 cells per genotype; 2-sided Mann-Whitney-U-test: Pearson’s coefficient ARL6IP1-HA versus ARL6IP1-7KR-HA p = 0.012, Climp63 area control versus ARL6IP1-HA p = 0.036, Climp63 area ARL6IP1-HA versus ARL6IP1-7KR-HA p = 0.036). Scale bars: 10 µm. **b)** Co-localisation of either ARL6IP1-HA or ARL6IP1-7KR with FAM134B-Myc in *Alr6ip1* KO MEFs does not differ (1 exp.; 10 cells per genotype; 2-sided Mann-Whitney-U-test). Scale bars: 10 µm. **c)** Quantitative evaluations of TEM images of freeze-fractured liposomes demonstrate that the shaping properties of His-Trx-ARL6IP1-7KR do not differ from His-Trx-ARL6IP1 (2 exp.; n = 344/391/242; Kruskal-Wallis test with Dunn’s post-hoc test: His-Trx-ARL6IP1 versus His-Trx p < 0.0001, His-Trx-ARL6IP1-7KR versus His-Trx p < 0.0001; n = 344/391/242). **d)** The binding of LC3B to V1-ARL6IP1-7KR/V2-FAM134B heterodimers is reduced compared to V1-ARL6IP1/V2-FAM134B heterodimers (3 exp.). Illustration for quantification shown in Fig. 4f. **e)** The co-localization of ER-fragments with LC3B (red) and Ubiquitin (Ub, white) was analysed in *ARL6IP1* KO U2OS cells transiently expressing either V1-FAM134B/V2-ARL6IP1 or V1-FAM134B/V2-ARL6IP1-7KR heterodimers after treatment with Torin1 for 6 h. Scale bars: 10 µm. Illustration for quantification shown in Fig. 4g. **f)** Endogenous ARL6IP1 is ubiquitinated upon induction of ER stress (1 exp.). U2OS cells were harvested at steady state (basal) or after incubation with the indicated stressors for 6 h (Bafilomycin A1 200 nM, Torin1 250 nM, Thapsigargin 1.5 µM, Tunicamycin 5 µg/ml, Chloroquine 100 µM; stressors and steady state with DMSO 1:1,000). ARL6IP1 is detected in the TUBE2-pull-down. **g)** Upper: The *in vitro* ubiquitination assay with subsequent mass spectrometry shows that AMFR can ubiquitinate ARL6IP1 K96 (1 exp. with 3 replicates; 2-sided unpaired Student’s t-test p = 0.0001). Lower: Confirmation of ubiquitination of ARL6IP1 by immunoblot analysis (1 exp.). Quantitative data are shown as mean ± SEM. Source Data

We next simulated the structural dynamics of non-ubiquitinated ARL6IP1 and the ubiquitinated ARL6IP1 (K96-Ub) embedded in phosphocholine bilayers using coarse-grained molecular dynamics simulations (up to 10 µs). Ubiquitination resulted in a more compact conformation in which the ubiquitin moiety interacted with the cytosolic loops (Fig. 4d). To assess the functional relevance of ARL6IP1 ubiquitination, we replaced all seven predicted ubiquitinated lysine residues with arginine residues (ARL6IP1-7KR). Transiently expressed HA–ARL6IP1-7KR co-precipitated with green fluorescent protein (GFP)-tagged FAM134B (GFP–FAM134B), albeit to a lesser extent (Fig. 4e). A reduced interaction was also evident from co-immunoprecipitation of endogenous FAM134B with HA-ARL6IP1-7KR (Extended Data Fig. 2f). HA-ARL6IP1-7KR also co-localized with the ER sheet marker CLIMP63 (ref. ^19^) (Extended Data Fig. 5a) and with FAM134B–Myc (Extended Data Fig. 5b). Moreover, the shaping properties of ARL6IP1-7KR tagged with a thioredoxin–histidine tag (Trx–His–ARL6IP1-7KR) did not differ from Trx–His–ARL6IP1 in liposome-binding assays (Extended Data Fig. 5c). ARL6IP1-7KR therefore preserves basic functions of ARL6IP1.

Next, we cloned ARL6IP1-7KR and FAM134B constructs with the complementing V1 and V2 segments of the Venus protein. Precipitation with anti-GFP-beads showed an efficient pull-down of V1-FAM134B–V2-ARL6IP1 heterodimers with LC3B. By contrast, binding to LC3B was significantly reduced for complexes with the ubiquitination-deficient ARL6IP1-7KR variant (Fig. 4f and Extended Data Fig. 5d). Co-expression of V1-ARL6IP1-7KR and V2-ARL6IP1 or V1-FAM134B and V2-ARL6IP1 in *ARL6IP1* KO U2OS cells resulted in a regular distribution of the Venus signal in the ER (Extended Data Fig. 5e). Co-localization analysis using Pearson’s correlation showed that Venus puncta co-labelling with ubiquitin or LC3B in *ARL6IP1* KO U2OS cells were reduced for ARL6IP1-7KR (Fig. 4g and Extended Data Fig. 5e).

Because the ER-resident E3 ligase AMFR was found in the interactome of V1-FAM134B–V2-ARL6IP1 complexes, we tested whether AMFR ubiquitinates ARL6IP1. We detected ARL6IP1 and FAM134B in the tandem ubiquitin binding entity (TUBE2) pull-down from cell lysates upon co-expression of GFP–FAM134B and HA–ARL6IP1 together with AMFR–Flag, but not with the catalytically inactive AMFR-C356G-H361A variant (AMFR RINGmut-Flag) (Fig. 4h, left). Moreover, the interaction between ARL6IP1 and FAM134B was promoted by AMFR (Fig. 4h, right). ARL6IP1 was detected in the Myc pull-down assay after co-expression of Myc–ubiquitin and ARL6IP1 with active AMFR, but not with the inactive AMFR RINGmut variant (Fig. 4i). When V1-ARL6IP1 was expressed with AMFR-V2, the ratio of peptides ubiquitinated at K96 or K114 in comparison to total peptides was approximately doubled compared with co-expression with AMFR RINGmut-V2 (Fig. 4j). We further detected ubiquitinated endogenous ARL6IP1 after induction of ER stress (Extended Data Fig. 5f), and we verified that ARL6IP1-K96 was ubiquitinated by AMFR in vitro (Extended Data Fig. 5g).

We considered that ubiquitination of ARL6IP1 might be important for membrane remodelling during ER-phagy. Accordingly, shaping assays showed that in vitro ubiquitination of ARL6IP1 by AMFR resulted in reduced mean liposome diameters (Fig. 4k). We therefore propose that the ubiquitination of RHDs of ARL6IP1 and FAM134B by AMFR is probably involved in ER remodelling during ER-phagy.

## Impaired ER-phagy flux after ARL6IP1 disruption

**Fig. 5:**
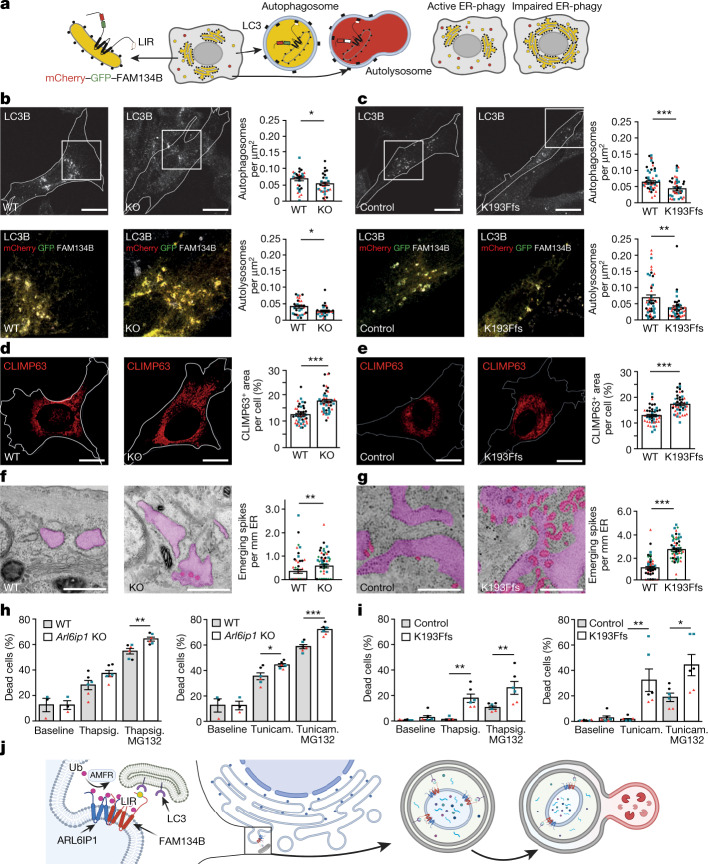
FAM134B-mediated ER-phagy requires ARL6IP1 in mice and humans. **a**, Rational of the mCherry–GFP–FAM134B reporter. **b**,**c**, ARL6IP1 loss-of-function compromises ER-phagy. *Arl6ip1* WT and KO MEFs (**b**) or fibroblasts from the patient and the healthy individual (control) (**c**) were transfected with mCherry–GFP–FAM134B and stained for LC3B. Quantifications of LC3B^+^mCherry^+^GFP^+^ puncta and LC3B^+^mCherry^+^GFP^–^ puncta per cell area suggest that the formation of autophagosomes (3 experiments with 10 cells per genotype each; two-sided Mann–Whitney *U*-test; MEFs, *P* = 0.0479; human cells, *P* = 0.0005) and autolysosomes (3 experiments with 10 cells per genotype each; two-sided Mann–Whitney *U*-test; MEFs, *P* = 0.0307; human cells, *P* = 0.0096) is impaired. **d**,**e**, ARL6IP1 loss-of-function enlarges ER sheets. *Arl6ip1* WT and KO MEFs (**d**) or fibroblasts from the patient and healthy individual (**e**) were stained for the ER sheet protein CLIMP63 and the relative CLIMP63^+^ area per cell calculated (3 experiments with 15 cells per genotype; two-sided Mann–Whitney *U*-test; MEFs, *P* = 0.0001; human cells, *P* = 0.0001). **f**,**g**, TEM images showed increased numbers of small highly curved ER protrusions emanating from ER sheets in the absence of ARL6IP1 (ER sheets in light purple, ER-emerging spikes in pink) in MEFs (**f**) and human fibroblasts (**g**) (1 experiment with 55 cells per genotype; two-sided Mann–Whitney *U*-test; *P* = 0.0059 (**f**) and *P* = 0.0001 (**g**)). **h**,**i**, ARL6IP1 loss-of-function decreases cell viability in the presence of the ER stressors tunicamycin (Tunicam.) or thapsigargin (Thapsig.) with or without the proteasome inhibitor MG132 in MEFs (**h**) and human fibroblasts (**i**) (3 experiments with 2 replicates; two-sided Mann–Whitney *U*-test; MEFs, thapsigargin + MG132, *P* = 0.0022; tunicamycin, *P* = 0.041; tunicamycin + MG132, *P* = 0.0022; patient cells, thapsigargin, *P* = 0.0022; thapsigargin + MG132, *P* = 0.0087; tunicamycin, *P* = 0.0022; tunicamycin + MG132, *P* = 0.026). **j**, Cartoon showing ARL6IP1 (blue) ubiquitinated (red balls) by AMFR in a complex with FAM134B (red), which binds to LC3 (purple) during ER-phagy. Quantitative data are shown as the mean ± s.e.m. Individual experiments are indicated by differently coloured data points. Scale bars, 500 nm (**f**,**g**), 10 µm (**d**,**e**) or 20 µm (**b**,**c**). The models in **a** and **j** were created using BioRender (https://www.biorender.com). Source Data

**Extended Data Fig. 6:**
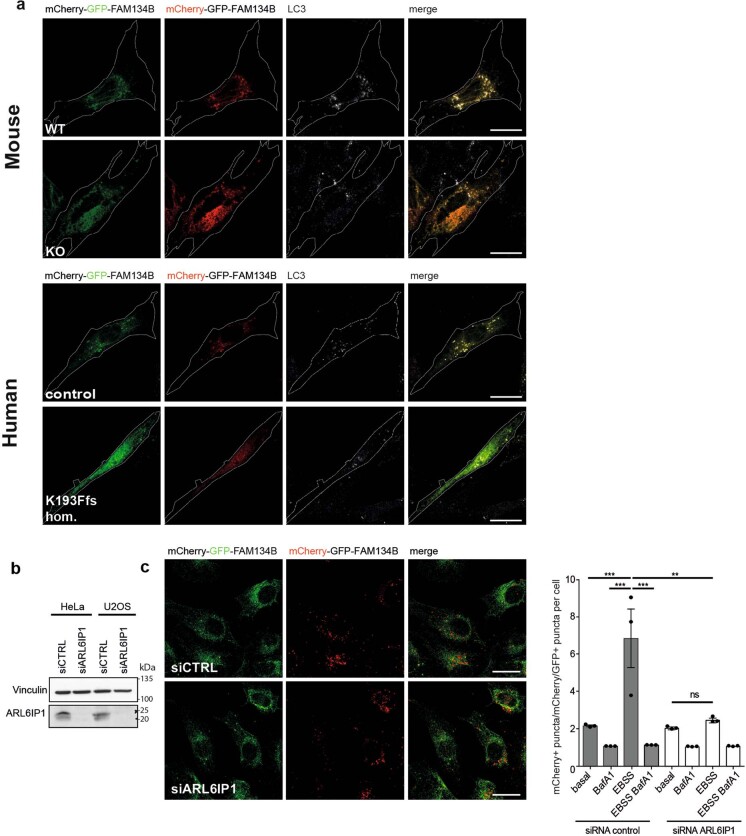
FAM134B-driven ER-phagy is compromised in the absence of ARL6IP1. **a)** Single channels of the merged images displayed in Fig. 5b and c. Scale bar: 20 μm. **b)** Western blot analysis of siRNA mediated knock-down of ARL6IP1 in HeLa and U2OS cells (1 exp. with 2 replicates). **c)** The ratio between mCherry-positive and mCherry and GFP-positive puncta is decreased in EBSS starved cells upon knock-down of ARL6IP1 and induction of the reporter with doxycycline (1 exp. with triplicates; one-way ANOVA with Bonferroni post-hoc analysis, p = 0.0001, F = 12.19: siRNA control basal versus EBSS p = 0.0006; BafA1 versus EBSS p = 0.0001, EBSS versus EBSS BafA1 p = 0.0001; siRNA control EBSS versus siRNA ARL6IP1 EBSS p = 0.0013). Scale bar: 10 μm. Quantitative data are shown as mean ± SEM. Source Data

**Extended Data Fig. 7:**
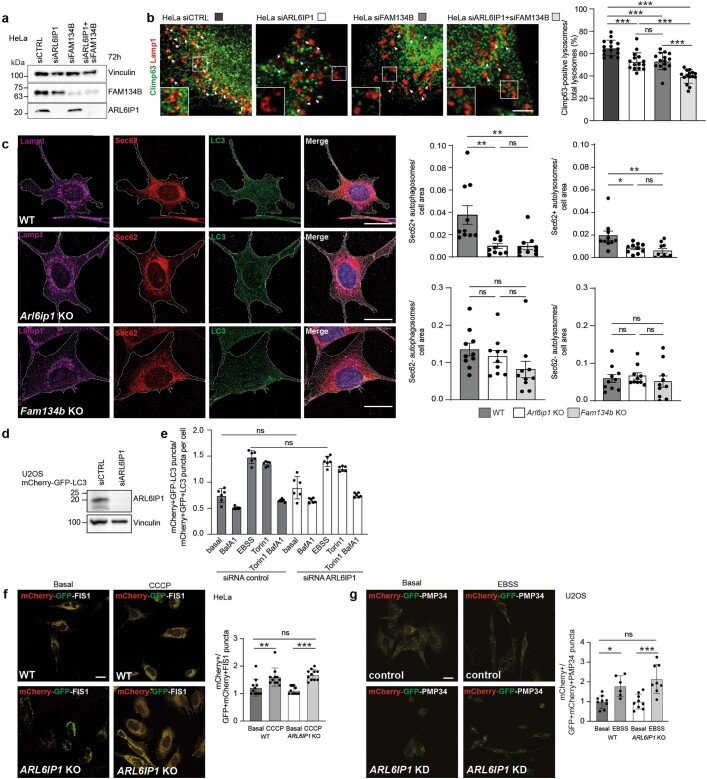
ER-phagy but not bulk autophagy, mitophagy or pexophagy is compromised upon disruption of ARL6IP1. **a)** siRNA mediated knock-down of either ARL6IP1, FAM134B or both in HeLa cells (3 exp.). **b)** The ratio of Climp63-positive versus Climp63-negative lysosomes upon knock-down of either ARL6IP1 or FAM134B is consistent with a defect in ER-phagy. Cells were treated with Torin1 and Bafilomycin A1. The simultaneous knock-down of both further decreases this ratio (1 exp.; 15 cells per genotype, one-way-ANOVA, F-value = 32.59, p = 0.0001, Bonferroni post-hoc analysis: siCTRL versus siARL6IP1 p = 0.0001, siCTRL versus siFAM134B p = 0.0017, siRNA CTRL versus siARL6IP1+siFAM134B p = 0.0001; siARL6IP1 versus siARL6IP1+siFAM134B p = 0.0001, siFAM134B versus siARL6IP1+siFAM134B p = 0.0001). Scale bar: 5 µm. **c)** Arl6ip1 and Fam134b WT and KO MEFs were EBSS starved for 4 h, fixed and stained for Lamp1, LC3B, and the ER-protein Sec62. The quantification of autophagosomes (LC3B-positive and Lamp1-negative) loaded with ER (Sec62-positive) or devoid of ER (Sec62-negative) supports a defect in ER-phagy but not bulk autophagy (1 exp.; 10 cells per genotype were analysed; one-way-ANOVA with Bonferroni post-hoc analysis; autophagosomes: p = 0.001, F-value = 9.081, WT versus *Arl6ip1* KO p = 0.003, WT versus *Fam134b* KO p = 0.003; autolysosomes: p = 0.004, F-value = 6.954, WT versus *Arl6ip1* KO p = 0.024, WT versus *Fam134b* KO p = 0.005). Scale bar: 20 µm. **d)** Knock-down of ARL6IP1 in U2OS cells with inducible expression of the mCherry-GFP-LC3 reporter (1 exp.). **e)** Bulk autophagy upon siRNA mediated knock-down of ARL6IP1 is not compromised in U2OS cells expressing the mCherry-GFP-LC3 reporter (1 exp. with 3 replicates, one-way-ANOVA with Bonferroni post-hoc analysis). **f)** Mitophagy is not affected by KO of ARL6IP1. WT and *ARL6IP1* KO HeLa cells were transfected with the mitophagy reporter mCherry-GFP-FIS1. Mitophagy flux was studied at steady state (basal) and after 4 h with 40 µM CCCP (1 exp; n = 11 cells each were analysed; one-way ANOVA with Bonferroni post-hoc analysis, F-value = 13.36, p = 0.0001: WT basal versus CCCP p = 0.0067, KO basal versus CCCP p = 0.0001). Scale bar: 25 µm. **g)** Pexophagy is not affected by ARL6IP1 knock-down. The mCherry-GFP-PMP34 reporter was induced in control and ARL6IP1 knock-down U2OS cells and pexophagy flux studied at steady state (basal) and 20 h of EBSS starvation (1 exp. with n = 9/6/9/8 cells analysed; one-way ANOVA with Bonferroni post-hoc analysis, F-value = 10.65, p = 0.0001: WT basal versus CCCP p = 0.0377, KO basal versus CCCP p = 0.0004). Scale bar: 25 µm. Quantitative data are shown as mean ± SEM. Source Data

**Extended Data Fig. 8:**
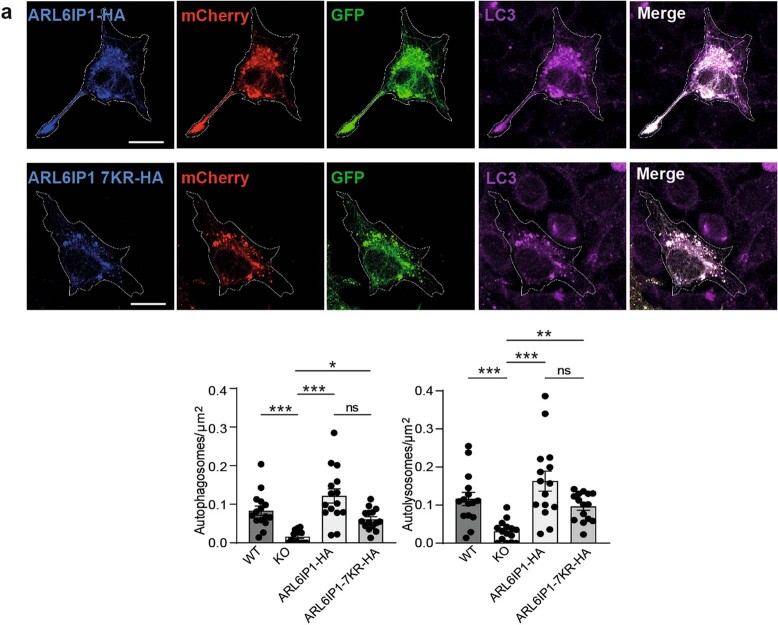
Rescue experiment with either ARL6IP1 or the ARL6IP1-7KR variant. **a)** In *Arl6ip1* KO MEFs ER-phagy can be rescued by overexpression of either ARL6IP1 or ARL6IP1-7KR-HA. KO MEFs were transfected with the mCherry-GFP-FAM134B reporter and either ARL6IP1-HA or ARL6IP1-7KR-HA, fixed and stained for LC3B. Quantifications of LC3B/mCherry/GFP-positive puncta (autophagosomes) and LC3B/mCherry-positive but GFP-negative puncta (autolysosomes) per cell area (1 exp. with 15 cells analysed per genotype; Kruskal-Wallis test with Dunn`s multiple comparison test: autophagosomes WT versus KO p = 0.0002, KO versus ARL6IP1 rescue p = 0.0001, KO versus ARL6IP1-7KR rescue p = 0.0138; autolysosomes WT versus KO p = 0.001, KO versus ARL6IP1 rescue p = 0.0001, KO versus ARL6IP1-7KR rescue p = 0.0053). Scale bars: 20 µm. Quantitative data are shown as mean ± SEM. Source Data

To assess whether FAM134B-mediated ER-phagy is compromised in the absence of ARL6IP1, we overexpressed the mCherry–GFP–FAM134B reporter in WT and *Arl6ip1* KO MEFs (Fig. 5a). Staining for LC3B enabled the quantification of autophagosomes and autolysosomes because GFP is quenched in the acidic lumen of autolysosomes. Puncta labelled for LC3B, mCherry and GFP (autophagosomes) and puncta positive for LC3B and mCherry but negative for GFP (autolysosomes) were decreased in KO MEFs and in patient fibroblasts (Fig. 5b,c and Extended Data Fig. 6a). A defect in ER-phagy was further confirmed following siRNA-mediated knockdown of *ARL6IP1* in U2OS cells expressing the mCherry–GFP–FAM134B reporter after induction with doxycycline (Extended Data Fig. 6b,c). Similar results were also obtained after knockdown of *ARL6IP1* or *FAM134B* in HeLa cells alone or in combination, when the defect was more severe (Extended Data Fig. 7a,b). The quantification of SEC62-positive autophagosomes and autolysosomes in either starved *Arl6ip1* or *Fam134b* KO MEFs further confirmed a defect in ER-phagy. By contrast, SEC62-negative autophagosomes and autolysosomes as a readout for bulk autophagy were not affected (Extended Data Fig. 7c). Intact bulk autophagy was additionally verified using the mCherry–GFP–LC3 reporter after knockdown of *ARL6IP1* in U2OS cells (Extended Data Fig. 7d,e). Mitophagy (Extended Data Fig. 7f) and pexophagy (Extended Data Fig. 7g) were not affected following the disruption of ARL6IP1. Notably, ER-phagy in *Arl6ip1* KO MEFs was rescued by ARL6IP1 or ARL6IP1-7KR overexpression (Extended Data Fig. 8).

**Extended Data Fig. 9:**
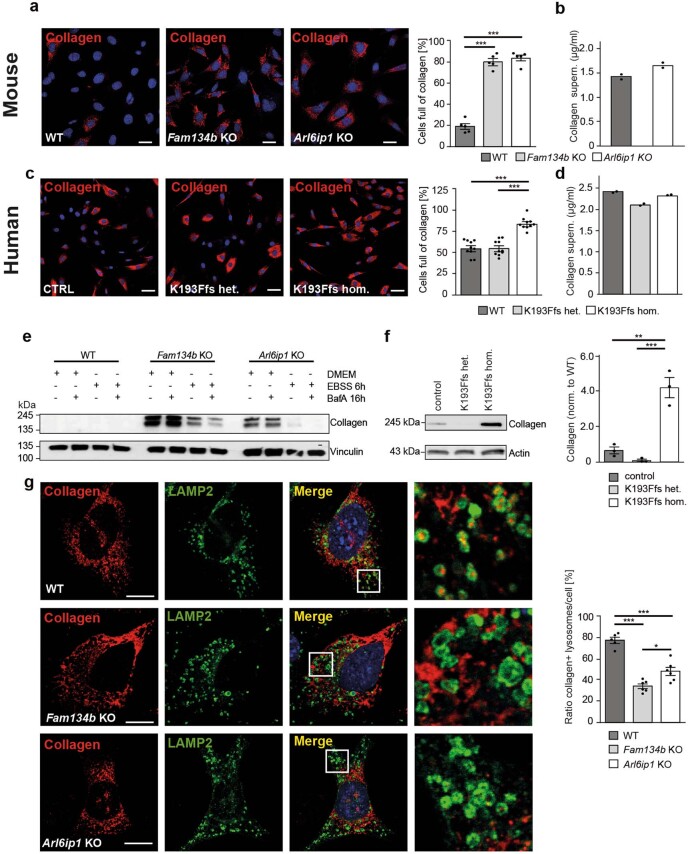
Intracellular accumulation of collagen in the absence of ARL6IP1. **a)** The ratio of cells strongly labelling for collagen is increased in both *Fam134b* and *Arl6ip1* KO MEFs (1 exp. with n = 86 WT cells, n = 121 FAM134B KO MEF cells, n = 69 ARL6IP1 KO MEFs analysed; one-way ANOVA with Bonferroni post-hoc analysis, F-value = 171.7, p = 0.0001: WT versus *Fam134b* KO p = 0.0001, WT versus *Arl6ip1* KO p = 0.0001). Scale bar: 50 µm. **b)** Comparable collagen levels in the supernatants of *Fam134b* and *Arl6ip1* KO MEFs exclude a defect of collagen secretion (1 exp. with n = 2 samples). **c)** The ratio of cells strongly labelling for collagen is increased in fibroblasts of the patient homozygous for the K193Ffs variant (n = 3 experiments with 344 (control), 310 (K193Ffs het.), 399 (K193Ffs hom.) cells analysed; one-way ANOVA with Bonferroni post-hoc analysis, F = 40.02, p = 0.0001: WT versus K193Ffs hom. p = 0.0001, K193Ffs het. versus K193Ffs hom. p = 0.0001). **d)** Comparable collagen levels in the supernatants of fibroblasts of a healthy control, the heterozygous father and the affected patient exclude a defect of collagen secretion (1 exp. with n = 2 samples). **e)** Western blot analysis of WT, *Fam134b* KO and *Arl6ip1* KO MEFs at steady state and EBSS starvation with or without Bafilomycin A1 (BafA). The accumulation of collagen is not enhanced upon Bafilomycin A1 treatment. This suggests that the removal of misfolded collagen by ER-phagy is abolished in *Arl6ip1* KO MEFs. Vinculin served as loading control (1 exp.). **f)** Accumulation of collagen in patient fibroblasts. Actin served as loading control (3 experiments; one-way ANOVA with Bonferroni post-hoc analysis, F-value = 40.8, p = 0.0003: WT versus K193Ffs hom. p = 0.0011; K193Ffs het. versus K193Ffs hom. p = 0.0005). **g)** MEFs devoid of Fam134b or Arl6ip1 cannot deliver collagen to lysosomes. Quantification of collagen-positive lysosomes per cell in cells loaded with collagen (1 exp., 6 cells analysed with 564 (WT), 474 (*Fam134b* KO), 785 (*Arl6ip1* KO) puncta counted; one-way ANOVA with Bonferroni post-hoc analysis, F-value 47.59, p = 0.0001: WT versus *Fam134b* KO p = 0.0001, WT versus *Arl6ip1* KO p = 0.0001, *Fam134b* KO versus *Arl6ip1* KO p = 0.0165). Scale bars: 20 µm. Quantitative data are shown as mean ± SEM. Source Data

Because FAM134B is involved in the lysosomal clearance of ER subdomains containing ERAD-resistant misfolded proteins such as pro-collagen^20^, we also assessed whether the clearance of pro-collagen is affected following disruption of ARL6IP1. The ratio of cells with an intracellular accumulation of pro-collagen was substantially increased in both *Fam134b* and *Arl6ip1* KO MEFs (Extended Data Fig. 9a). A defect in collagen exocytosis could be excluded because collagen concentrations in cell culture supernatants did not differ between genotypes (Extended Data Fig. 9b). Comparable results were obtained for fibroblasts from the patient and from the healthy individual (Extended Data Fig. 9c,d), which were confirmed by immunoblot analysis (Extended Data Fig. 9e,f). Co-staining with LAMP2 further indicated that the delivery of pro-collagen to lysosomes is impaired in *Fam134b* and *Arl6ip1* KO MEFs (Extended Data Fig. 9g).

Collectively, these data suggest that FAM134B-mediated ER-phagy requires ARL6IP1 and that it is likely to be promoted by ARL6IP1 ubiquitination.

## Abnormal ER sheets after ARL6IP1 disruption

**Extended Data Fig. 10:**
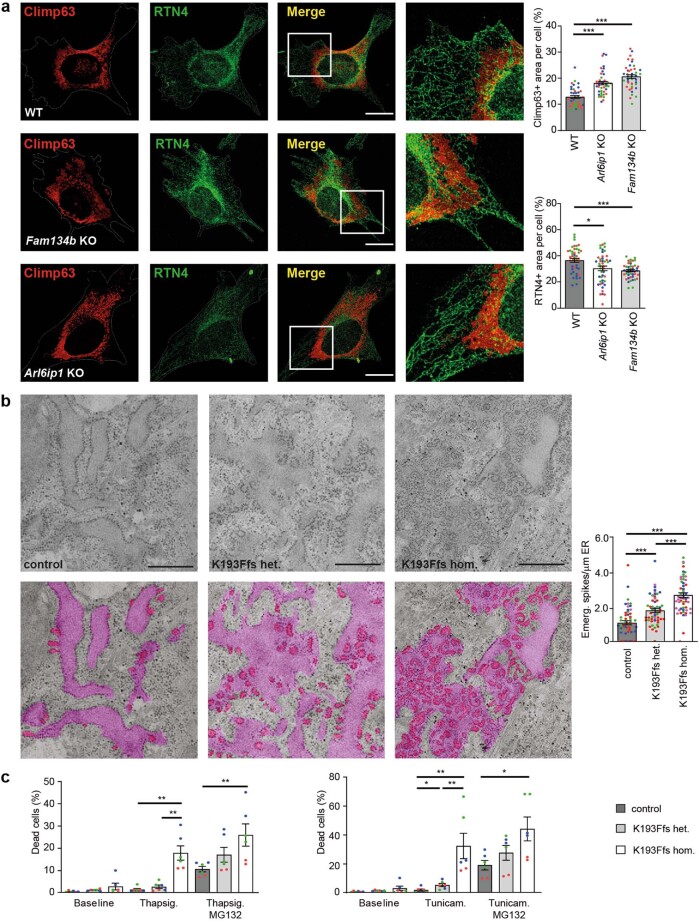
Consequences of compromised FAM134B-dependent ER-phagy for ER structure and cell viability. **a)** Staining for the ER sheet protein Climp63 and the ER tubule marker RTN4 in WT and *Fam134b* and *Arl6ip1* KO MEFs. Relative Climp63-positive or RTN4-positive pixels per cell and their ratio was calculated (3 exp. with 45 cells analysed per genotype; Kruskal-Wallis test with Dunn’s multiple comparisons test: Climp63 WT versus *Arl6ip1* KO p = 0.0001, WT versus *Fam134b* KO p = 0.0001; RTN4 WT versus *Arl6ip1* KO p = 0.036, WT versus *Fam134b* KO p = 0.0005; data points obtained within the same experiment are indicated by the same colour). Scale bar: 10 µm. **b)** Compared with the strongly increased number of small highly curved ER protrusions emanating from ER sheets in fibroblasts of the homozygous patient, fibroblasts of the heterozygous father show an intermediate phenotype (1 exp. with 55 cells per genotype; one-way ANOVA with Bonferroni post-hoc analysis, F-value 42,59, p = 0.0001: WT versus K193Ffs het. p = 0.0002, WT versus K193Ffs hom. p = 0.0001, K193Ffs hom. versus K193Ffs het. p = 0.0001; data points obtained within the same experiment are indicated by the same colour). Scale bar: 500 nm. The ER is shown in light purple colour. ER-emerging spikes are shown in pink. **c)** In the presence of the ER stressor Tunicamycin without the proteasomal inhibitor MG132 the viability of the fibroblasts of the heterozygous father is slightly increased compared to control (3 experiments with 2 replicates each; 2-sided Mann-Whitney-U-test; Thapsigargin: WT versus K193Ffs hom. p = 0.0022, K193Ffs het. versus K193Ffs hom. p = 0.0022; Thapsigargin + MG132: WT versus K193Ffs hom. p = 0.0087; Tunicamycin: WT versus K193Ffs het. p = 0.0411, WT versus K193Ffs hom. p = 0.0022; K193Ffs het. versus K193Ffs hom. p = 0.0022; Tunicamycin+MG132: WT versus K193Ffs hom. p = 0.026). Individual experiments are indicated by differently coloured data points. Quantitative data are shown as mean ± SEM. Source Data

In agreement with a severe defect of FAM134B-dependent ER-phagy flux, the relative area covered by CLIMP63-labelled ER sheets was increased in maximum intensity projections of confocal *z*-stacks of *Arl6ip1* KO MEFs (Fig. 5d) and in patient fibroblasts (Fig. 5e). The area labelled for RTN4, a marker of ER tubules^21^, was decreased, which closely resembled findings for *Fam134b* KO MEFs (Extended Data Fig. 10a).

Transmission electron microscopy (TEM) analyses revealed an increased number of spike-like ER protrusions in *Arl6ip1* KO MEFs (Fig. 5f). The numbers of spike-like ER protrusions was higher in fibroblasts from the patient compared with those from the healthy individual (Fig. 5g). Notably, such ER protrusions were also increased in fibroblasts from the patient’s father (heterozygous for K193Ffs) compared with the healthy individual, but significantly less compared with the patient (Extended Data Fig. 10b).

The compromised ER structure was accompanied by diminished cell viability in response to ER stressors such as thapsigargin or tunicamycin with or without the proteasome inhibitor MG132 in KO MEFs (Fig. 5h) and in fibroblasts from the patient (Fig. 5i). By contrast, the viability of fibroblasts from the patient’s father only differed marginally from control cells (Extended Data Fig. 10c). Notably, the alterations in ER structure were more pronounced in human fibroblasts compared with MEFs, whereas the sensitivity was less for human fibroblasts in our cell viability assays. This disparity may be explained by species-specific differences or differences between embryonic and adult tissues. Overall, we showed that the disruption of ARL6IP1 leads to a defect in ER remodelling and ER structure and decreases resistance to ER stress.

We conclude that the loss of ARL6IP1 crucially impairs ER-phagy and decreases cellular fitness. As an in vivo correlate of defective ER-phagy, ER sheets were expanded in DRG neurons in *Arl6ip1* KO mice, which degenerated over time and led to sensory loss. In agreement, *ARL6IP1* knockdown was reported to result in the expansion of ER sheets in HeLa cells^22^ and the fragmentation of ER tubules in *Drosophila* axons^23^. Here we showed that ARL6IP1 is required for FAM134B-mediated ER-phagy. However, it does not act as an ER-phagy receptor on its own (Fig. 5j) because it cannot bind LC3 proteins itself. Instead, ARL6IP1 forms multi-protein clusters with FAM134B and other ER-shaping proteins. Notably, the RHDs of FAM134B and ARL6IP1 were ubiquitinated within such ER-phagy competent multi-protein clusters. Moreover, the ubiquitination of ARL6IP1 increased its shaping properties and its binding to LC3 by FAM134B. Our observation that membrane protrusions emanating from ER sheets are increased suggests that ER membrane remodelling is incomplete in the absence of ARL6IP1. As a new mechanism, we propose that ER-phagy is controlled by the ubiquitin-dependent formation of heteromeric ER-phagy receptor complexes. Ubiquitination therefore acts as a higher level of regulation acting on top of RHD phosphorylation to promote FAM134B oligomerization and ER fragmentation^24^. As ER-phagy is also involved in the degradation of ER subdomains containing ERAD-resistant misfolded proteins, which has been shown for ATZ polymers^25^ and for endogenous pro-collagen^20^, compromised ER-phagy following the disruption of either FAM134B or ARL6IP1 coupled with age-associated attenuation of autophagy might contribute to an accumulation of misfolded or aggregated proteins within the ER. Subsequently, this leads to impaired proteostasis and progressive neurodegeneration. Because ARL6IP1 also binds to FAM134A and FAM134C, two broadly expressed close homologues of FAM134B^2^, which also serve as ER-phagy receptors^26^, this may explain why mutations in *ARL6IP1* lead to more severe disease^2,3,8,9,27^. In light of the evolutionarily conserved function of FAM134B, the formation of heteromeric clusters of ubiquitinated membrane-shaping proteins to remodel the ER may represent a more general principle of cell homeostasis.

## Methods

To study the function of ARL6IP1 and the consequences of its disruption, we investigated *Arl6ip1* KO mice and different cell culture models, including fibroblasts from a patient carrying the homozygous mutation *AR6IP1*^*K193Ffs*^, from the patient’s father (unaffected and carrying the heterozygous allele) and from a healthy individual. Mouse experiments were performed on a C57BL/6 background after backcrossing for more than four generations. Mice were maintained in groups of up to 3 mice per cage at 21 ± 2 °C, air humidity of ≥45%, 15-fold air exchange, 14–10 h day–night cycle and maximum 500 lx. Mice had free access to standard mouse chow and water. Littermates of the same sex were randomly assigned to experimental groups. Experiments were conducted in a blinded manner with regard to cell, mouse and human genotypes. Figure legends include details of replicate experiments used to generate datasets. All animal experiments were approved by the Thüringer Landesamt für Lebensmittelsicherheit und Verbraucherschutz (registration numbers 02-055/14 and UKJ-17-006). Studies using human fibroblasts were approved by the local ethics committee.

Plasmids are presented in Supplementary Table 1. cDNAs were cloned into the pDONR223 vector using a BP Clonase Reaction kit (Invitrogen, 11789100) and further recombined into the Gateway destination vectors pcDNA5-FRT/TO-N-mCherry-EGFP, pcDNA3.1-N-HA, pHAGE-GFP, pcDNA3.1-N-Flag, pcDNA3.1-C-Flag, pcDNA3.1-N-SBP-Flag, pGEX6-GST, and the biomolecular complementation affinity purification system vectors pDEST-V1-ORF, pDEST-V2-ORF, pDEST-ORF-V1 and pDEST-ORF-V2 using a LR Clonase Reaction kit (Invitrogen). Plasmids encoding untagged ARL6IP1, Trx–His-tagged ARL6IP1 and Trx–His–ARL6IP1-7KR were generated by subcloning ARL6IP1 from pGEX6P1 into pPal7 and pET32a, respectively. ARL6IP1 in pGEX6P1 was cloned by PCR using pClneo-ARL6IP1 WT-GFP as template. The three sgRNA guides of ARL6IP1 were cloned into the pLentiCRISPR v2 vector.

Primers are presented in Supplementary Table 2, primary antibodies in Supplementary Table 3 and secondary antibodies are presented in Supplementary Table 4.

### Generation of *Arl6ip1* KO mice

The EUCOMM embryonic stem cell clone HEPD0752_7_A11 (Source Bioscience) was injected into C57BL/6J donor blastocysts. Next, 15–30-week-old F_1_ female offspring from C57BL/6J and CBA/J matings served as foster mice. Resulting chimeras were mated with C57BL/6J. For all experiments, littermates were used, which had been backcrossed for at least four generations. Genotyping was performed by PCR with three primers (*Arl6ip1*-forward: 5′-GTAATATTCTGAGCACTGCCT-3′, *Arl6ip1*-KO-reverse: 5′-TGCCATAATGACCTAATACTGTTGTG-3′, *Arl6ip1*-WT-reverse: 5′-CTAAGCACAGGCTATGAACC-3), which produced a WT band of 537 bp or the KO band of 350 bp.

### Generation of *ARL6IP1* CRISPR–Cas9 KO cell lines

The *ARL6IP1* knockout U2OS cell line was generated using a lentiviral CRISPR–Cas9 system. sgRNAs are reported in Supplementary Table 5 (design at https://portals.broadinstitute.org/gpp/public/analysis-tools/sgrna-design). The lentiviral plasmids were generated as previously reported^28^. The forward and reverse oligonucleotides were annealed and phosphorylated using T4 polynucleotide kinase (BioLabs). The oligonucleotides were ligated into the Cas9 vector pLenti-Puro-v2 and pLenti-Puro-EGFP using the BsmBI site. The lentiviral plasmids were co-transfected into HEK293T cells together with the packaging vectors pPAX2 and pDM2.G for lentivirus production. After 48 h, the medium containing lentiviral particles was collected, centrifuged to remove dead cells and stored at –80 °C. To generate the *ARL6IP1* KO cell line, fresh U2OS or HeLa cells were infected with lentiviral particles with the three different sgRNAs for 48 h and then selected using 5 μg ml^–1^ of puromycin. The surviving cells were maintained in DMEM supplemented with 2 μg ml^–1^ puromycin. When using pLenti-Puro-EGFP as backbone of the sgRNA, cells were also FACS-sorted for GFP expression (SONY SH800S Cell Sorter, version 2.1.6). KO was verified by western blotting.

### Motor performance

For the beam walk test, mice were placed on an elevated beam of 1 m in length and 4 cm in width, with the home cage at the end. After habituation on three consecutive days, the mouse was videotaped from behind during its movement on the beam. The foot base angle of the hind limb was measured at the moment when the toe was lifted.

For the grip strength analysis, mice were lifted at the tail base, brought to a trapeze-shaped handle connected with a force sensor (Grip Strength Meter, Ugo Basile). When the mouse spontaneously grabbed the handle, the mouse was gradually pulled away from the handle until it was released.

For the electrophysiological analysis of peripheral nerves, anaesthetized mice (100 mg kg^–1^ ketamine and 16 mg kg^–1^ xylazine) were placed on a heating pad. One pair of needle electrodes with a tip distance of 5 mm (WE30030.1H10, Science Products) was inserted near the base of the tail and a second pair 30 mm distal to the stimulation site close to the tip of the tail. For the analysis of motor fibres, the stimulus was applied through the proximal electrodes and the response recorded using the distal electrodes. Compound muscle action potentials and sensory nerve action potentials were evoked with increasing intensity (0–15 V, increment of 1 V, 50 µs duration, interstimulus interval of 20 s). Sum action potentials were filtered (high-pass filter 3 Hz, low-pass filter 1.3 kHz) and digitized with a sampling frequency of 10 kHz. Amplitudes were determined from peak to peak.

### Histology, neuron count and TEM of mouse tissues

Mice were deeply anaesthetized and transcardially perfused with PBS (pH 7.4) followed by 4% paraformaldehyde (PFA) in PBS for 10 min. After dissection, tissues were post-fixed in 4% PFA in PBS for at least 1 h. Tissues were incubated in sucrose (10% sucrose for 4 h and in 30% sucrose overnight at 4 °C), frozen on dry ice and cut with a sliding microtome (Leica SM 2000R) in 30-µm-thick free-floating sections and stored in PBS supplemented with sodium azide at 4 °C until further use. For NeuN staining, free-floating sections were permeabilized with 0.25% Triton X-100, blocked in 5% normal goat serum (NGS) for 1 h and incubated with mouse anti-NeuN (Millipore) 1:500 at 4 °C overnight. After washing, sections were incubated with the appropriate secondary antibodies (Invitrogen) at 1:1,000 and Hoechst 33342 (Thermo Scientific). Sections were mounted with Fluoromount-G (Southern Biotech). Images were acquired using Cellobserver Z1 (Zeiss) with the tile-scan module and further analysed using ImageJ.

For paraffin embedding, the samples were dehydrated overnight in a series of ethanol and xylol baths (Leica TP20 Tissue Processor), embedded with paraffin (Leica HistoCore Arcadia) and cut into 5 µm sections with a microtome (ThermoScientific Microm HM 355S). For histological analyses, sections were either stained with haematoxylin and eosin (Sigma-Aldrich) or cresyl-violet (Sigma-Aldrich). Images were acquired using a Zeiss AxioLab A1 microscope and further analysed using ImageJ.

For TEM of tissue sections, animals were perfused with 2.5% glutaraldehyde in PBS unless indicated otherwise. For the analysis of DRGs, mice were perfused with 4% PFA and 2% glutaraldehyde in PBS. After dissection, tissues were post-fixed overnight. Tissues were contrasted with 1% osmium tetroxide, dehydrated and infiltrated with epoxy resin. Ultrathin sections were stained with uranyl acetate and lead citrate, mounted on copper grids and viewed with a Philips CM10 or Zeiss EM 900 digital (DRGs) transmission electron microscope.

### TEM of cultured cells

MEFs and human fibroblasts were fixed by adding an equal volume of double strength fixative (4% PFA, 5% glutaraldehyde in 0.1 M sodium cacodylate buffer, pH 7.4) to the culture medium and incubated for 20 min at room temperature. The fixative mixture was then replaced with one volume of single strength fixative (2% PFA and 2.5% glutaraldehyde in 0.1 M sodium cacodylate buffer, pH 7.4) for another 2 h at room temperature. After 5 washes with 0.1 M sodium cacodylate buffer (pH 7.4), cells were processed for dehydration and embedding in Epon resin^29^. To preserve their original morphology, the monolayer culture of fibroblasts of the patient and of his father were embedded in their original position in their culture flasks. By contrast, WT and *Arl6ip1* KO MEFs were scraped into 2% low-melting-point agarose before the dehydration process and the embedding in Epon resin, as previously described^29^. Subsequently, 70 nm ultrathin sections were cut using a Leica EM UC7 ultra microtome (Leica Microsystems) and stained with uranyl acetate and lead citrate^29^. Cell sections were analysed using an 80 kV transmission electron microscope CM100bio TEM (FEI).

### Skeletal muscle fibre bundle staining

Muscles freshly dissected from 2-month-old mice were fixed in 2% PFA for 15 min and subsequently washed with PBS. Fibre bundles were prepared and used for further analyses. After overnight permeabilization with 0.2% Triton X-100 in PBS, samples were blocked with 5% NGS for 1 h followed by an incubation with α-bungarotoxin-Alexa 555 (Invitrogen) 1:500 and mouse anti-NF200 overnight at 4 °C. After washing with PBS, single myofibre bundles were incubated with the corresponding secondary antibodies (Invitrogen) in a dilution of 1:1,000 for 1 h at room temperature. Nuclei were stained with Hoechst 33258 (Invitrogen). Myofibres were washed with PBS and mounted using Fluoromount-G (Southern Biotech). Images were acquired using a Zeiss LSM 880 confocal microscope with Airyscan using the *z*-stack module. *Z*-projections with maximum intensities processed using ImageJ are shown.

### Cell culture

HEK293T, U2OS and HeLa cells were obtained from the American Type Culture Collection. Their identities were authenticated by STR analysis. U2OS TRex cells were provided by S. Blacklow (Brigham and Women’s Hospital and Harvard Medical School), HeLa TRex were provided by S. Taylor (Manchester University). WT, *Fam134b* KO and *Arl6ip1* KO MEFs were isolated from embryos and immortalized using SV40 large T antigen. All cell lines were regularly tested for mycoplasma contamination using a LookOut Mycoplasma PCR Detection kit (Sigma). Cells were maintained at 37 °C with 5% CO_2_ in DMEM medium (Gibco) supplemented with 10% FBS (Gibco) and 100 U ml^–1^ penicillin and streptomycin (Gibco).

Inducible cell lines were induced with 1 µg ml^–1^ doxycycline (Sigma-Aldrich).

Bafilomycin A1 (LC-Laboratories) was used at a concentration of 200 ng ml^–1^, Torin1 (LC-Laboratories) at 250 nM. EBSS medium was obtained from Gibco. For each treatment, cells were plated the day before to perform the experiments when cells had a confluence of 50–60%. For transient expression, DNA plasmids were transfected using GeneJuice (Merck-Millipore), Turbofect (Thermo Scientific) or Lipofectamine 2000 (Invitrogen).

U2OS TRex cell lines were used to generate stable cell lines using the Flp-IN TRex system (Invitrogen) or the lentiviral vector p-Lenti N-HA. For *ARL6IP1* knockdown experiments, the respective cells were transfected with either 30 pmol siNT (non-targeting sequence, Qiagen) or with 30 pmol double-stranded *ARL6IP1* siRNA (Integrated DNA Technologies; hs.RiARL6IP1.13.2) using Lipofectamine RNAiMAX transfection reagent (Invitrogen, 13778075). For *ARL6IP1* and *FAM134B* double-knockdown experiments, HeLa cells were transfected with *ARL6IP1* siRNA and *FAM134B* siRNA (siRNA RETREG1 18 J-016936-18-0002 and siRNA RETREG1 21 J-016936-21-0002, respectively). Experiments were performed 72 h after transfection.

### Protein isolation from cells and tissue lysates

Cells were collected and lysed in RIPA buffer (50 mM Tris-HCl, pH 7.4, 150 mM NaCl, 1% (v/v) NP-40, 1% (w/v) sodium deoxycholate, 0.1% (w/v) SDS, 1 mM EDTA and complete protease inhibitor (Roche)). Tissue lysates were prepared using a Ultra-Turrax T8 tissue homogenizer (IKA-WERKE) in RIPA buffer. After sonication, homogenates were spun down at 16,900*g* to remove nuclei and insoluble debris. The supernatant was stored at –80 °C until further use.

### Western blotting

Proteins were denatured at 90 °C for 5 min in Laemmli buffer, resolved by SDS–PAGE and transferred to methanol-activated PVDF membranes (Amersham Hybond P 0.45 µm). Membranes were blocked for 1 h in 10% skim milk in TBS-T (Tris-buffered saline with Tween, 20 mM Tris, 150 mM NaCl, 0.1% Tween 20, pH 7.5) and incubated overnight at 4 °C with the specific primary antibody followed by 1 h incubation with the respective secondary antibody at room temperature. Detection was carried out using Clarity Western ECL substrate (Bio-Rad) and a LAS 4000 automated detection system (GE Healthcare). Bands were quantified using ImageJ.

### Real-time qPCR

RNA was isolated by TRIzol–chloroform extraction. RNA was reverse-transcribed using a GoScript reverse transcription kit (Promega). qPCR was performed with a final amount of 20 ng of cDNA and EvaGreen Mix (Bio-Rad) with primer pairs for either mouse *Gapdh* (forward, GCTCATGACCACAGTCCAT; reverse, GTCATCATACTTGGCAGGTTT), mouse *Arl6ip1* (forward, GCTCTAATAAATGGACCACTG; reverse, GCACAAATGTCACAATCAGGT), human *GAPDH* (forward, GAAGGCTGGGGCTCATTT; reverse, GGACTGTGGTCATGAGTC) or human *ARL6IP1* (forward, GCTCCAATAAATGGACCACTGA; reverse, GGAAGTCACTATCAGGTAGGT) on a CFX96 Touch Real-Time PCR detection system (Bio-Rad).

### Immunofluorescence and autophagic flux analysis

For immunostainings, cells were fixed with 4% PFA at room temperature or ice-cold methanol, washed with PBS and permeabilized with 0.25% Triton X-100 in PBS at room temperature for 10  min, or with 0.1% saponin in PBS at room temperature for 1 min followed by blocking with 5% NGS in PBS for 1  h at room temperature. Incubation with primary antibodies diluted in 5% NGS and 0.25% Triton X-100 in PBS was carried out overnight at 4 °C or at room temperature for 1 h. After three consecutive washes with PBS, secondary antibodies were incubated for 1 h at room temperature. After three washes, cells were incubated at room temperature with Hoechst 33258 (Invitrogen) for 10 min. After a final wash with PBS, the coverslips were mounted with Fluoromount-G solution (ThermoFisher, 00-4958-02). Images were acquired using a confocal microscope (Zeiss LSM 880 with Airyscan).

For the analysis of ER-phagy, cells were transiently transfected with the mCherry–GFP–FAM134B reporter construct. Cells were fixed with ice-cold methanol for 10 min 24 h after transfection. Then cells were permeabilized with 0.25% Triton X-100 in PBS and blocked with 4% NGS for 1 h and stained for LC3B. Images were taken with a LSM 880 and analysed using the ComDet (v.0.5.5) plugin for ImageJ (https://github.com/ekatrukha/ComDet; settings: particle size = 10 pixels, co-localization distance = 7, intensity threshold = 20). The cell border was selected and the cell area determined. Only signals within the cell border were analysed. The intensity threshold was set at 1,000 for all channels, except for human fibroblasts, for which the threshold for mCherry was set at 200. Pearson’s coefficients were calculated using the JaCOP plugin in ImageJ and normalized to the ER area.

To assess whether ARL6IP1 is involved in bulk autophagy, we knocked down *ARL6IP1* in U2OS cells stably expressing the mCherry–GFP–LC3 reporter. Autophagy was triggered by 6 h of EBSS exposure or 6 h of 250 nM Torin1 exposure. Images were acquired with a high-content microscope–Yokogawa CQ1 confocal imaging cytometer. To assess whether ARL6IP1 is involved in mitophagy, ARL6IP1, CRISPR–Cas9 KO HeLa cells were transfected with the mitophagy reporter mCherry–GFP–FIS1. Autophagic flux was triggered with 40 μM CCCP for 4 h. Pexophagy was assessed after siRNA-mediated knockdown of *ARL6IP1* in U2OS cells after induction of the doxycycline-inducible reporter mCherry–GFP–PMP34 (ref. ^30^) at baseline or after starvation with EBSS for 20 h. Images were acquired with a confocal microscope (Zeiss LSM 880 with Airyscan). The red and yellow puncta were manually counted.

### Rescue experiment

*Arl6ip1* WT and KO MEFs were seeded in 24-well-plates at 40,000 cells per well. After 24 h, cells were transfected with the mCherry–GFP–FAM134B plasmid in combination with either the ARL6IP–HA or the ARL6IP1-7KR–HA plasmid. After 48 h, cells were fixed with ice-cold methanol for 10 min, permeabilized with 0.25% Triton X-100 in PBS and blocked with 4% NGS for 1 h and stained for LC3B and HA and further processed as described above. Images were taken with a LSM 880 and analysed using the ComDet (v.0.5.5) plugin for ImageJ (https://github.com/ekatrukha/ComDet; settings: particle size = 10 pixels, co-localization distance = 10, intensity threshold = 200/200/15). Only signals within the cell border were analysed.

### ER stress induction and cell viability count

MEFs or human fibroblasts were seeded in 6-well-plates and cultured to 70–80% confluency. Cells were washed with PBS and incubated with new medium with 1.5 µM thapsigargin (Sigma, T9033-5MG) or 5 µg ml^–1^ tunicamycin (Santa Cruz) without or in combination with 1 µM MG132 (Calbiochem, 474787-10MG) to inhibit the proteasome. After 24 h for MEFs and 48 h for human fibroblasts, the culture medium was removed, cells washed with PBS and trypsinized. All cells were pooled, centrifuged at 800 r.p.m. for 5 min (Heraeus Sepatech Megafuge 2.0R) and resuspended in fresh medium. Cell viability was measured by trypan blue exclusion with an automatic counting device (Bio-Rad TC20 automatic cell counter).

### Interactome analysis and sample preparation for MS

For the LC–MS interactome analysis, HA–FAM134B expression was induced in U2OS cells with doxycycline. HEK293T cells were transiently transfected with the constructs V1-ARL6IP1, V2-ARL6IP1, V1-FAM134B and V2-FAM134B. For in vivo ubiquitination, HEK293T cells were transiently transfected with the plasmids V1-ARL6IP1, V2-ARL6IP1, AMFR-V2 and AMFR-V2-C356G-H361A. After 24 h, cells were lysed with 1% Triton X-100 IP buffer (50 mM Tris-HCL, 150 mM NaCl and 0.5 mM EDTA). Lysis buffer without detergents was added to protein lysates to dilute Triton X-100 to 0.3%. Then samples were incubated with HA-agarose beads (Sigma-Aldrich, A2095) or GFP-Trap beads (Chromotek, gta-20) overnight at 4 °C on a rotating platform. Protein-bound beads were washed three times with lysis buffer supplemented with 0.1% Triton X-100 and once with lysis buffer without detergents. HA IP samples were incubated with 40 µl denaturing buffer (2% sodium deoxycholate, 1 mM tris(2-carboxyethyl)phosphine, 4 mM chloroacetamide and 50 mM Tris-HCl pH 8.5) and heated at 95 °C for 10 min. Samples were mixed 1:1 with 500 ng LysC (Promega), incubated for 3 h at 37 °C and digested with 500 ng of trypsin in 50 mM Tris-HCl, pH 8.5, overnight at 37 °C. GFP IP samples were denatured with 25 µl denaturing buffer at 60 °C for 30 min. After cooling down, the samples were digested with 25 µl 50 mM Tris with 1 µl of trypsin (500 ng) at 37 °C overnight. Reactions were stopped by addition of 150 µl of isopropanol containing 1% trifluoroacetic acid (TFA). Peptides were cleaned up by loading them onto SDB-RPS stage tips (Sigma). After one wash with 1% TFA in isopropanol and one wash with 0.2% TFA in water, peptides were eluted using 80% acetonitrile and 1.25% ammonia. Eluted peptides were dried, tandem mass tagged labelled and processed for LC–MS measurements.

### LC–MS analysis

Dried peptides were reconstituted in 2% acetonitrile, 0.1% TFA and analysed on a Q Exactive HF mass spectrometer coupled to an easy nLC 1200 (ThermoFisher Scientific) using a 35-cm-long, 75 µm inner diameter fused-silica column packed in-house with 1.9 µm C18 particles (Reprosil pur, Dr. Maisch) and kept at 50 °C using an integrated column oven (Sonation). Peptides were eluted using a nonlinear gradient from 4 to 28% acetonitrile over 45 min and directly sprayed into the mass spectrometer equipped with a nanoFlex ion source (ThermoFisher Scientific). Full scan MS spectra (300–1,650 *m/z*) were acquired in profile mode at a resolution of 60,000 at *m/z* 200, a maximum injection time of 20 ms and an automatic gain control target value of 3 × 10^6^ charges. Up to 15 most intense peptides per full scan were isolated using a 1.4 Th window and fragmented using higher energy collisional dissociation (normalized collision energy of 28). MS/MS spectra were acquired in centroid mode with a resolution of 30,000, a maximum injection time of 45 ms and an automatic gain control target value of 1 × 10^5^. Single charged ions, ions with a charge state above 4 and ions with unassigned charge states were not considered for fragmentation, and dynamic exclusion was set to 20 s to minimize the acquisition of fragment spectra of already acquired precursors.

### Proteomics data processing

MS raw data were processed using MaxQuant (v.1.6.10.43) applying default parameters. Acquired spectra were searched against the human ‘one sequence per gene’ database (taxonomy identifier 9606) downloaded from UniProt (accessed 3 March 2020; 20,531 sequences) and a collection of 244 common contaminants (“contaminants.fasta” provided with MaxQuant) using the Andromeda search engine integrated in MaxQuant^31,32^. Identifications were filtered to obtain false discovery rates (FDRs) below 1% for both peptide spectrum matches (minimum length of 7 amino acids) and proteins using a target–decoy strategy^33^. Protein quantification and data normalization relied on the MaxLFQ algorithm implemented in MaxQuant^34^. The MS proteomics data have been deposited to the ProteomeXchange Consortium^35^ through the PRIDE partner repository^36^ with the dataset identifiers PXD032718, PXD032720 and PXD039184. All acquired raw files were processed using MaxQuant (v.1.6.10.43) and the implemented Andromeda search engine. For protein assignment, spectra were correlated with the UniProt human database (v.2019) including a list of common contaminants. Searches were performed with tryptic specifications and default settings for mass tolerances for MS and MS/MS spectra. Carbamidomethyl at cysteine residues, oxidations at methionine, acetylation at the N terminus were defined as a fixed modification. The minimal peptide length was set to 7 amino acids and the FDR for proteins and peptide-spectrum matches to 1%. The match-between-run feature with a time window of 1 min was used. For further analysis, Perseus software (v.1.6.6.0) was used and first filtered for contaminants and reverse entries as well as proteins that were only identified by a modified peptide.

### Proximity ligation assays

Proximity ligation assays (PLAs) were performed using a Duolink in situ red starter kit mouse/rabbit (DUO92101, Sigma-Aldrich) according to the manufacturer’s instructions with rabbit anti-FAM134B (ref. ^1^) and rabbit anti-ARL6IP1 (PRS3305, Sigma-Aldrich) antibodies using the Minus (DUO92010) and the Plus probe (DUO92009). Rabbit anti-FAM134B-Plus was used in a 1:5 dilution and rabbit anti-ARL6IP1-Minus in a 1:10 dilution on WT, *Arl6ip1* and *Fam134b* KO MEFs after PFA (4%) fixation and permeabilization with 0.25% (v/v) Triton X-100 in PBS.

### Fluorescence protease protection assay

We followed a fluorescence protease protection assay protocol as previously described^37^. In brief, 75,000 COS-7 cells per well were seeded on 18 mm coverslips coated with 0.1 mg ml^–1^ PLL. The next day, cells were transfected with the respective constructs using Lipofectamine 2000. After two more days, cells were washed with pre-warmed intracellular buffer (50 mM HEPES, pH 7.5, 23  mM NaCl, 3 mM MgCl_2_, 100 nM CaCl_2_, 1 mM EGTA and freshly added 107 mM potassium glutamate, 1 mM ATP and 2  mM dithiothreitol) and transferred to a heated perfusion chamber filled with the same buffer. Live cell imaging for both GFP and RFP was initiated on a Zeiss Cell Observer Z1 with a frame every 20 s starting with a pre-permeabilization image followed by manual administration of 18 µM digitonin. After 120 s, the buffer was replaced by intracellular buffer containing 6 mM freshly added trypsin. Analysis was carried out using ImageJ by drawing the outline of the selected cell and measuring the mean fluorescence intensity of the surrounded area subtracted by the background intensity taken from a cell free spot of the same frame. For further analysis, the area under the curve was calculated between 160 and 720 s.

### Purification of recombinant proteins

Trx–His fusion proteins were purified from *Escherichia coli* treated with 0.5 mM IPTG (overnight, 18 °C). Eluted proteins were concentrated using Amicon Ultra-4-10k centrifugal filter units (Millipore) and then either dialysed at 4 °C against HN buffer (20 mM HEPES/KOH pH 7.4, 150 mM NaCl and 2.5 mM dithiothreitol) (material used for Fig. 4 and Extended Data Fig. 5 analyses) or against liposome buffer (25 mM HEPES-KOH, pH 7.2, 25 mM KCl, 2.5 mM magnesium acetate and 100 mM potassium glutamate) (protein used for studies presented in Fig. 2).

### Liposome preparation, liposome incubations, freeze-fracturing and TEM

Liposomes were prepared using Folch-fraction type I lipids (Sigma-Aldrich) according to previously described procedures^10,38^. Liposome co-floatation assays were performed as previously reported^10^. In brief, liposomes and purified recombinant protein were incubated for 15 min at 37 °C in 0.3 M sucrose in liposome buffer, mixed with 75% sucrose in liposome buffer, overlaid with 200  μl 35% sucrose and 200 μl liposome buffer and then centrifuged at 200,000*g* for 30 min at 28 °C. Six fractions were collected from top to bottom and analysed by SDS–PAGE and fluorescence-based western blotting using a LICOR Odyssey system (LICOR Bioscience).

For shaping assays presented in Fig. 2, 1 mg of liposomes was incubated with 5 μM protein in liposome buffer containing 0.3 M sucrose for 15 min at 37 °C. For shaping assays presented in Fig. 4 and Extended Data Fig. 5, 1 mg of liposomes was incubated at 37 °C with 2.5 μM protein in HN buffer containing 0.3 M sucrose for 15 min. Thereafter, 20 μg proteinase K was added to avoid liposomal aggregates. The reaction was performed for 40 min at 45 °C (ref. ^39^). Small aliquots of the liposome suspension were then used for freeze-fracturing. The grids with the samples were systematically explored using an EM 900 electron microscope (Zeiss) operated at 80 kV. Images were acquired with a Wide-angle Dual Speed 2K (Tröndle) CCD camera. The diameters of liposomes were determined using ImageJ.

### In vitro ubiquitination assay of ARL6IP1 with recombinant AMFR and sample preparation for MS

For the ubiquitination assay with AMFR, 1 µM purified Trx-His–ARL6IP1, 10 µM ubiquitin (in-house), 10 mM ATP and 10 mM MgCl_2_ were incubated with 0.8 µM AMFR (provided by B. Schulman, Max Planck Institute of Biochemistry), 100 nM E1 UBA1 (in-house) and 0.8 µM E2 UBE2G2 (Biotechne) in 150 mM NaCl, 50 mM Tris-HCl, pH 7.5, at 37 °C for 2 h. The reaction mixture was analysed by SDS–PAGE and Coomassie staining or immunoblot analysis for GST (Cell Signaling Technology), His (Cell Signaling Technology) and ubiquitin (Cell Signaling Technology) or by mapping of ubiquitinated lysines by MS.

### Ubiquitination assays in cells, co-immunoprecipitations and TUBE2 pull-down

The ubiquitination of ARL6IP1 was assessed in HEK293T cells transfected with Myc–ubiquitin, HA–ARL6IP1 and either AMFR–Flag or its catalytically inactive RING mutant. Cells were lysed in lysis buffer (50 mM Tris-HCl, pH 7.5, 150 mM NaCl, 0.5 mM EDTA, 1% Triton X-100, 10 mM *N*-ethylmaleimide and protease inhibitors (Roche Diagnostics, 5892791001)). The lysates were then incubated on ice for 15 min and centrifuged at 12,000*g* at 4 °C for 30 min. Next, 40 µl of the supernatant was collected, mixed with Laemmli sample buffer, boiled for 5 min at 95 °C and stored at –20 °C as input control. Myc-tagged ubiquitinated proteins were immunoprecipitated from lysates cleared with Myc-Trap Agarose (Chromotek, yta-10). Beads were washed three times with lysis buffer, heated at 95 °C for 5 min, subjected to SDS–PAGE and analysed by immunoblotting to detect the HA-Tag. For other immunoprecipitation assays, cleared lysates were incubated with GFP-Trap (Chromotek, gta-20), HA-agarose beads (Sigma-Aldrich, A2095) or TUBE2 agarose beads (Life Sensors, UM402) and incubated at 4 °C overnight. The next day, tubes were centrifuged (800*g*, 4 °C, 5 min), the supernatants removed and the beads washed with ice-cold lysis buffer. Input and co-precipitated fractions were analysed by SDS–PAGE and immunoblotting. For co-immunoprecipitation of endogenous ARL6IP1 and FAM134B, a confluent 15 cm dish of *Arl6ip1* KO or WT MEFs was collected. Lysates were cleared by centrifugation at 12,000*g* for 10 min and incubated with the ARL6IP1 primary antibody at 4 °C overnight. Protein A agarose beads (Roche, 11719408001) were added and incubated at 4 °C for 4 h. Beads were then washed three times with lysis buffer, re-suspended in Laemmli buffer and boiled. Supernatants were analysed by SDS–PAGE and immunoblotting.

### Modelling and simulations of ARL6IP1

The atomic model of human ARL6IP1 was built using the AI-based AlphaFold (v.2) program^40^. Five models were constructed, and the top-ranked model was chosen as it had maximal overlap with predicted secondary structures and consensus transmembrane topology, a higher pLDDT score and a relatively lower predicted alignment error (AF confidence measure).

Coarse-grained (CG) molecular dynamics simulations were performed using the MARTINI model (v.2.2)^41,42^. CG models of ubiquitinated and non-ubiquitinated versions were built by using martinize.py^43^. DSSP assignments were used to generate backbone restraints to preserve local secondary structure^44,45^. In the ubiquitinated protein (ARL6IP1-K96-Ub), the iso-peptide bond between K96 and the terminal glycine (G76) of ubiquitin (Protein Data Bank identifier 1UBQ) was modelled by modifying the side chain lysine bead (SC2/+1) into a neutral backbone bead (BB/0) and restraining the distance between the terminal bead of ubiquitin and the lysine side chain to 0.35 nm with a force constant of *k* = 1,250 kJ (mol nm^2^)^–1^. CG protein models were embedded into POPC (16:0-18:1 PC) bilayers spanning the *xy* plane of a periodic simulation box (20 × 20 × 20 nm^3^) solvated with CG-water containing 150 mM NaCl using the insane.py script^43^. All systems were first energy minimized and then equilibrated using the Berendsen thermostat^46^ and barostat^47^ along with position restraints on protein backbone beads followed by production runs with a 20 fs time step for a total of 10 μs. The system temperature and pressure were maintained at 310 K and 1 atm with the velocity rescaling thermostat^48^ and the semi-isotropic Parrinello–Rahman barostat^49^, respectively. All simulations were performed using gromacs (v.2019.3)^50,51^.

### Statistical analysis

All experiments were performed in at least three independent biological replicates unless indicated otherwise. Data are presented as the mean ± s.e.m. unless indicated otherwise. Data analyses were performed using GraphPad Prism 9. For statistical analysis, raw data were analysed for normal distribution with the Kolmogorov–Smirnov test or with graphical analysis using Q-Q-plot. If appropriate, we used one-way analysis of variance (with Bonferroni post-hoc test unless indicated otherwise), repeated-measures two-way analysis of variance, Kruskal–Wallis *H*-test, Student’s *t*-test (two-sided unless indicated otherwise) or Mann–Whitney *U*-test. *P* values less than 0.05 were considered significant.

### Reporting summary

Further information on research design is available in the Nature Portfolio Reporting Summary linked to this article.

## Online content

Any methods, additional references, Nature Portfolio reporting summaries, source data, extended data, supplementary information, acknowledgements, peer review information; details of author contributions and competing interests; and statements of data and code availability are available at 10.1038/s41586-023-06090-9.

## Supplementary information

Supplementary Information Western blots.

Reporting Summary

Supplementary Table 1 Plasmids used in the study.

Supplementary Table 2 Primers used for this study.

Supplementary Table 3 Primary antibodies and their application related to this study.

Supplementary Table 4 Secondary antibodies and their application related to this study.

Supplementary Table 5 sgRNA sequences used to generate the *ARL6IP1* KO cell lines.

## Data Availability

The MS spectrometry proteomics data have been deposited to the ProteomeXchange Consortium^35^ through the PRIDE partner repository^36^ with the dataset identifiers PXD032718, PXD032720 and PXD039184. All source data in main and extended data figures are provided as supplementary information. This also includes gels and blots. Materials and associated protocols are available upon request without undue qualifications. Source data are provided with this paper.

## Source data

Source Data Fig. 1
Source Data Fig. 2
Source Data Fig. 4
Source Data Fig. 5
Source Data Extended Data Fig. 1
Source Data Extended Data Fig. 5
Source Data Extended Data Fig. 6
Source Data Extended Data Fig. 7
Source Data Extended Data Fig. 8
Source Data Extended Data Fig. 9
Source Data Extended Data Fig. 10
