# Supplementary material for: Heteromeric clusters of ubiquitinated ER-shaping proteins drive ER-phagy
Source: Nature. 2023 May 24;618(7964):402–10. doi: 10.1038/s41586-023-06090-9 (PMC10247384; doi:10.1038/s41586-023-06090-9)
Supplement: Supplementary file 1 — Western blots. [file 41586_2023_6090_MOESM1_ESM.pdf]

nature portfolio

https://doi.org/10.1038/s41586-023-06090-9

Supplementary information

Heteromeric clusters of ubiquitinated ER-shaping proteins drive ER-phagy

In the format provided by the authors and unedited

FIGURE 1 - C

n1

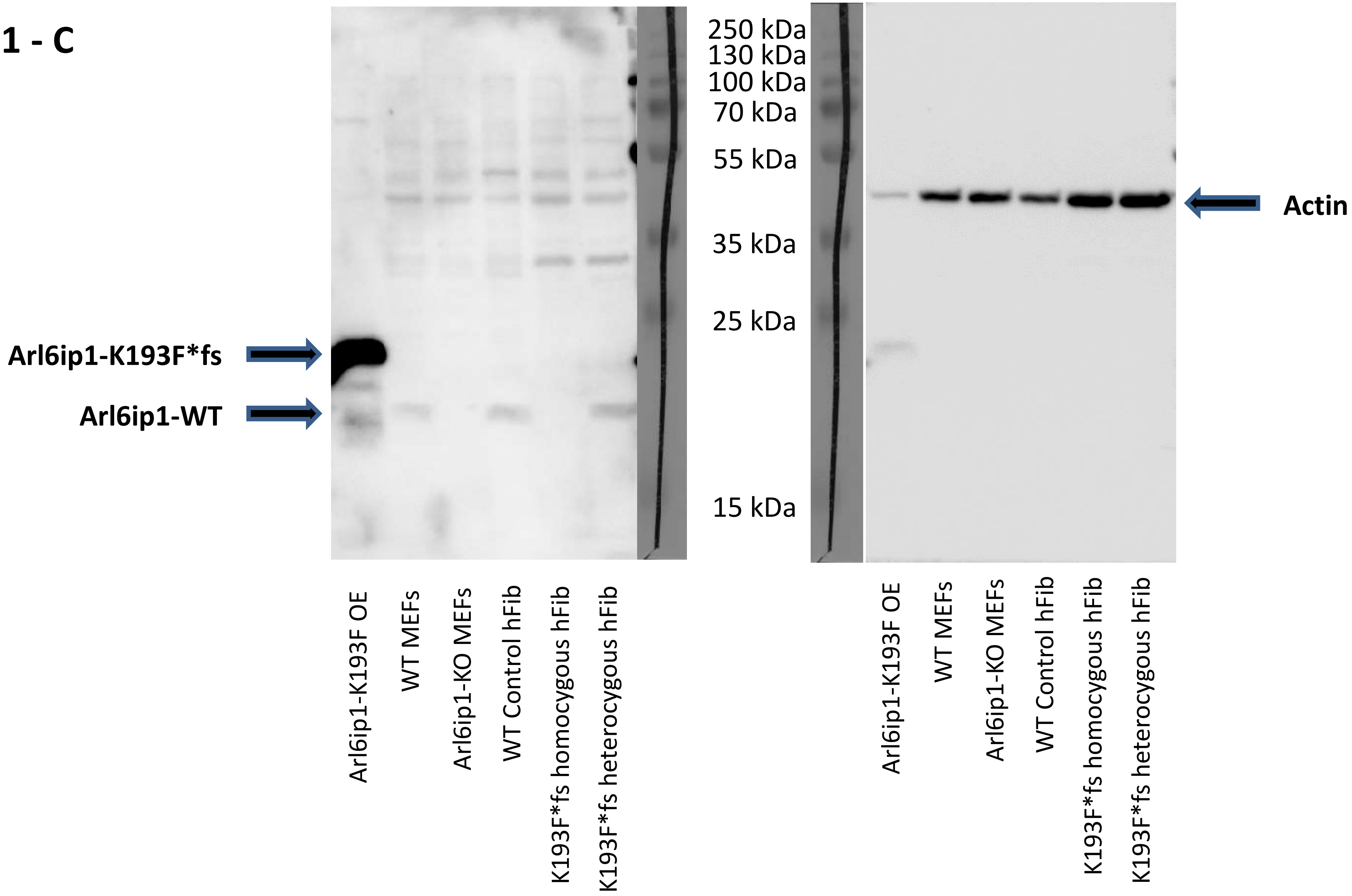

Arl6ip1-K193F*fs
Arl6ip1-WT
250 kDa
130 kDa
100 kDa
70 kDa
55 kDa
35 kDa
25 kDa
15 kDa
Actin
Arl6ip1-K193F OE
WT MEFs
Arl6ip1-KO MEFs
WT Control hFib
K193F*fs homocygous hFib
K193F*fs heterocygous hFib
Arl6ip1-K193F OE
WT MEFs
Arl6ip1-KO MEFs
WT Control hFib
K193F*fs homocygous hFib
K193F*fs heterocygous hFib

FIGURE 1 - C

n2

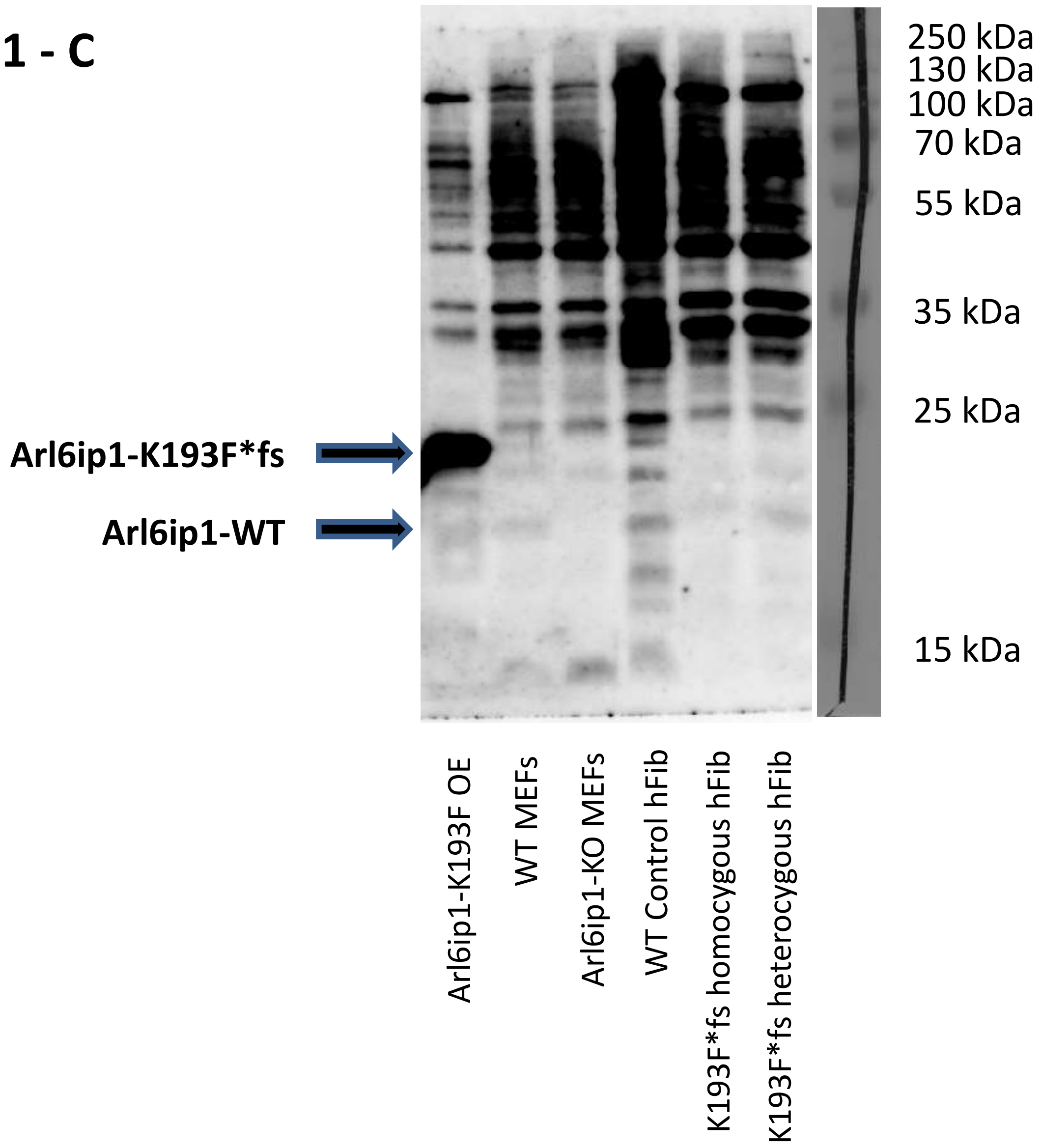
250 kDa
130 kDa
100 kDa
70 kDa
55 kDa
35 kDa
25 kDa
15 kDa
Arl6ip1-K193F*fs
Arl6ip1-WT
Arl6ip1-K193F OE
WT MEFs
Arl6ip1-KO MEFs
WT Control hFib
K193F*fs homocygous hFib
K193F*fs heterocygous hFib

FIGURE 1 - F

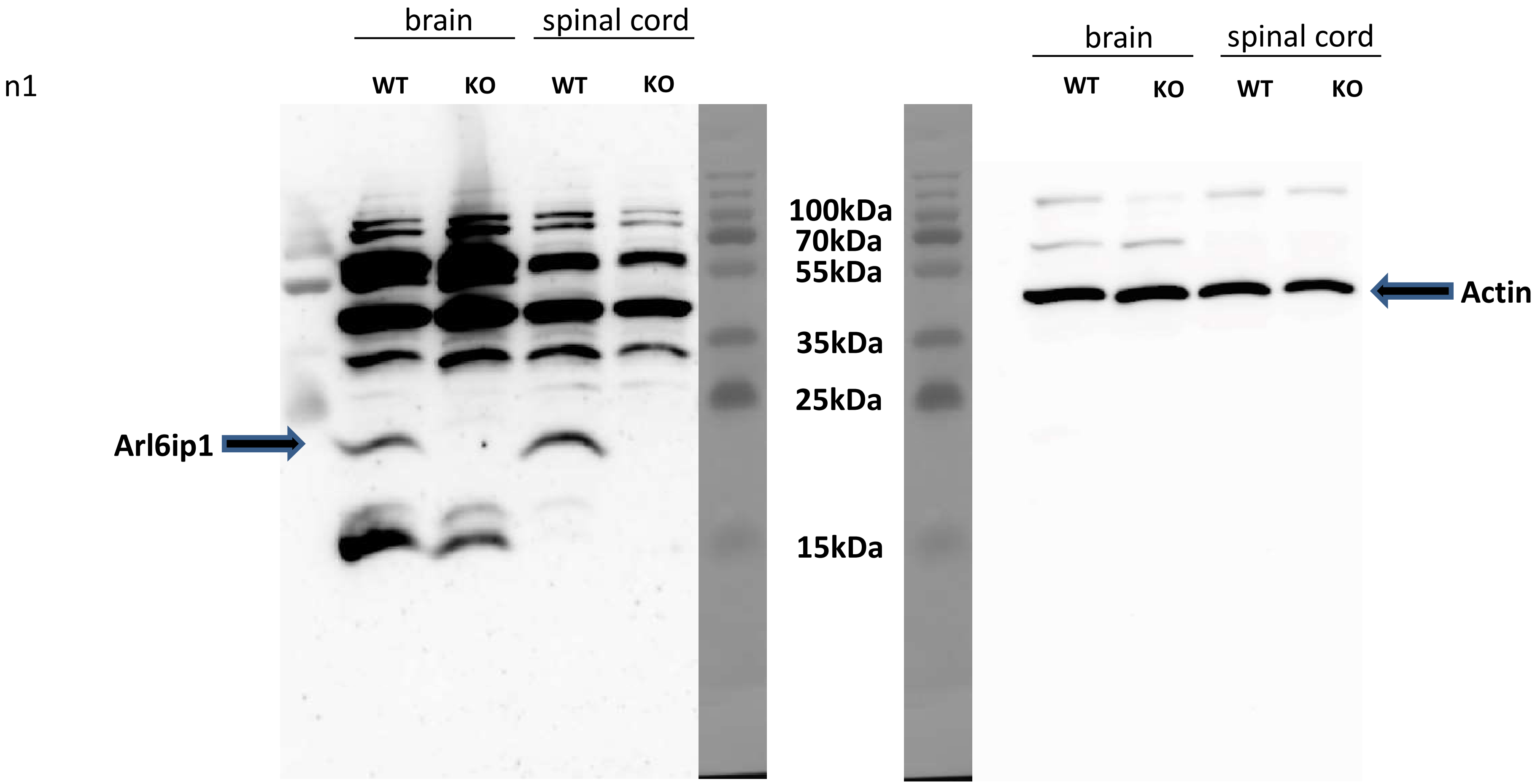

n1
brain
spinal cord
WT
KO
WT
KO
Arl6ip1
100kDa
70kDa
55kDa
35kDa
25kDa
15kDa
brain
spinal cord
WT
KO
WT
KO
Actin

FIGURE 1 - F

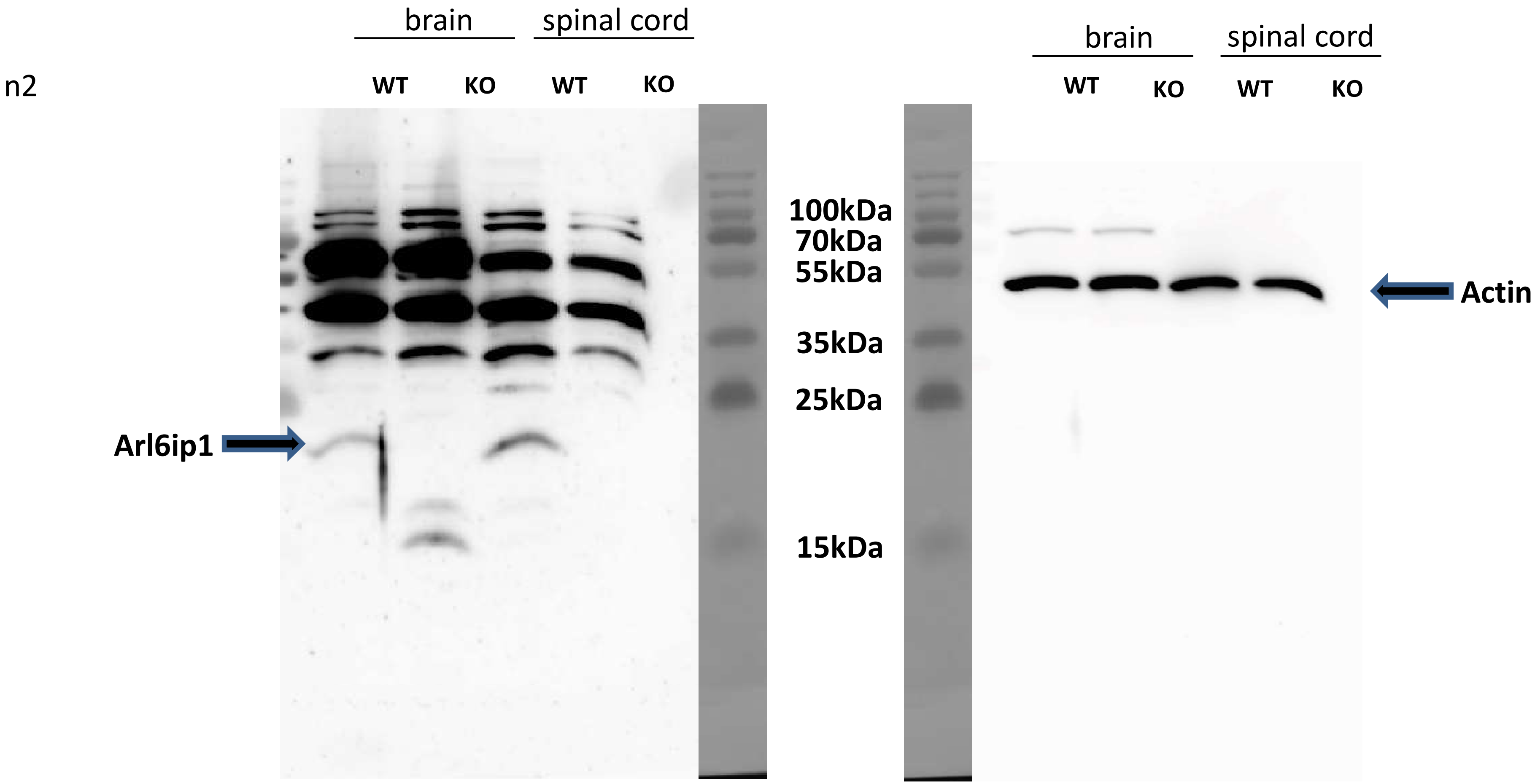

n2
brain
spinal cord
WT
KO
WT
KO
100kDa
70kDa
55kDa
35kDa
25kDa
15kDa
Arl6ip1
brain
spinal cord
WT
KO
WT
KO
Actin

FIG 2. C

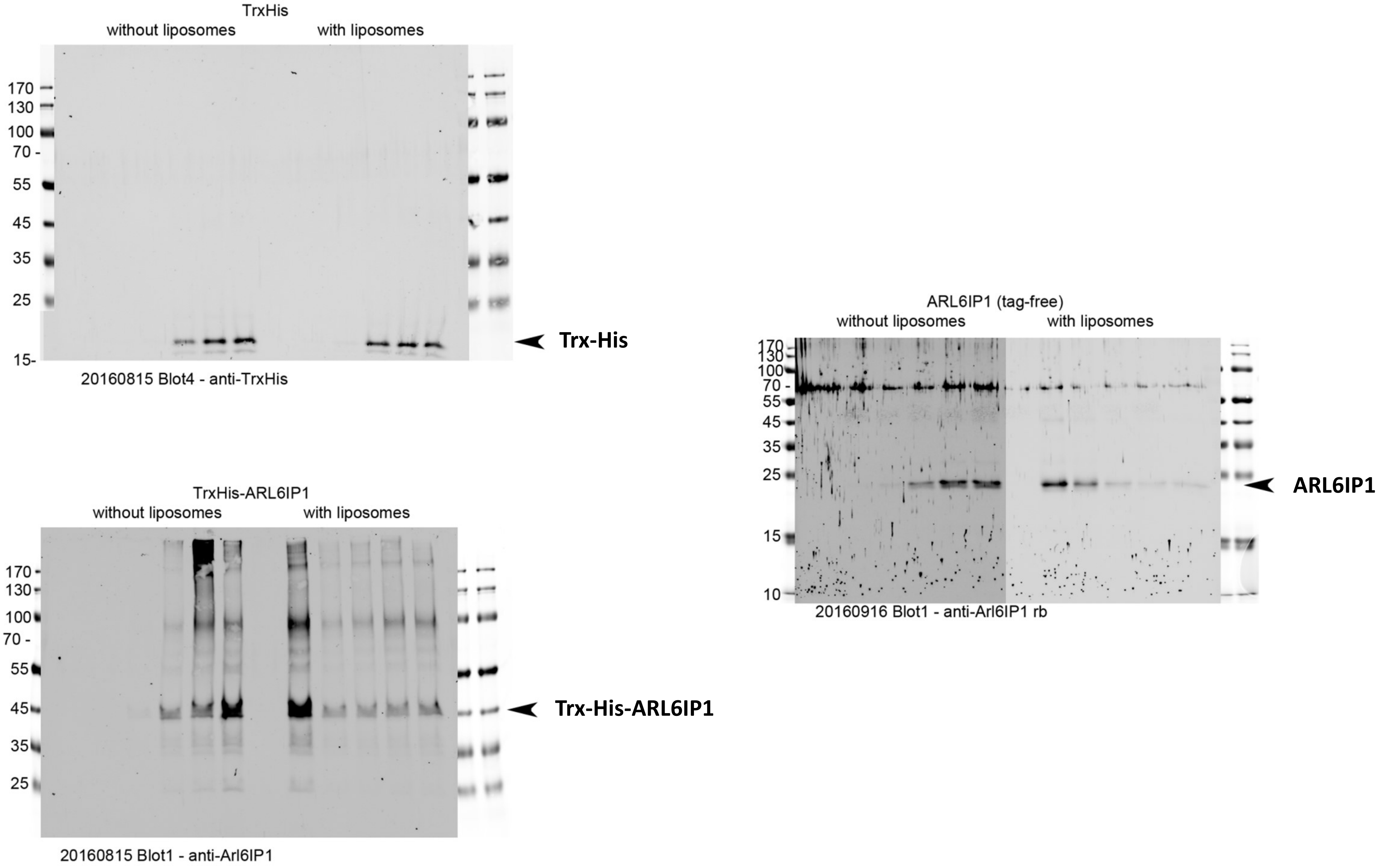

TrxHis
without liposomes
with liposomes
170
130
100
70 -
55
45
35
25
15-
Trx-His
20160815 Blot4 - anti-TrxHis
TrxHis-ARL6IP1
without liposomes
with liposomes
170
130
100
70 -
55
45
35
25
Trx-His-ARL6IP1
20160815 Blot1 - anti-Arl6IP1
ARL6IP1 (tag-free)
without liposomes
with liposomes
170
130
100
70
55
45
35
25
15
10
ARL6IP1
20160916 Blot1 - anti-Arl6IP1 rb

FIG 2. E

INPUT

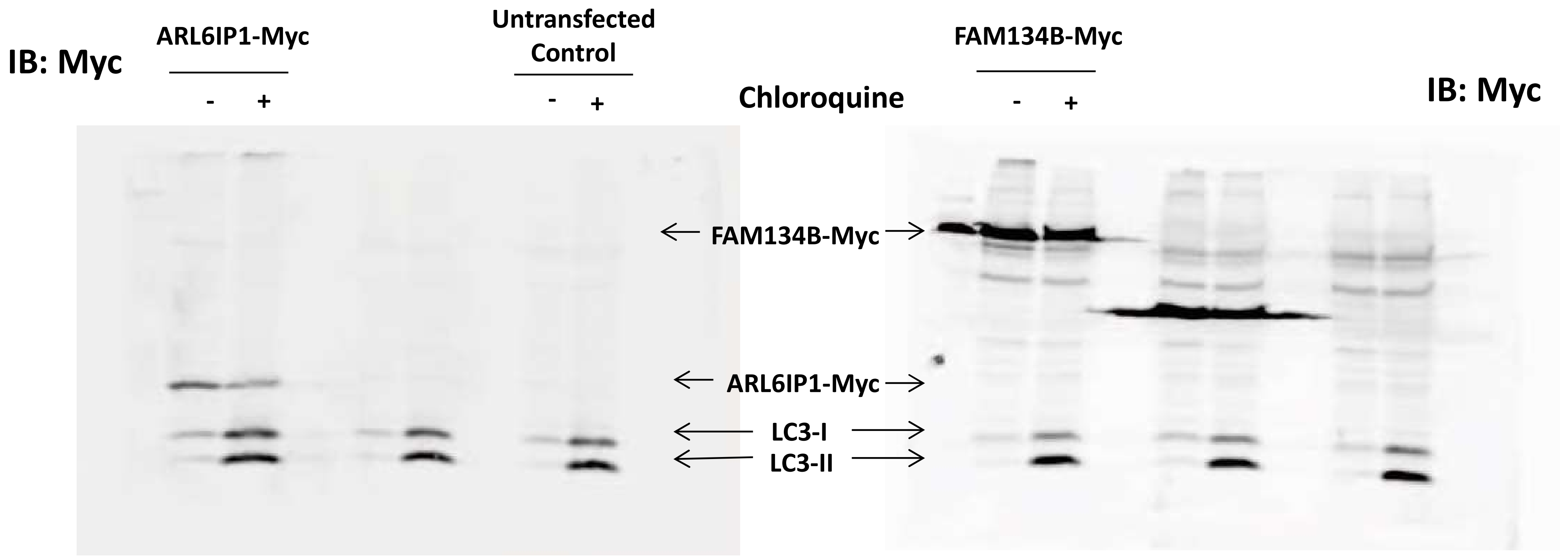
IB: Myc
ARL6IP1-Myc
Untransfected Control
FAM134B-Myc
- +
- +
Chloroquine
- +
IB: Myc
FAM134B-Myc
ARL6IP1-Myc
LC3-I
LC3-II

FIG 2. E

IP Myc

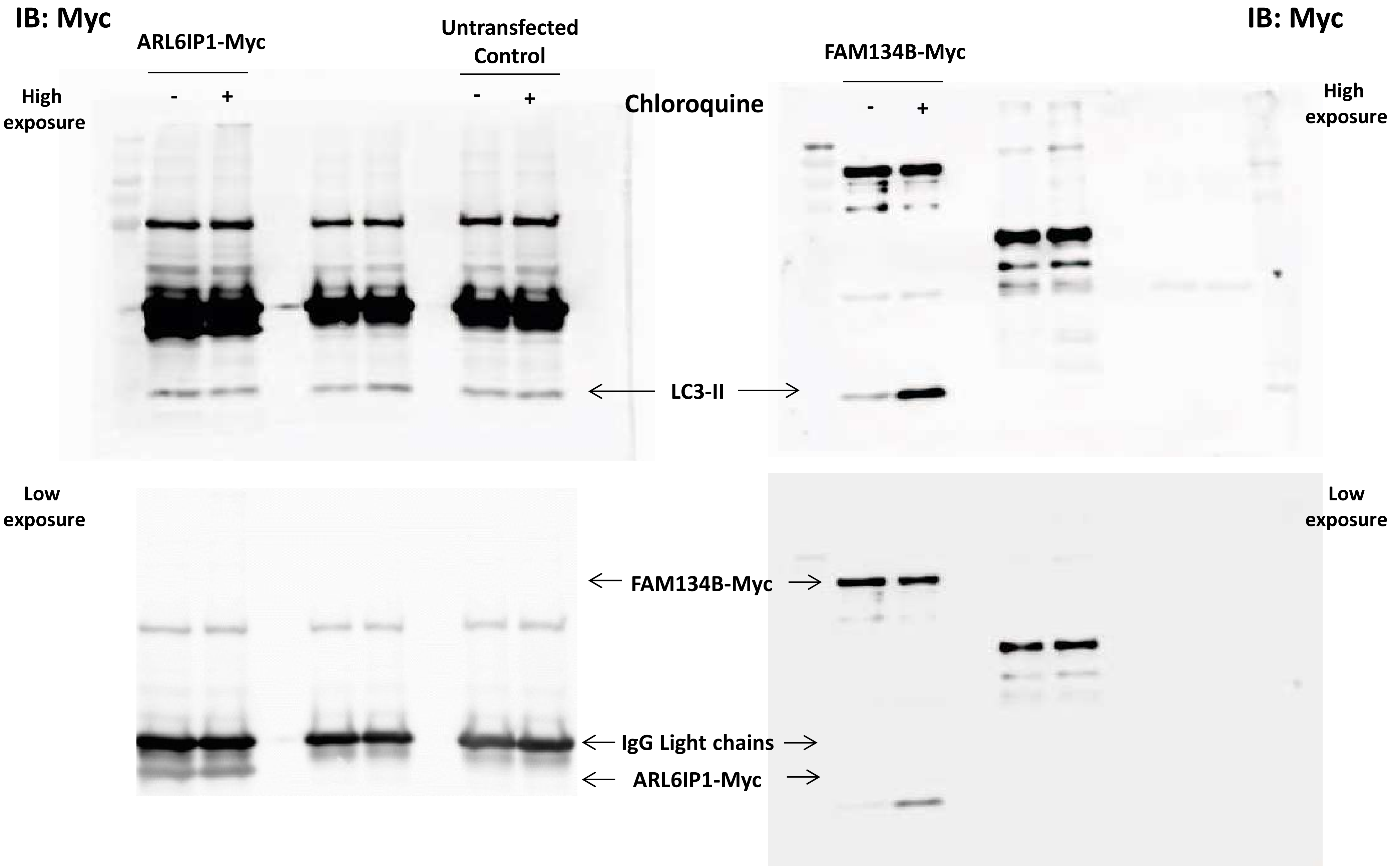

IB: Myc
ARL6IP1-Myc
Untransfected Control
High exposure
- +
- +
Chloroquine
FAM134B-Myc
- +
IB: Myc
High exposure
LC3-II
Low exposure
FAM134B-Myc
IgG Light chains
ARL6IP1-Myc
Low exposure

FIG 2. G

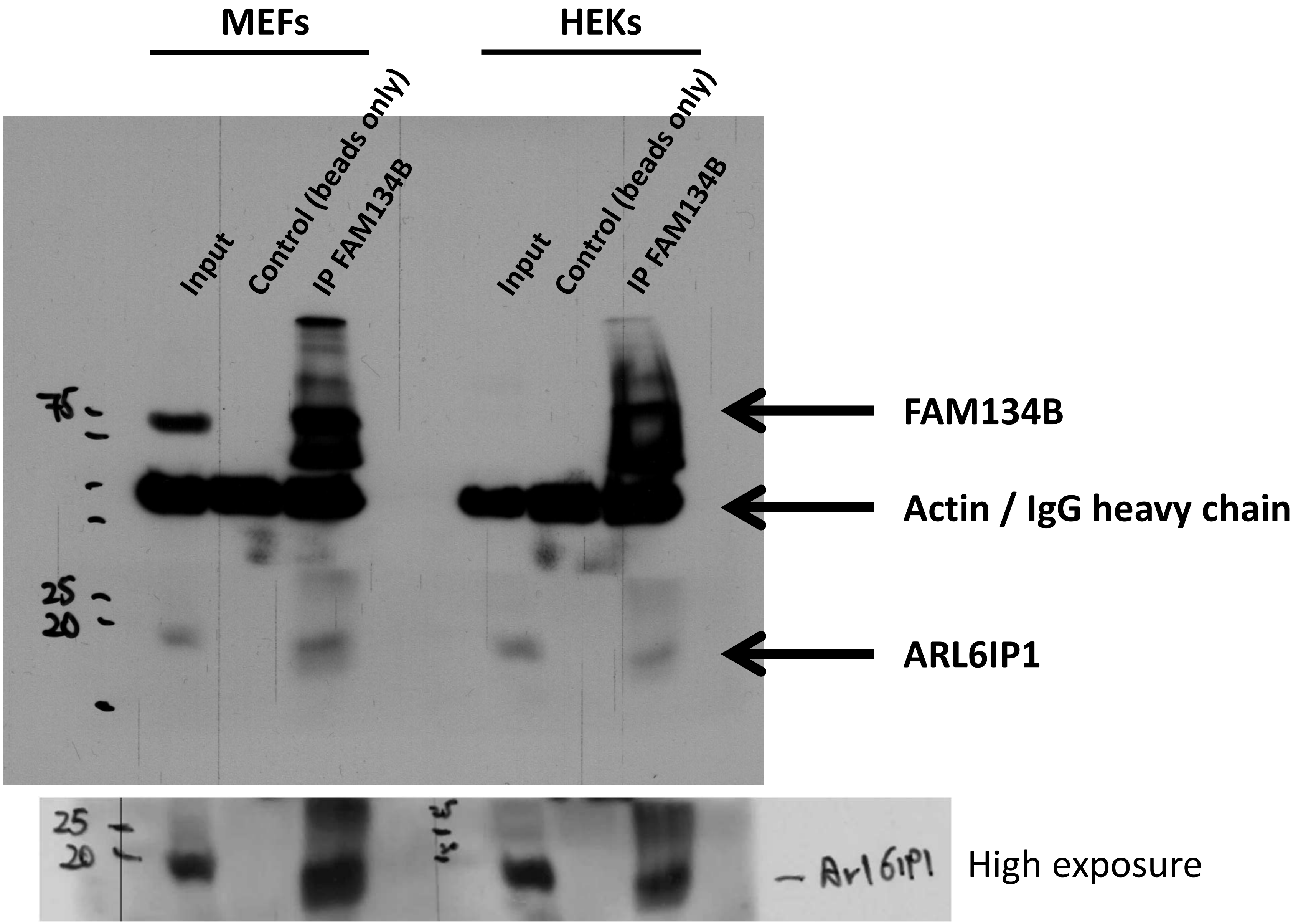

MEFs
HEKs
Input
Control (beads only)
IP FAM134B
Input
Control (beads only)
IP FAM134B
75
25
20
FAM134B
Actin / IgG heavy chain
ARL6IP1
25
20
Arl6IP1
High exposure

FIG 4. C

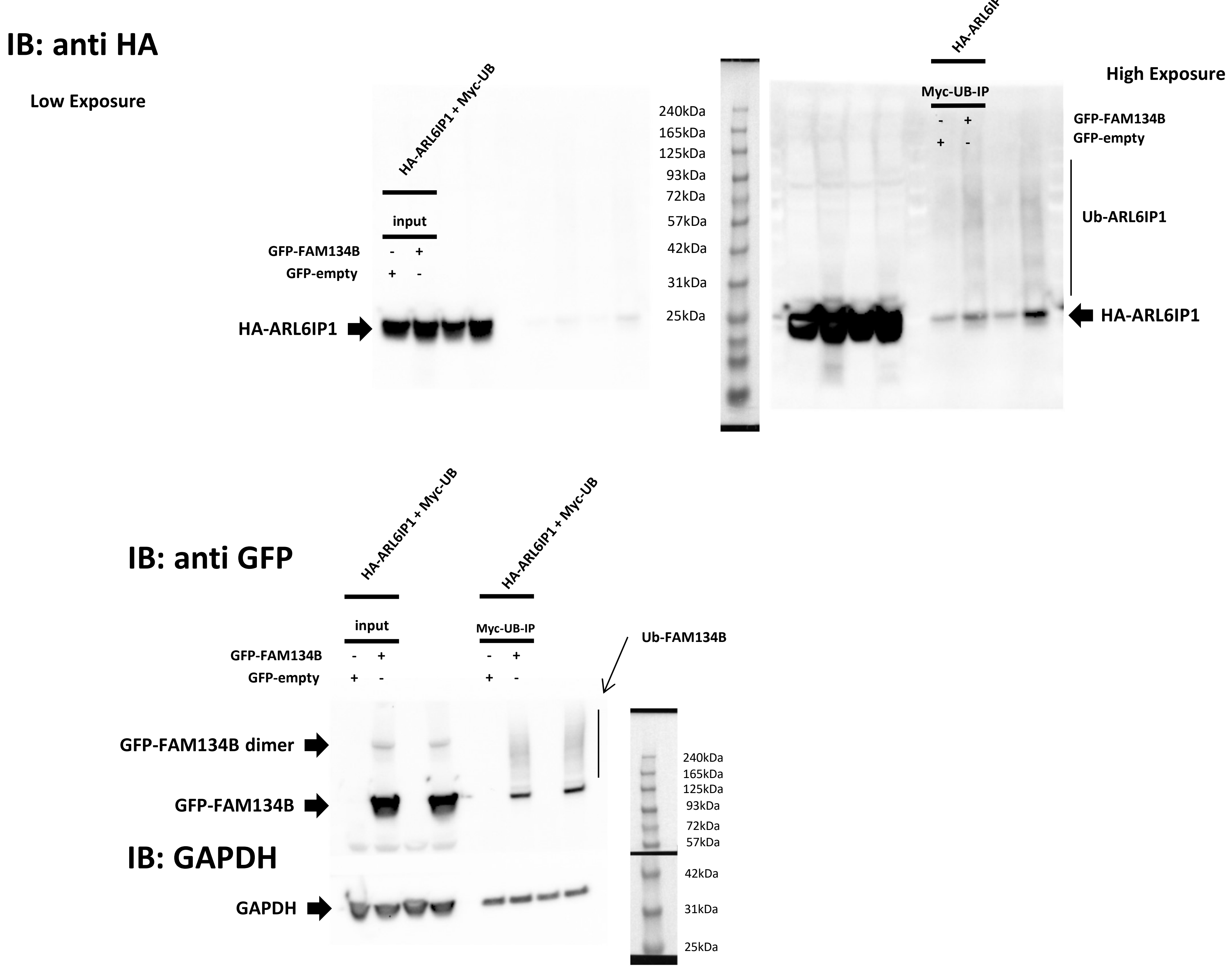
IB: anti HA
Low Exposure
HA-ARL6IP1 + Myc-UB
input
GFP-FAM134B - +
GFP-empty + -
HA-ARL6IP1
240kDa
165kDa
125kDa
93kDa
72kDa
57kDa
42kDa
31kDa
25kDa
HA-ARL6IP1 + Myc-UB
Myc-UB-IP
- +
+ -
High Exposure
GFP-FAM134B
GFP-empty
Ub-ARL6IP1
HA-ARL6IP1
IB: anti GFP
HA-ARL6IP1 + Myc-UB
HA-ARL6IP1 + Myc-UB
input
Myc-UB-IP
GFP-FAM134B - + - +
GFP-empty + - + -
Ub-FAM134B
GFP-FAM134B dimer
GFP-FAM134B
IB: GAPDH
GAPDH
240kDa
165kDa
125kDa
93kDa
72kDa
57kDa
42kDa
31kDa
25kDa

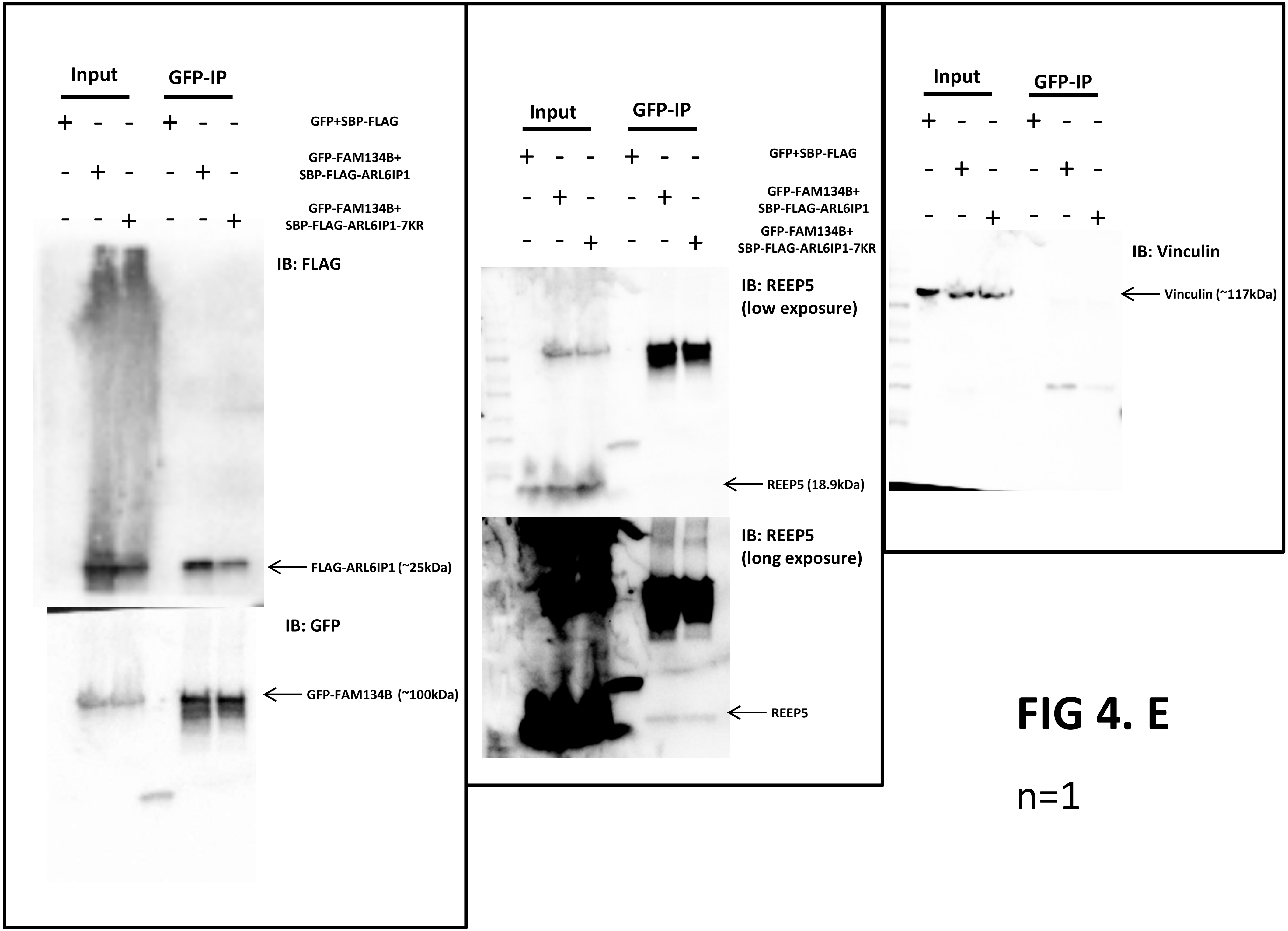
Input
GFP-IP
GFP+SBP-FLAG
GFP-FAM134B+
SBP-FLAG-ARL6IP1
GFP-FAM134B+
SBP-FLAG-ARL6IP1-7KR
IB: FLAG
FLAG-ARL6IP1 (~25kDa)
IB: GFP
GFP-FAM134B (~100kDa)
IB: REEP5
(low exposure)
REEP5 (18.9kDa)
IB: REEP5
(long exposure)
REEP5
IB: Vinculin
Vinculin (~117kDa)

FIG 4. E

n=1

FIG 4. E

n=2

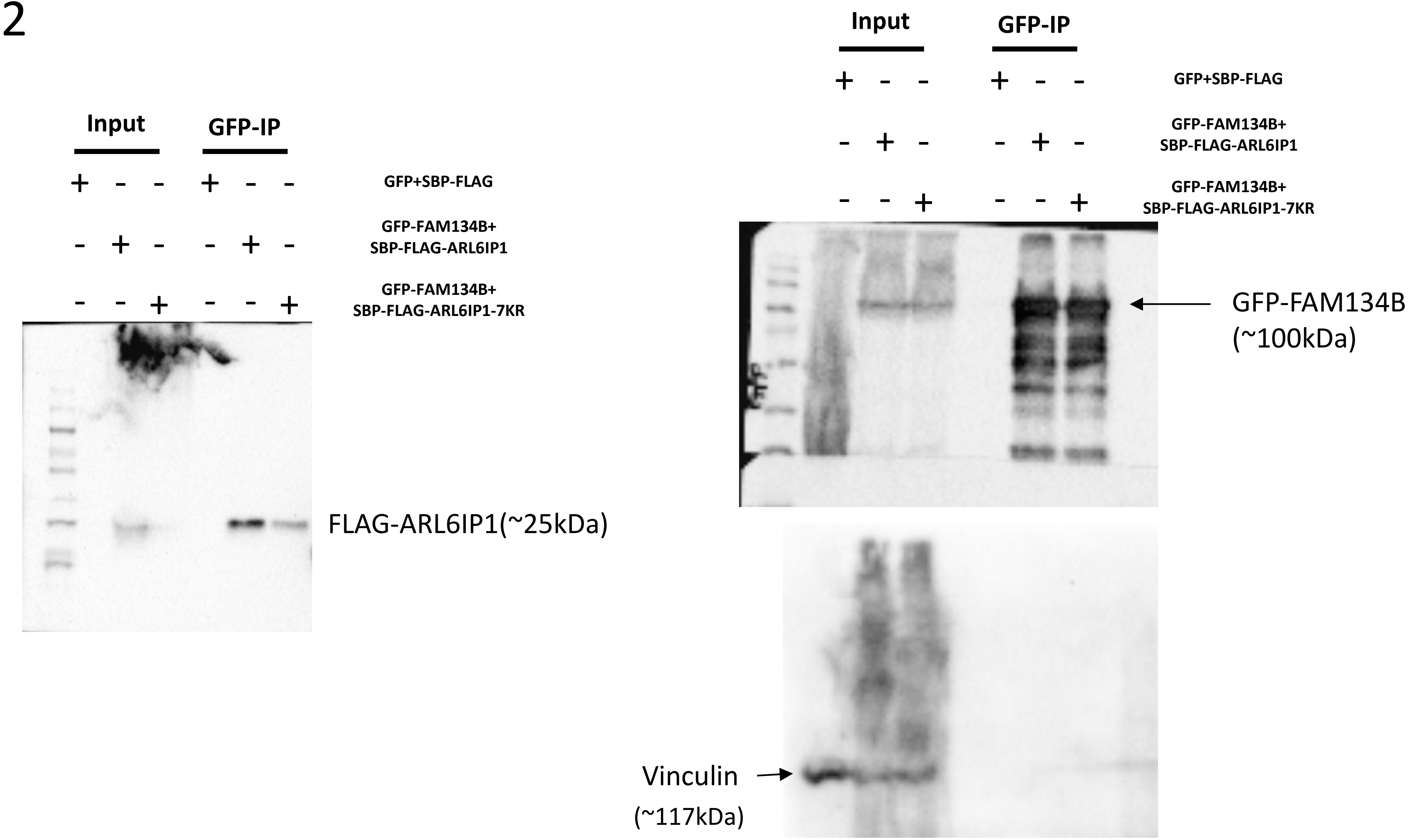
Input
GFP-IP
GFP+SBP-FLAG
GFP-FAM134B+
SBP-FLAG-ARL6IP1
GFP-FAM134B+
SBP-FLAG-ARL6IP1-7KR
FLAG-ARL6IP1(~25kDa)
Input
GFP-IP
GFP+SBP-FLAG
GFP-FAM134B+
SBP-FLAG-ARL6IP1
GFP-FAM134B+
SBP-FLAG-ARL6IP1-7KR
GFP-FAM134B
(~100kDa)
Vinculin
(~117kDa)

FIG 4. G

ARL6IP1
(23kDa)

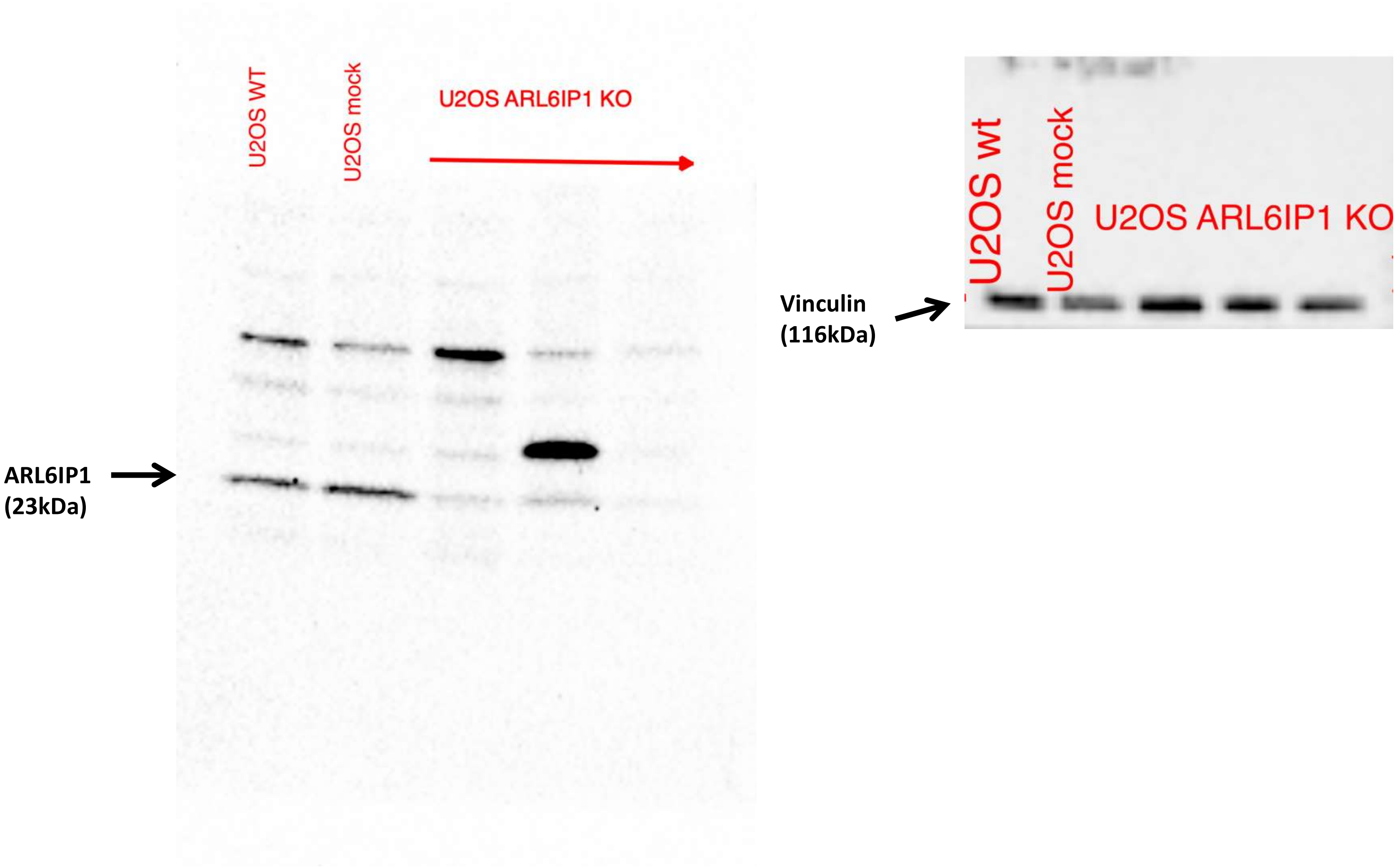

FIG 4. H

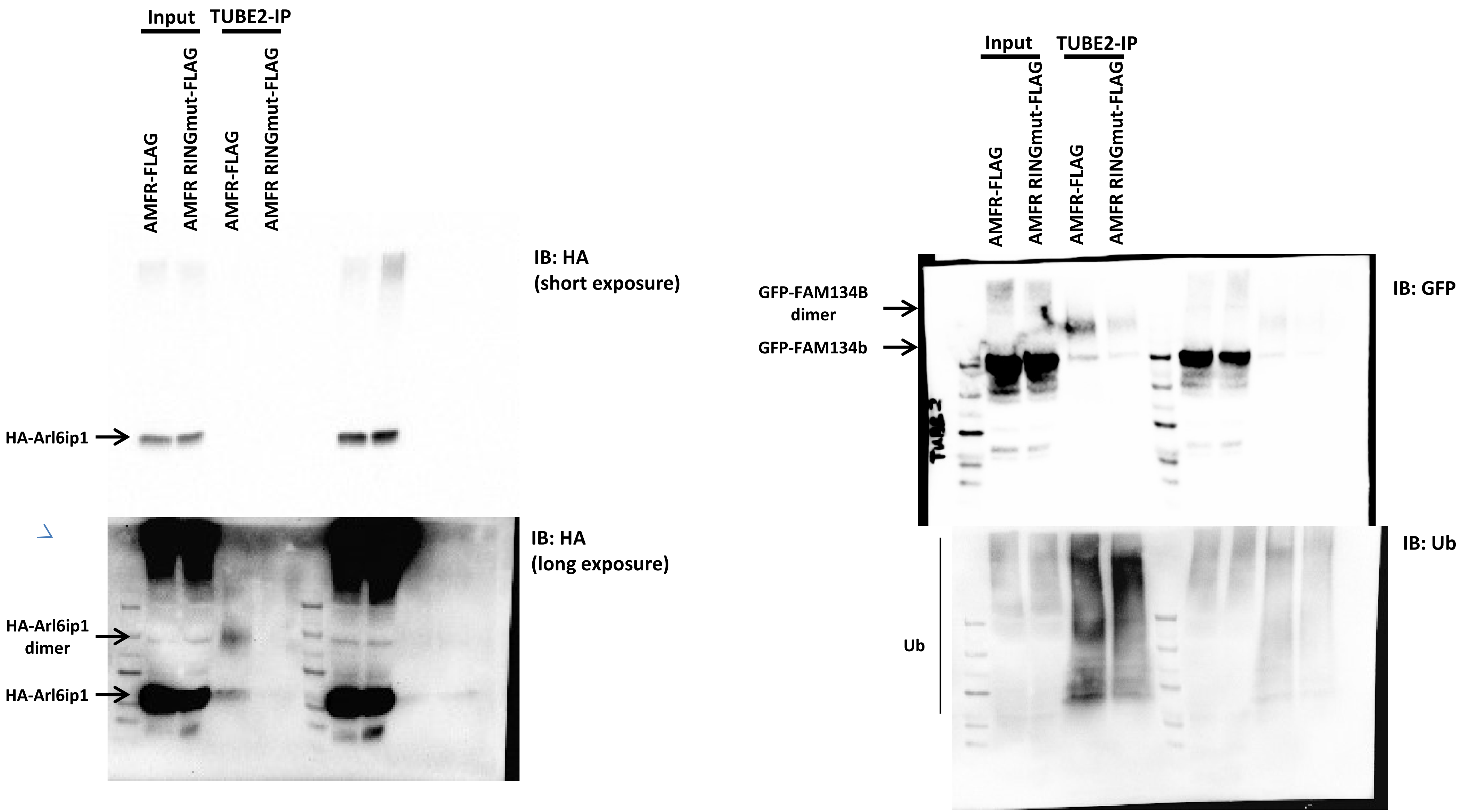
Input
TUBE2-IP
AMFR-FLAG
AMFR RINGmut-FLAG
AMFR-FLAG
AMFR RINGmut-FLAG
IB: HA
(short exposure)
HA-Arl6ip1
IB: HA
(long exposure)
HA-Arl6ip1
dimer
HA-Arl6ip1
Input
TUBE2-IP
AMFR-FLAG
AMFR RINGmut-FLAG
AMFR-FLAG
AMFR RINGmut-FLAG
IB: GFP
GFP-FAM134B
dimer
GFP-FAM134b
IB: Ub
Ub

FIG 4. H

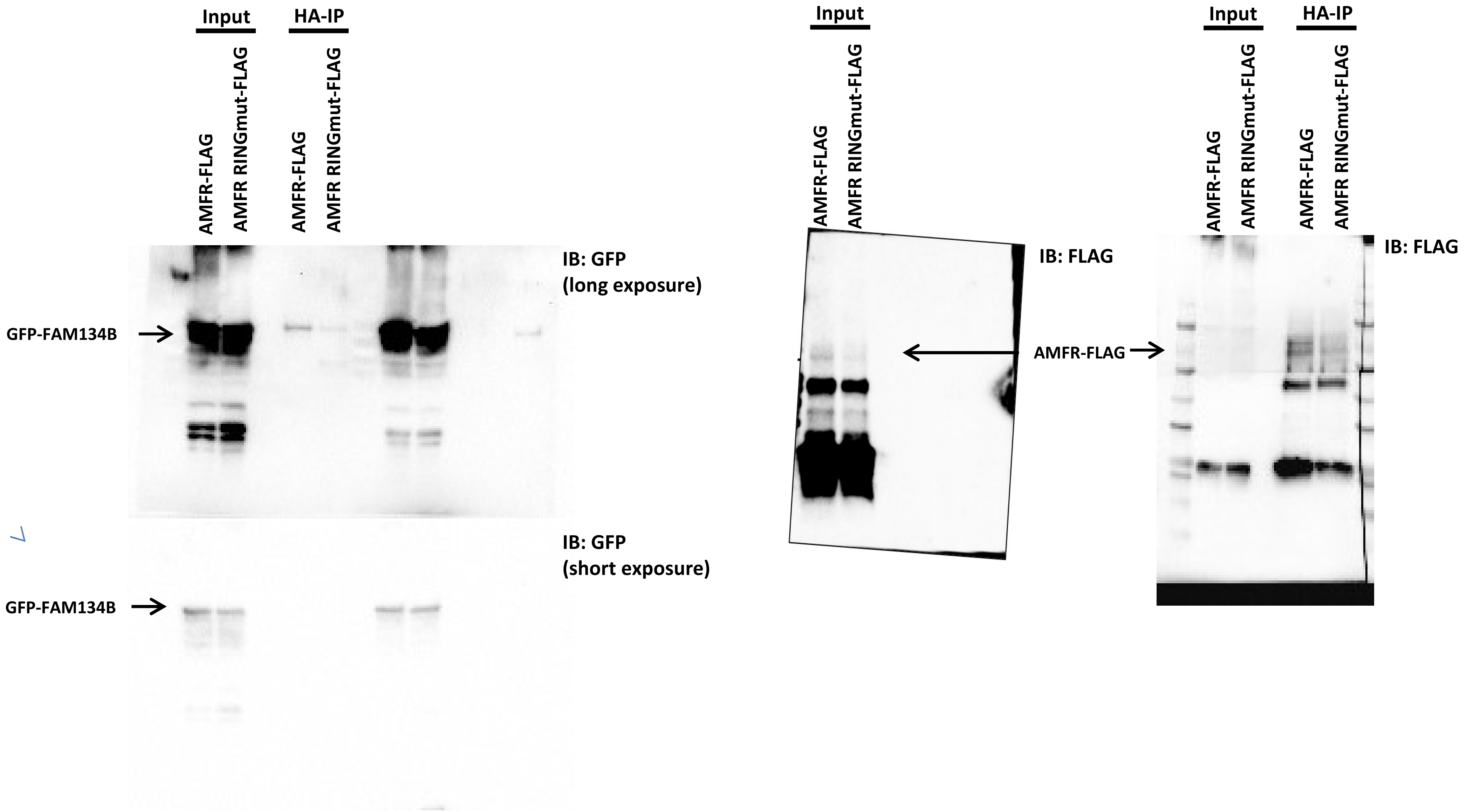
Input
HA-IP
AMFR-FLAG
AMFR RINGmut-FLAG
AMFR-FLAG
AMFR RINGmut-FLAG
IB: GFP
(long exposure)
GFP-FAM134B
IB: GFP
(short exposure)
GFP-FAM134B
Input
AMFR-FLAG
AMFR RINGmut-FLAG
IB: FLAG
AMFR-FLAG
Input
HA-IP
AMFR-FLAG
AMFR RINGmut-FLAG
AMFR-FLAG
AMFR RINGmut-FLAG
IB: FLAG

FIG 4. H

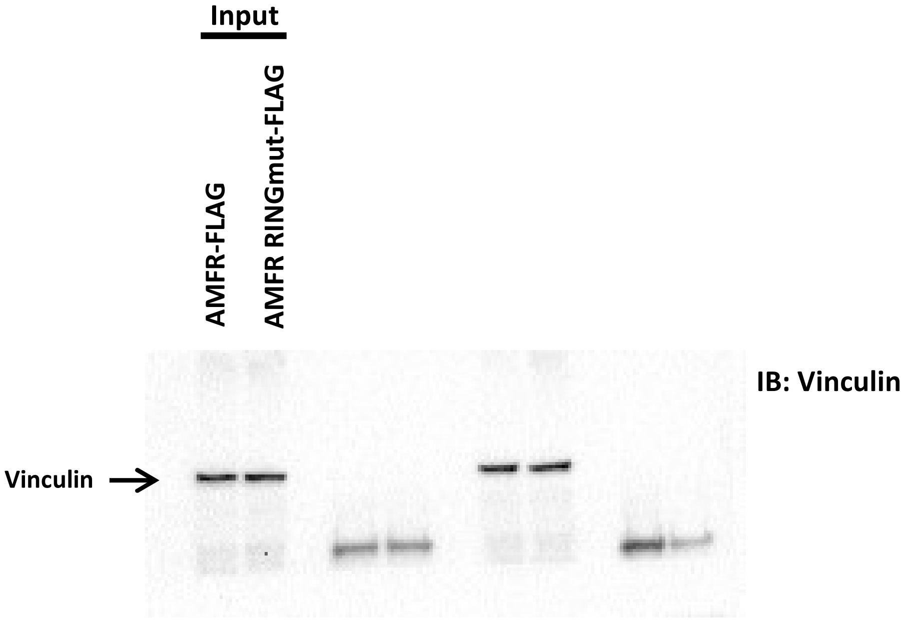
Input
AMFR-FLAG
AMFR RINGmut-FLAG
IB: Vinculin
Vinculin

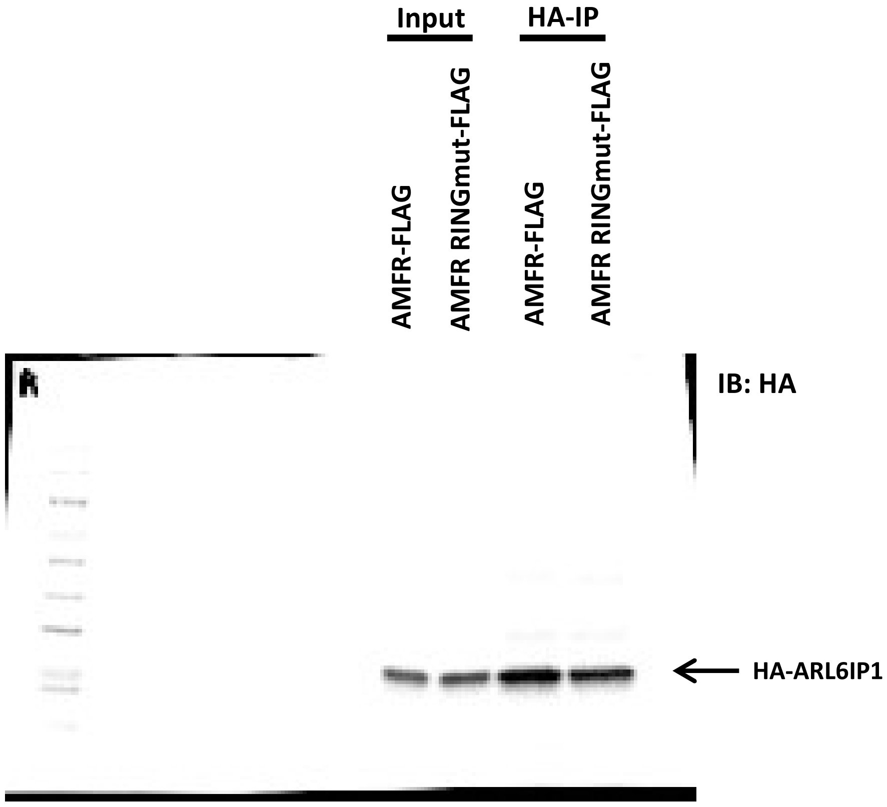
Input
HA-IP
AMFR-FLAG
AMFR RINGmut-FLAG
AMFR-FLAG
AMFR RINGmut-FLAG
IB: HA
HA-ARL6IP1

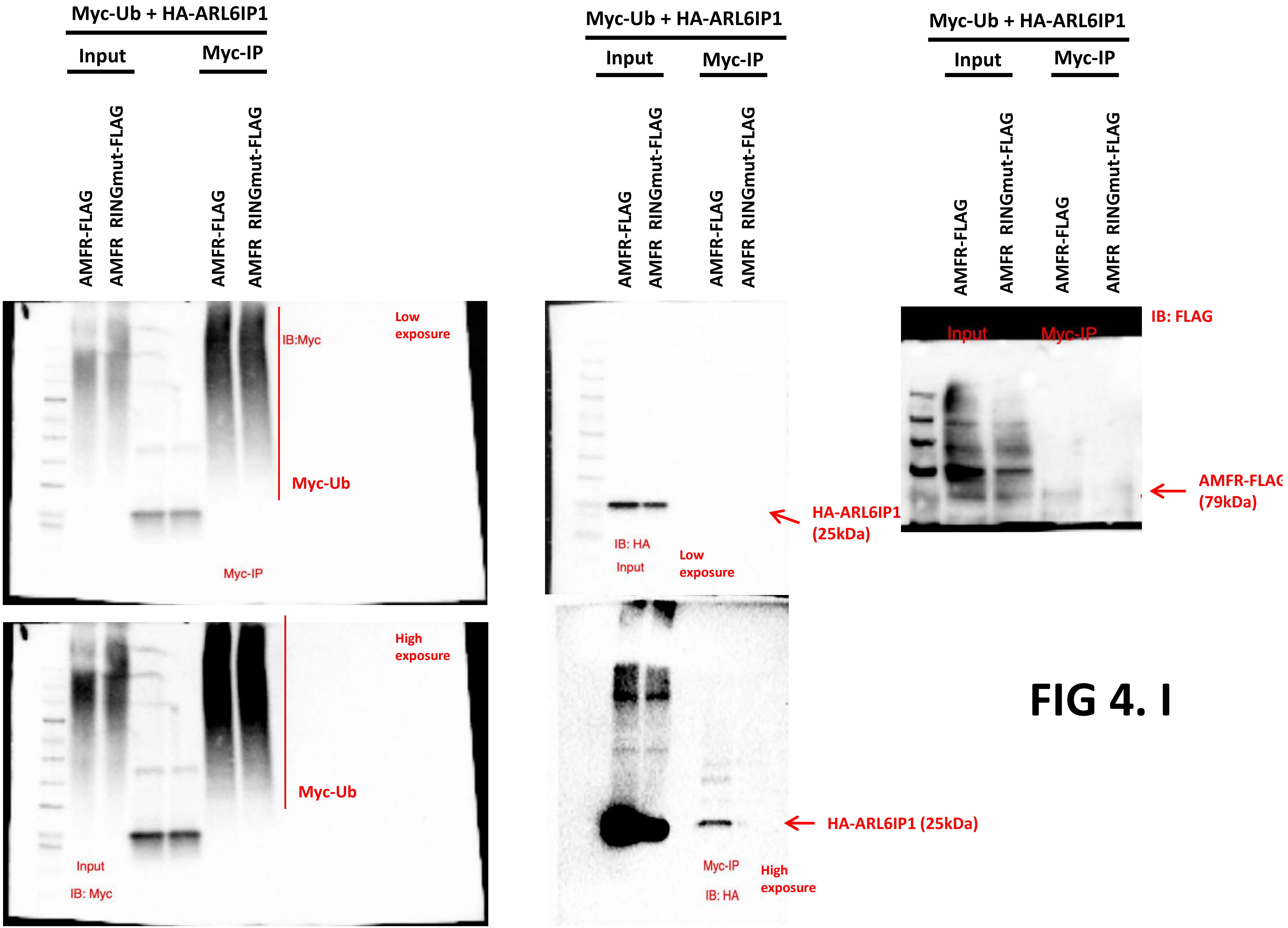

Myc-Ub + HA-ARL6IP1
Input
Myc-IP
AMFR-FLAG
AMFR RINGmut-FLAG
AMFR-FLAG
AMFR RINGmut-FLAG
IB:Myc
Low exposure
Myc-Ub
Myc-IP
High exposure
Myc-Ub
Input
IB: Myc
Myc-Ub + HA-ARL6IP1
Input
Myc-IP
AMFR-FLAG
AMFR RINGmut-FLAG
AMFR-FLAG
AMFR RINGmut-FLAG
HA-ARL6IP1 (25kDa)
IB: HA
Input
Low exposure
HA-ARL6IP1 (25kDa)
Myc-IP
IB: HA
High exposure
Myc-Ub + HA-ARL6IP1
Input
Myc-IP
AMFR-FLAG
AMFR RINGmut-FLAG
AMFR-FLAG
AMFR RINGmut-FLAG
IB: FLAG
Input
Myc-IP
(79kDa)

FIG 4. I

GEL 1

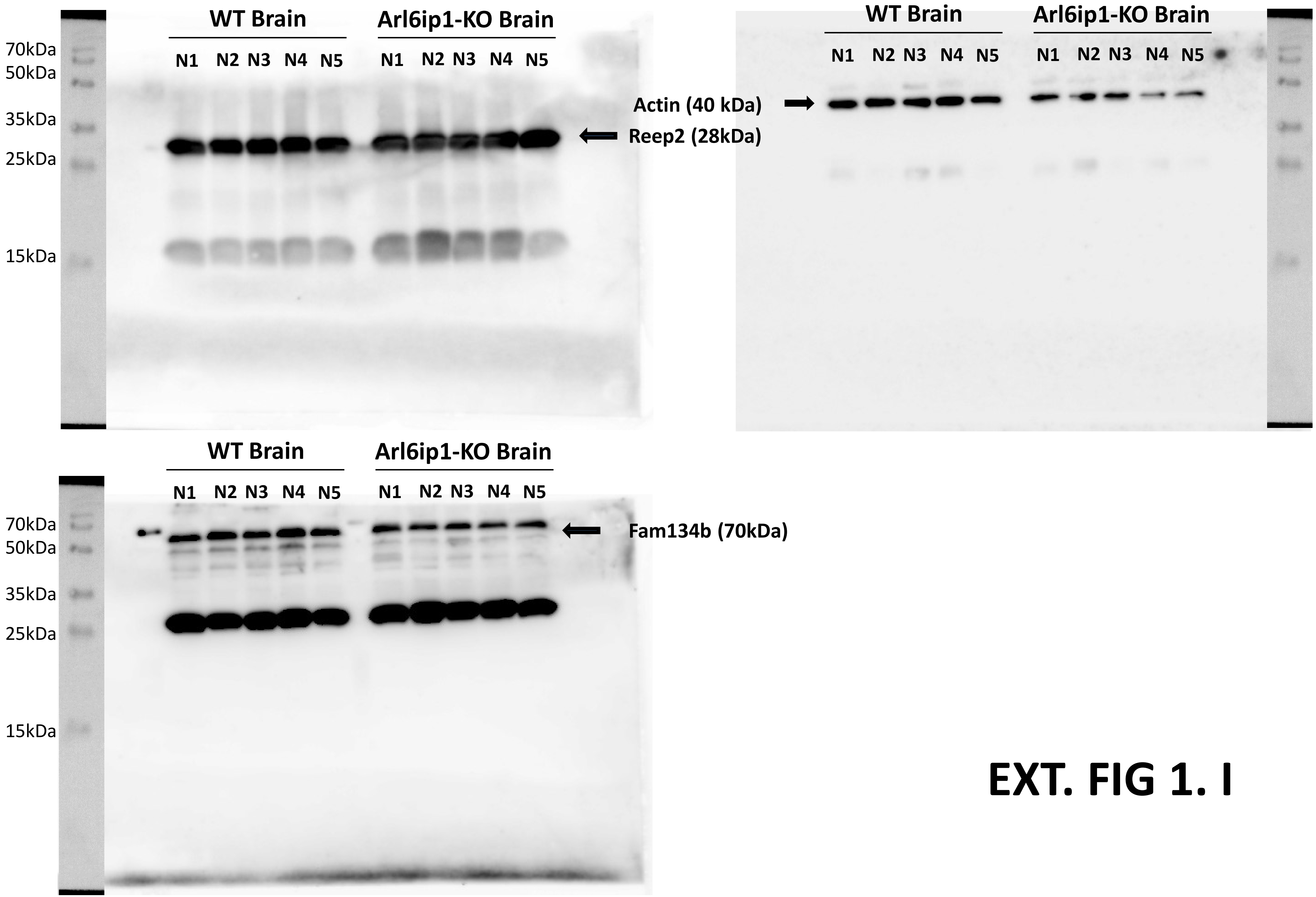

EXT. FIG 1. I

GEL 2

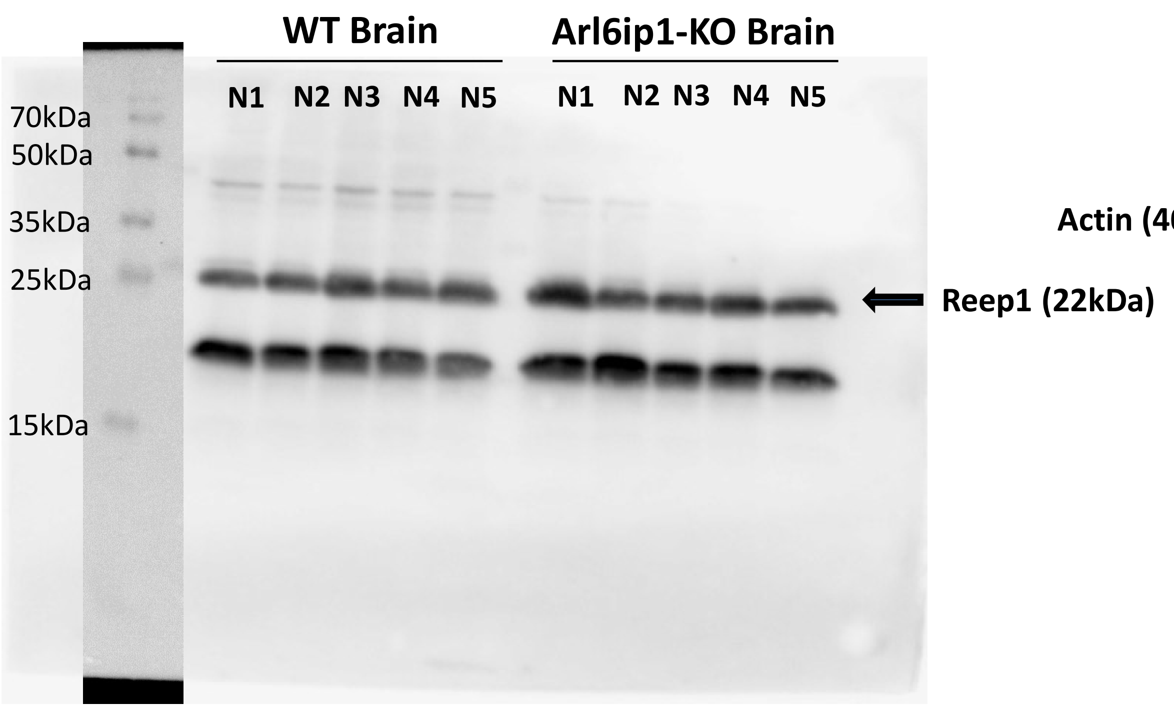

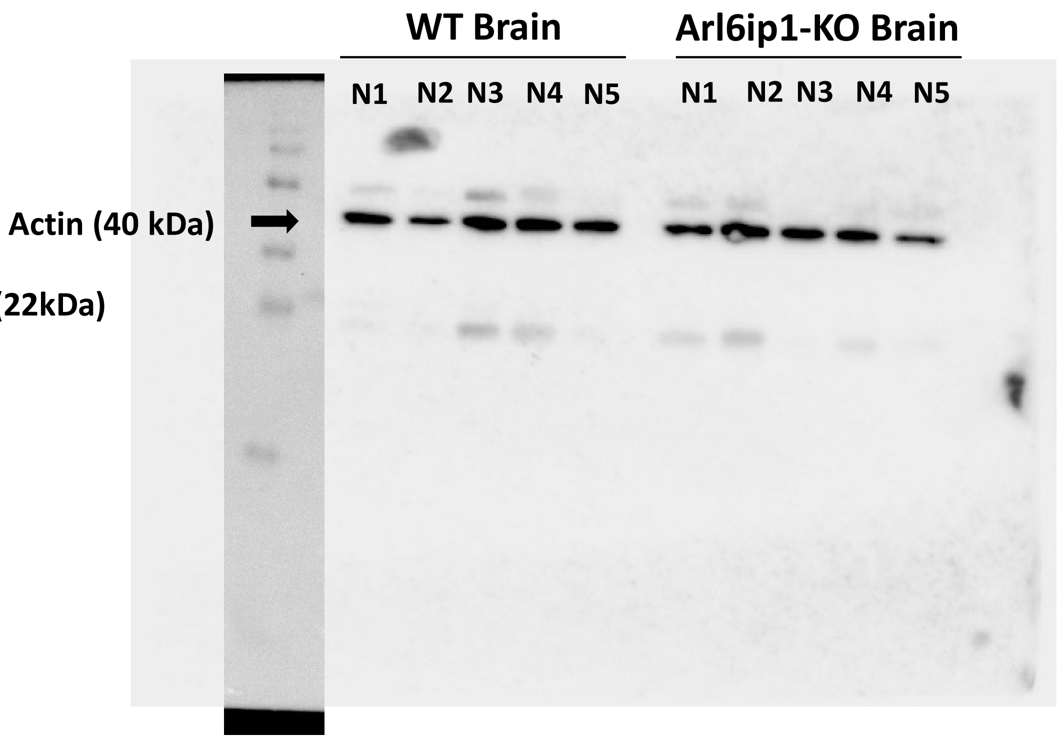

EXT. FIG 1. I

GEL 3

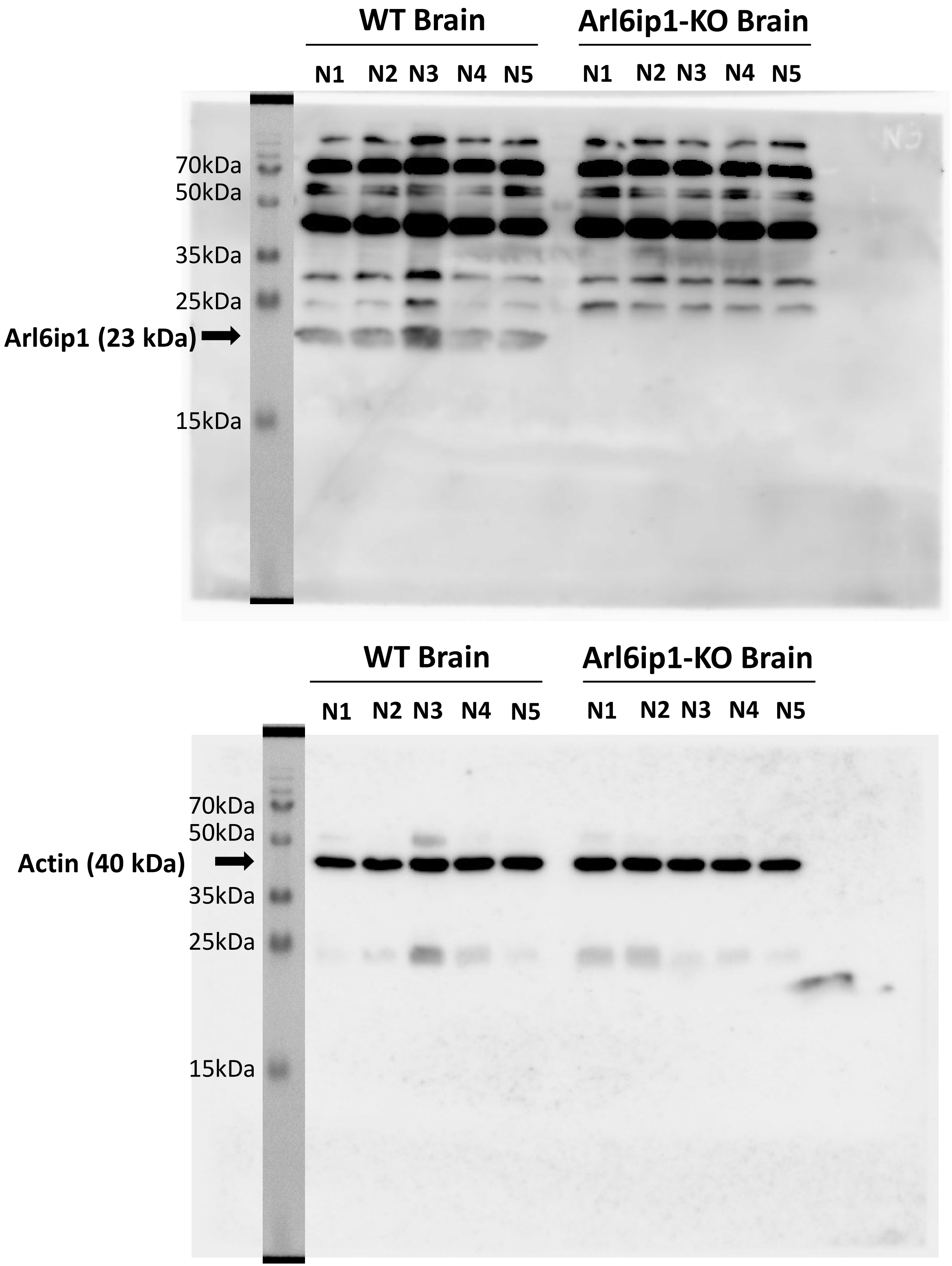

EXT. FIG 1. I

GEL 4

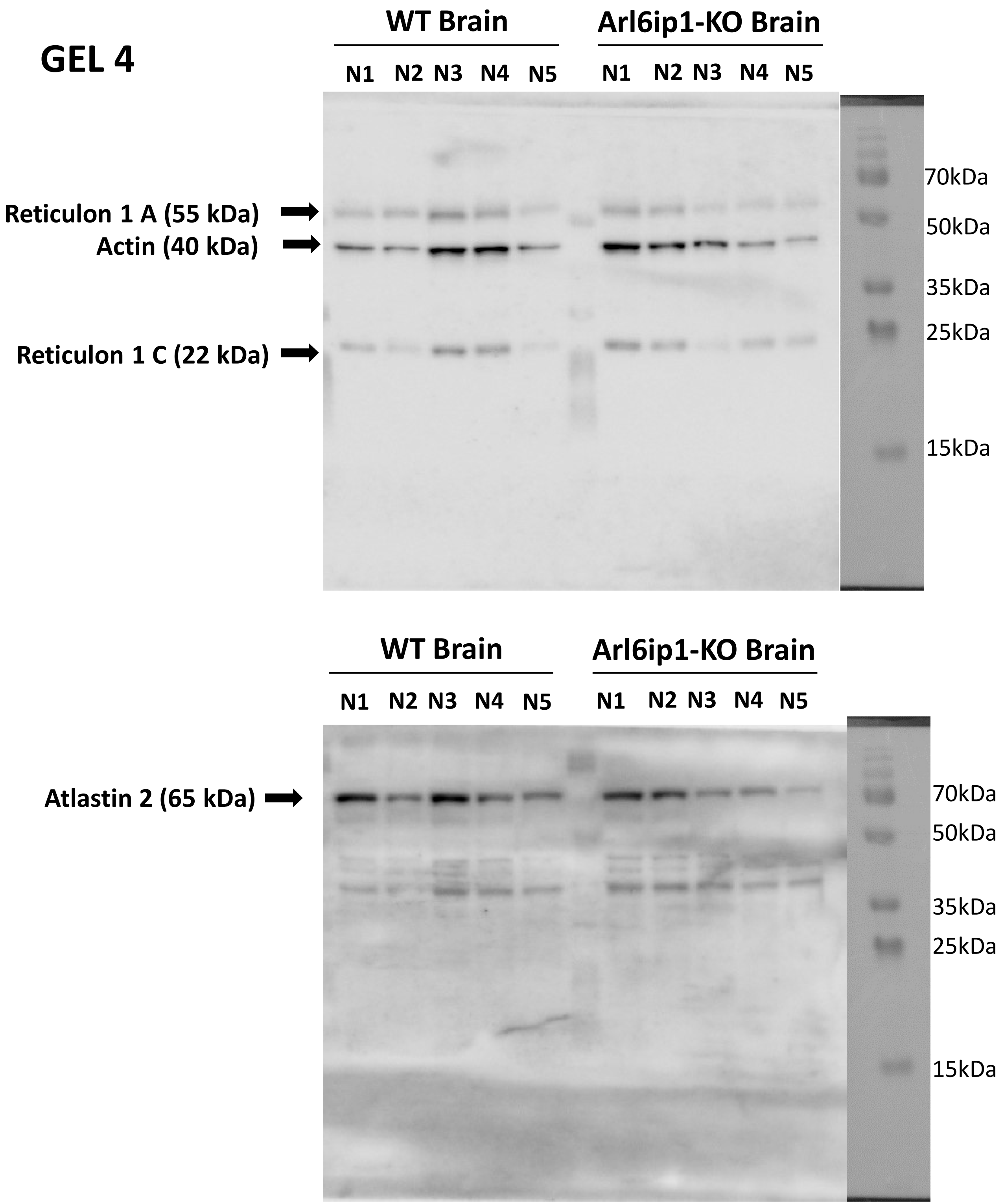

EXT. FIG 1. I

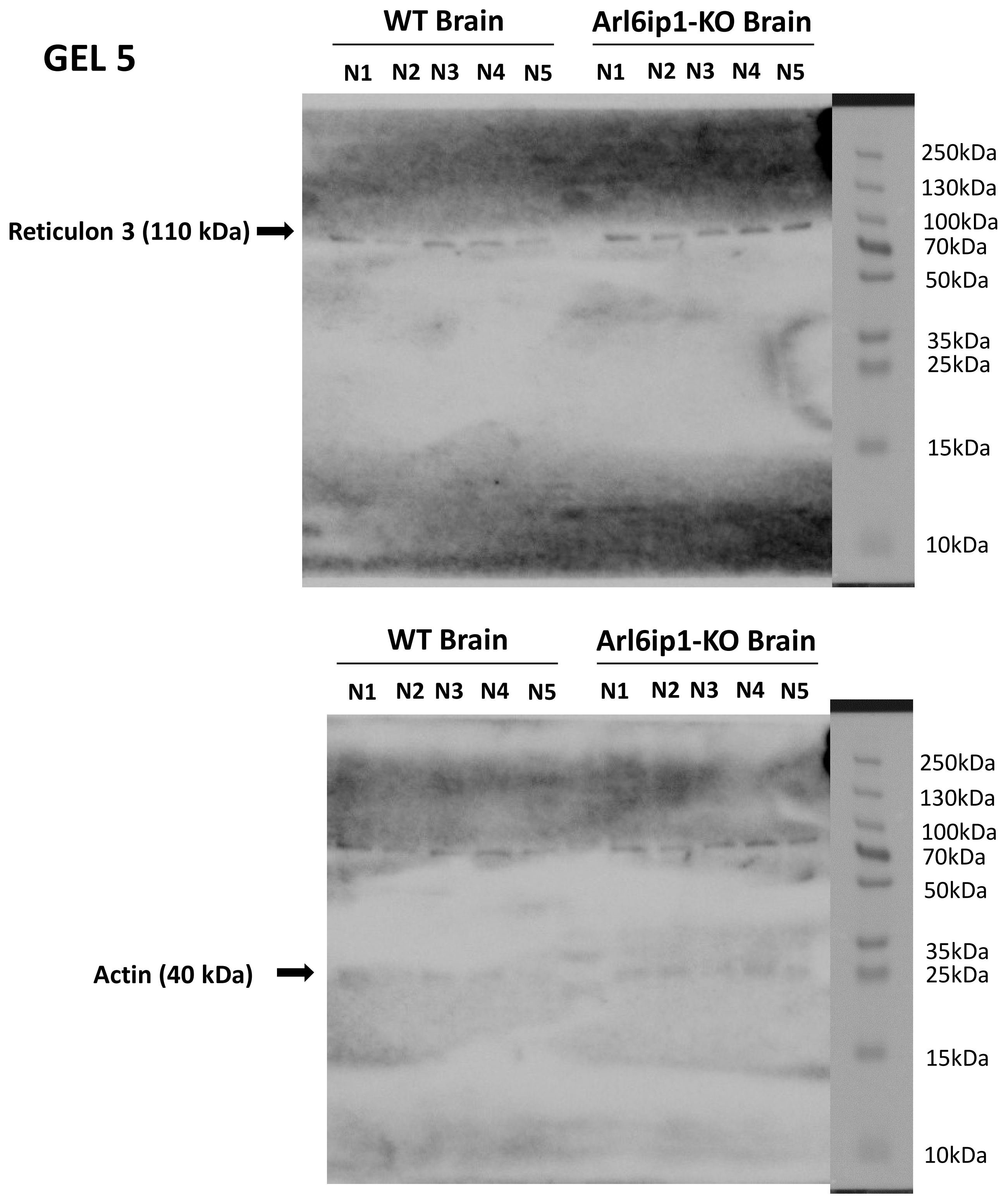
GEL 5
WT Brain
Arl6ip1-KO Brain
N1 N2 N3 N4 N5
N1 N2 N3 N4 N5
Reticulon 3 (110 kDa)
250kDa
130kDa
100kDa
70kDa
50kDa
35kDa
25kDa
15kDa
10kDa
WT Brain
Arl6ip1-KO Brain
N1 N2 N3 N4 N5
N1 N2 N3 N4 N5
Actin (40 kDa)
250kDa
130kDa
100kDa
70kDa
50kDa
35kDa
25kDa
15kDa
10kDa

EXT. FIG 1. I

GEL 6

High exposure

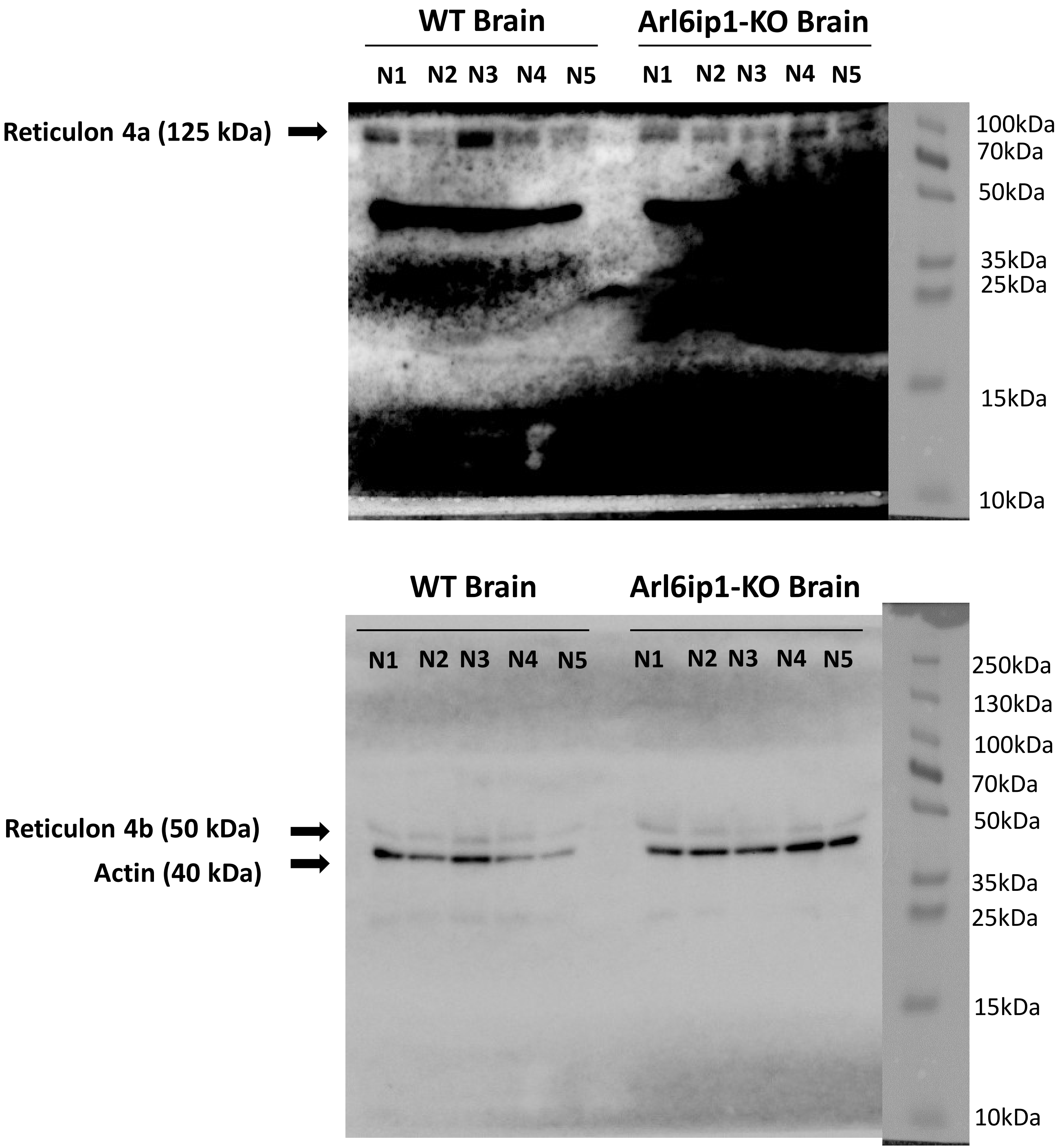

Low exposure

EXT. FIG 1. I

GEL 7

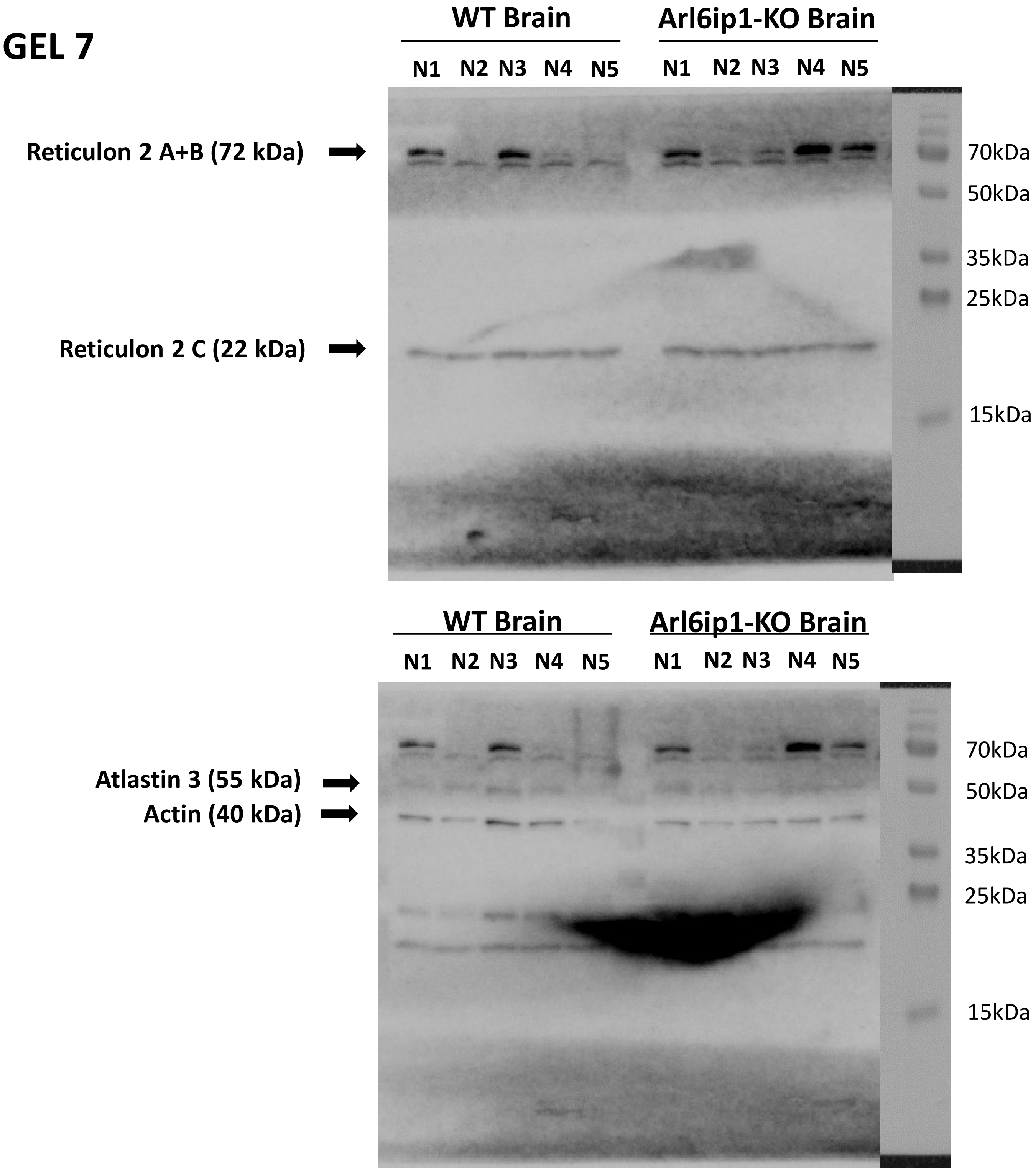

EXT. FIG 1. I

EXT. FIG 2. D

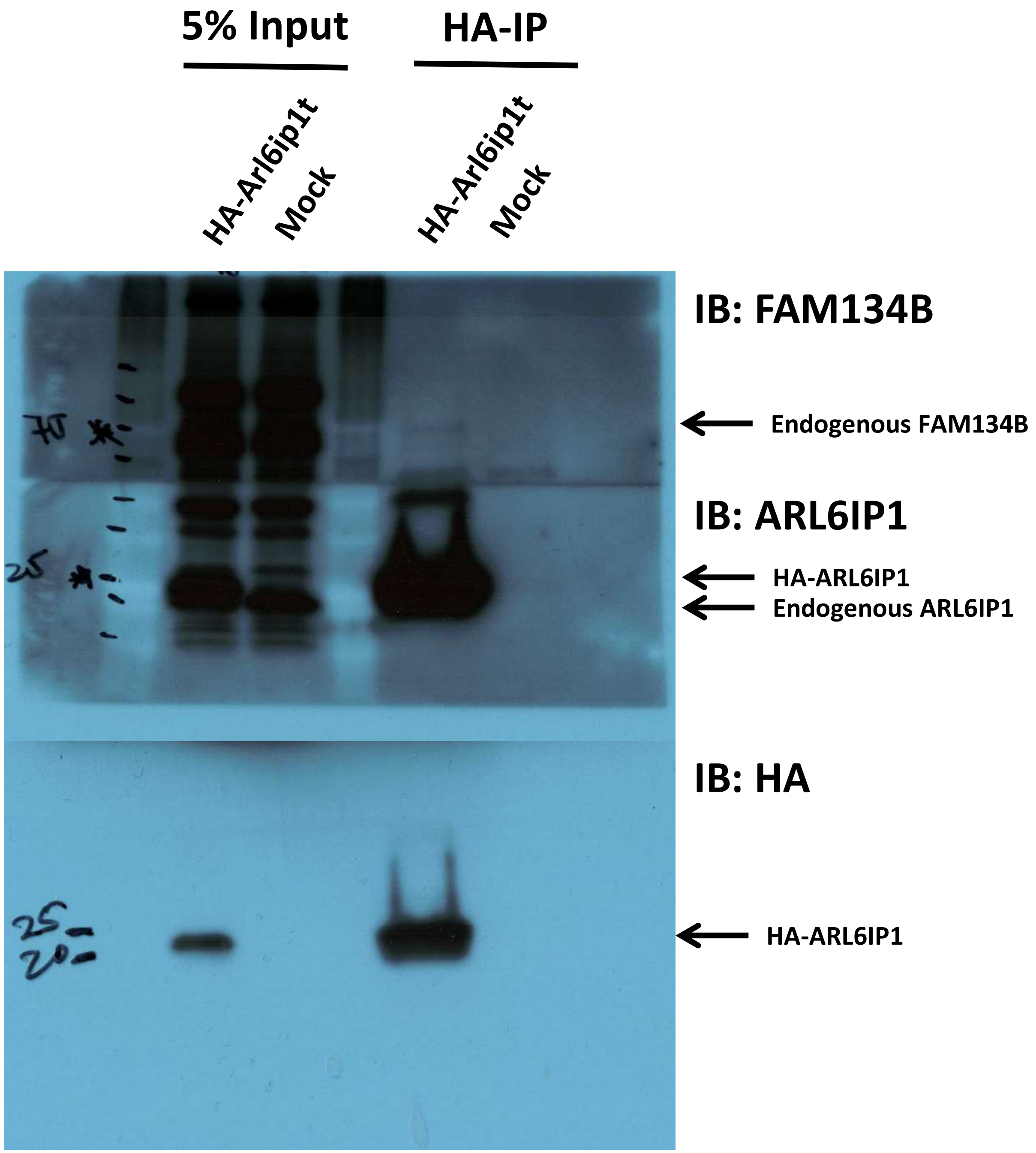
5% Input
HA-IP
HA-Arl6ip1t
Mock
HA-Arl6ip1t
Mock
IB: FAM134B
Endogenous FAM134B
IB: ARL6IP1
HA-ARL6IP1
Endogenous ARL6IP1
IB: HA
HA-ARL6IP1
25
20

EXT. FIG 2. E

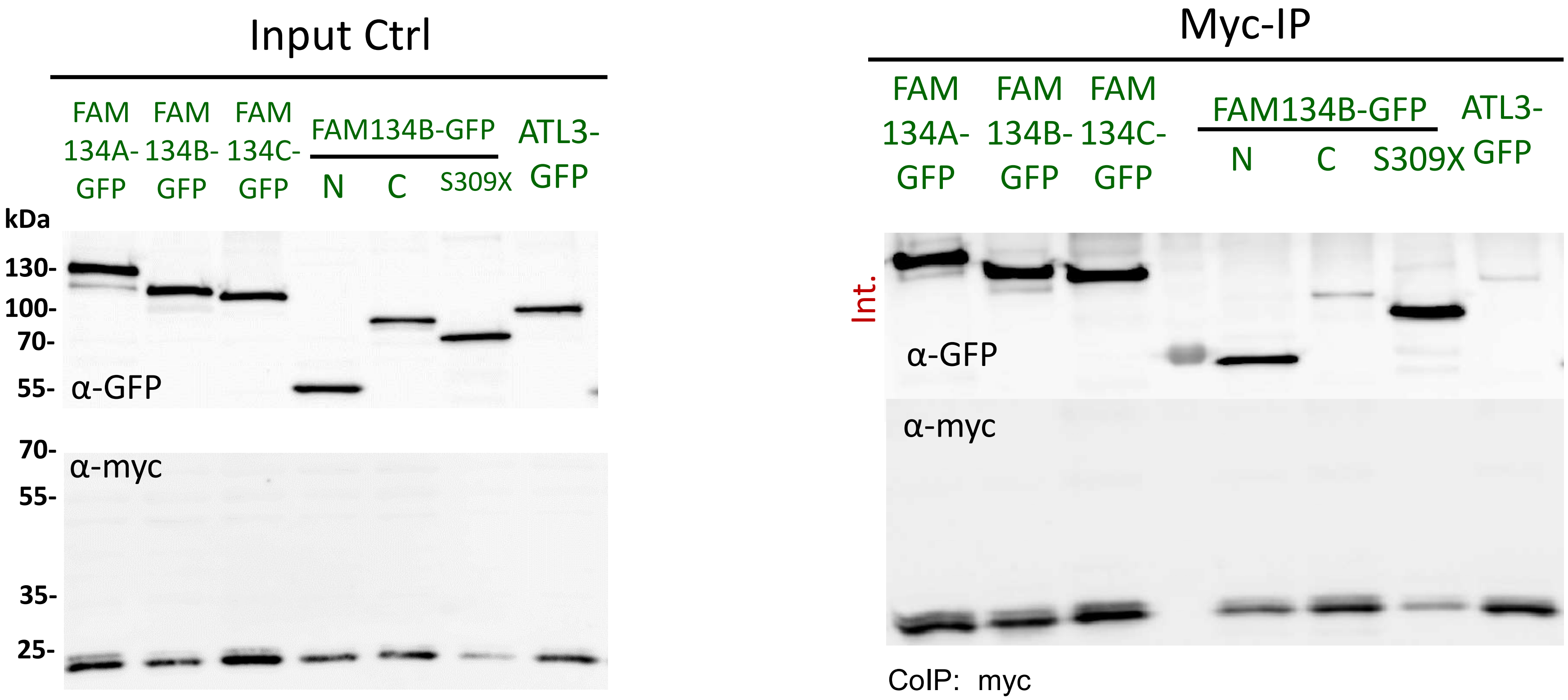
Input Ctrl
FAM 134A-GFP
FAM 134B-GFP
FAM 134C-GFP
FAM134B-GFP
N
C
S309X
ATL3-GFP
kDa
130-
100-
70-
55-
α-GFP
70-
55-
35-
25-
α-myc
Myc-IP
FAM 134A-GFP
FAM 134B-GFP
FAM 134C-GFP
FAM134B-GFP
N
C
S309X
ATL3-GFP
Int.
α-GFP
α-myc
CoIP: myc

EXT. FIG 2. F

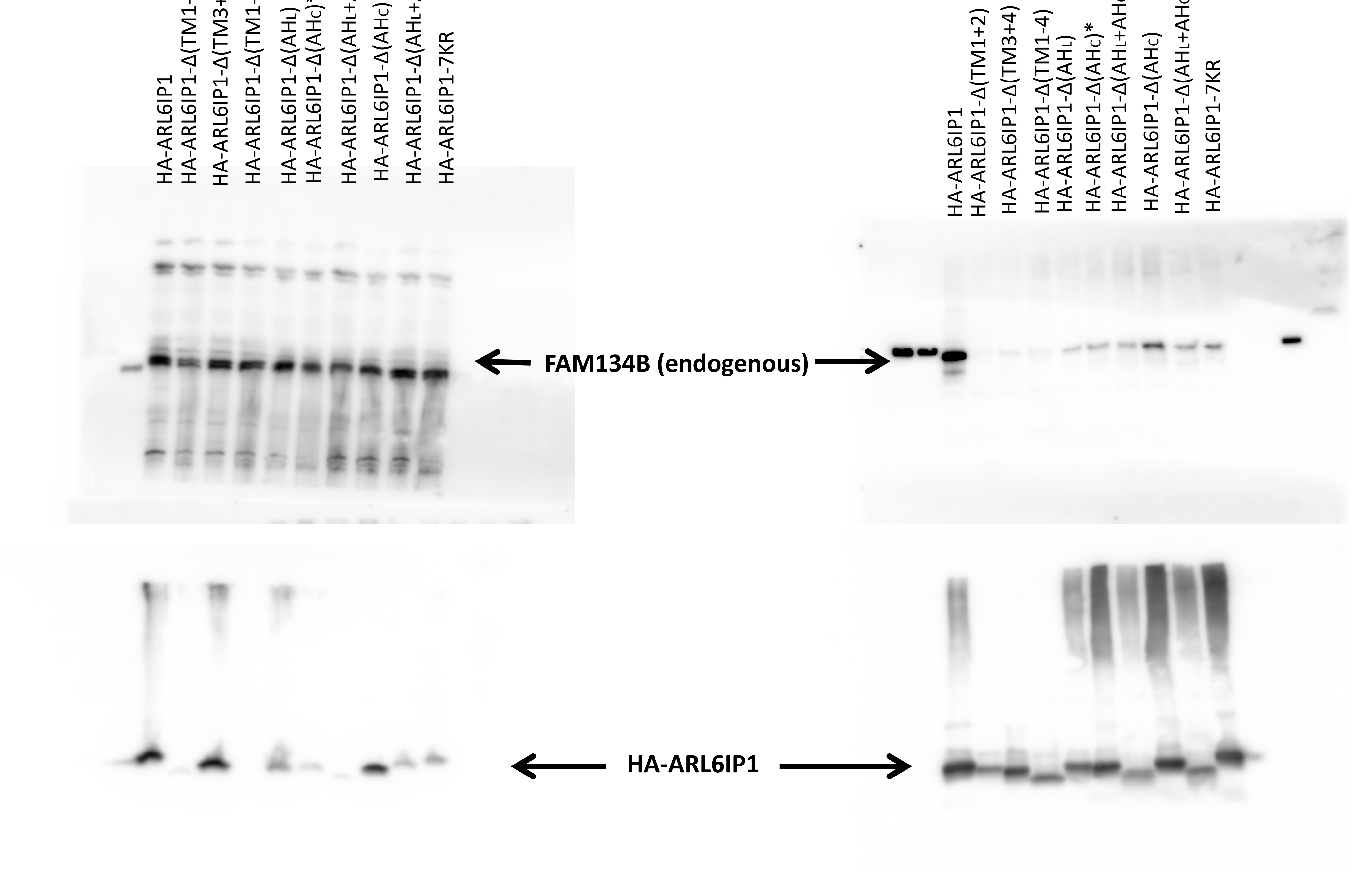
INPUT
HA IP
HA-ARL6IP1
HA-ARL6IP1-Δ(TM1+2)
HA-ARL6IP1-Δ(TM3+4)
HA-ARL6IP1-Δ(TM1-4)
HA-ARL6IP1-Δ(AHL)
HA-ARL6IP1-Δ(AHC)*
HA-ARL6IP1-Δ(AHL+AHC)*
HA-ARL6IP1-Δ(AHC)
HA-ARL6IP1-Δ(AHL+AHC)
HA-ARL6IP1-7KR
FAM134B (endogenous)
HA-ARL6IP1

INPUT

EXT. FIG 2. F

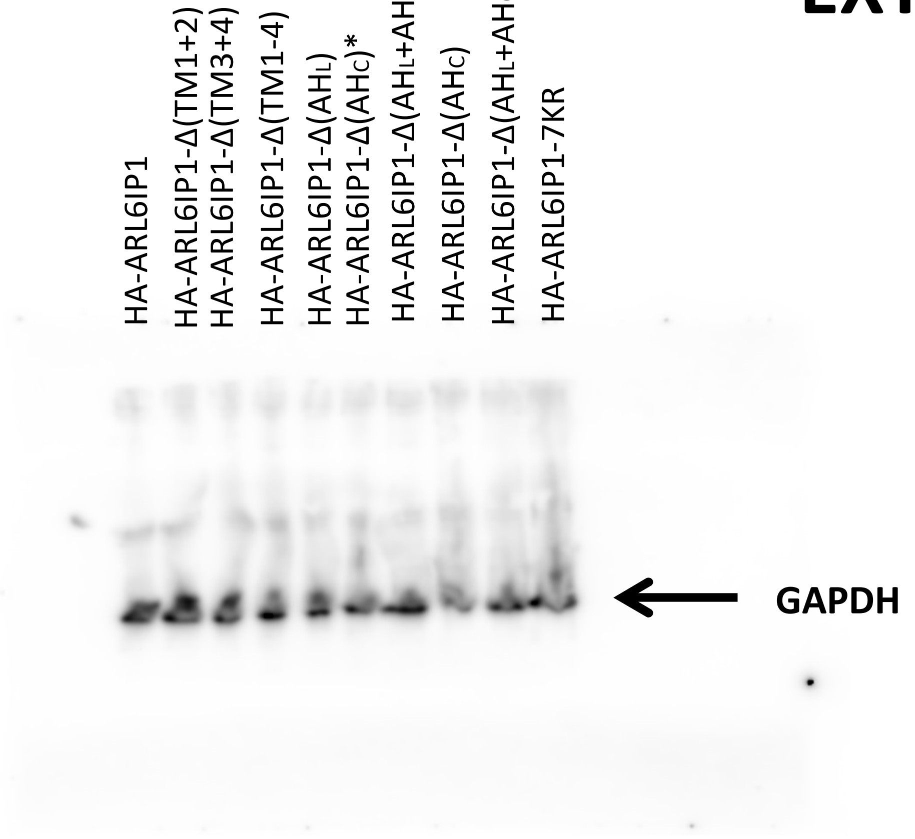

EXT. FIG 2. G

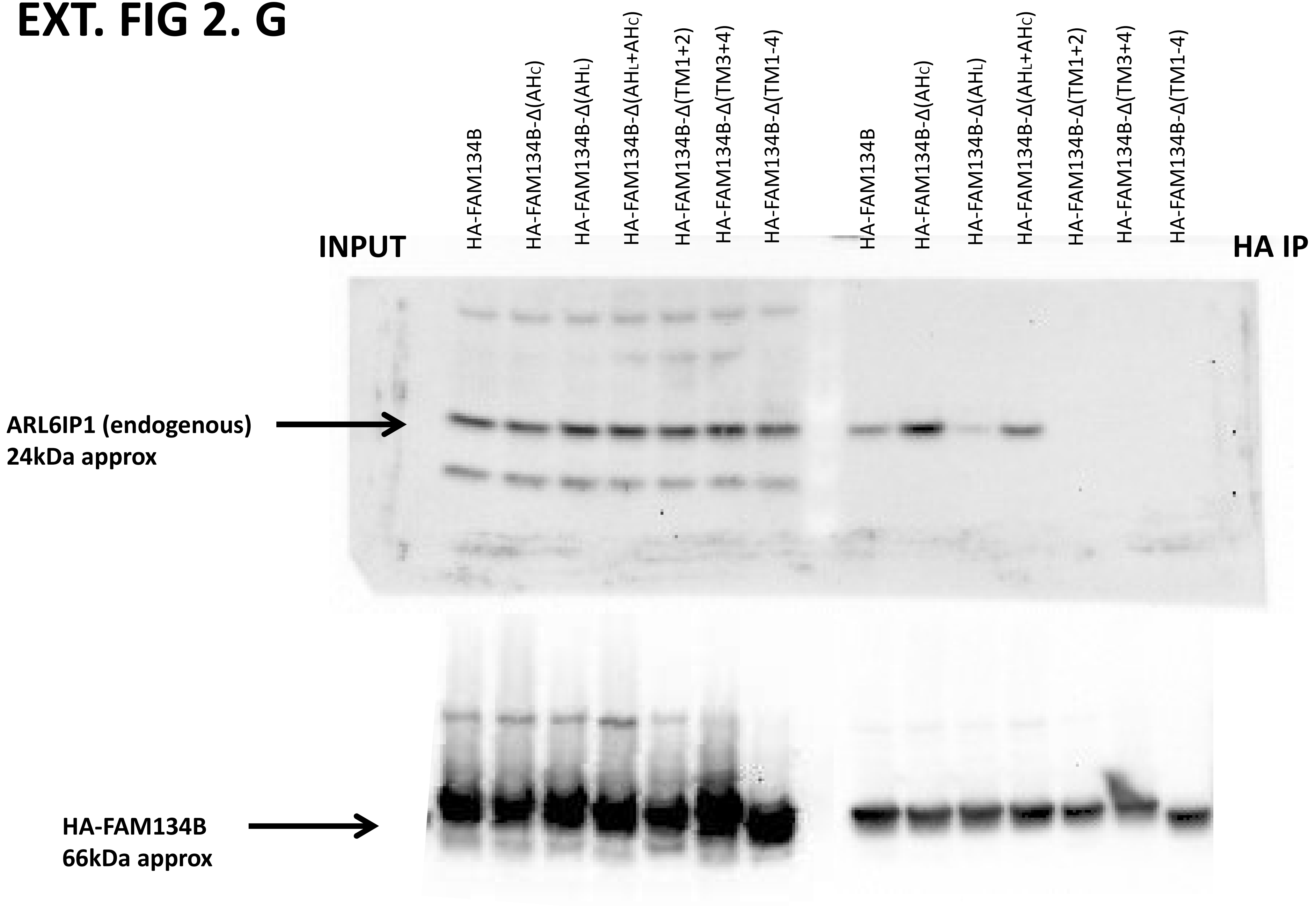
INPUT
HA-FAM134B
HA-FAM134B-Δ(AHC)
HA-FAM134B-Δ(AHL)
HA-FAM134B-Δ(AHL+AHC)
HA-FAM134B-Δ(TM1+2)
HA-FAM134B-Δ(TM3+4)
HA-FAM134B-Δ(TM1-4)
HA-FAM134B
HA-FAM134B-Δ(AHC)
HA-FAM134B-Δ(AHL)
HA-FAM134B-Δ(AHL+AHC)
HA-FAM134B-Δ(TM1+2)
HA-FAM134B-Δ(TM3+4)
HA-FAM134B-Δ(TM1-4)
HA IP
ARL6IP1 (endogenous)
24kDa approx
HA-FAM134B
66kDa approx

EXT. FIG 3. E

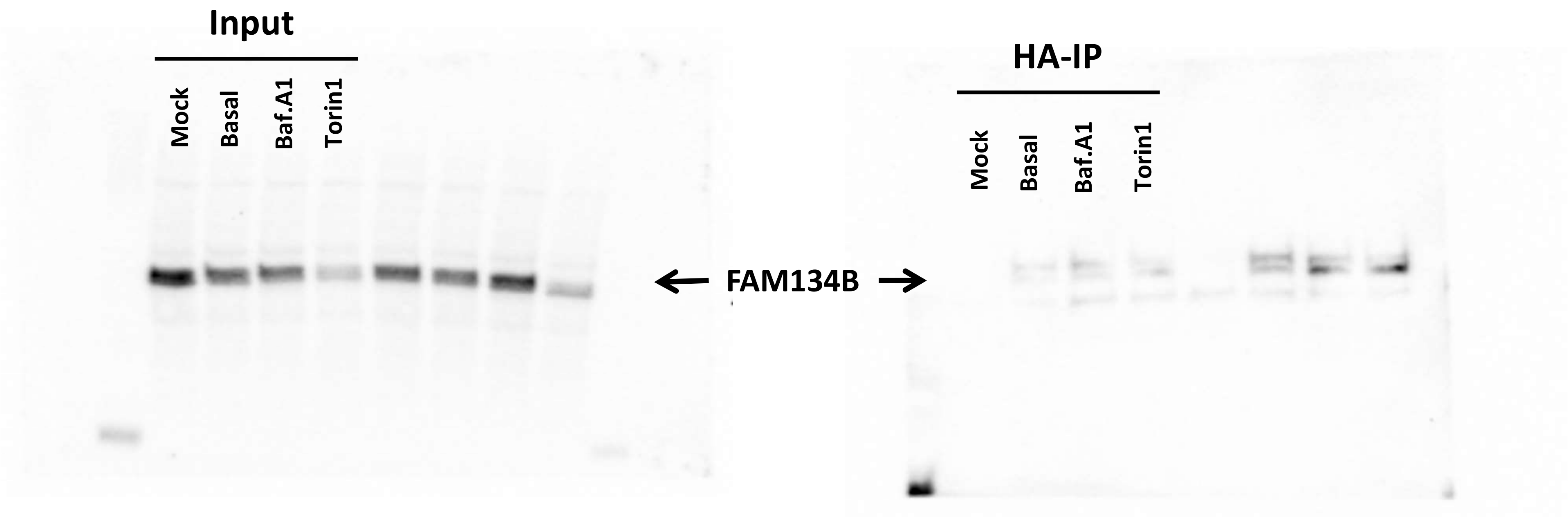
Input
Mock
Basal
Baf.A1
Torin1
FAM134B
HA-IP
Mock
Basal
Baf.A1
Torin1

EXT. FIG 3. E

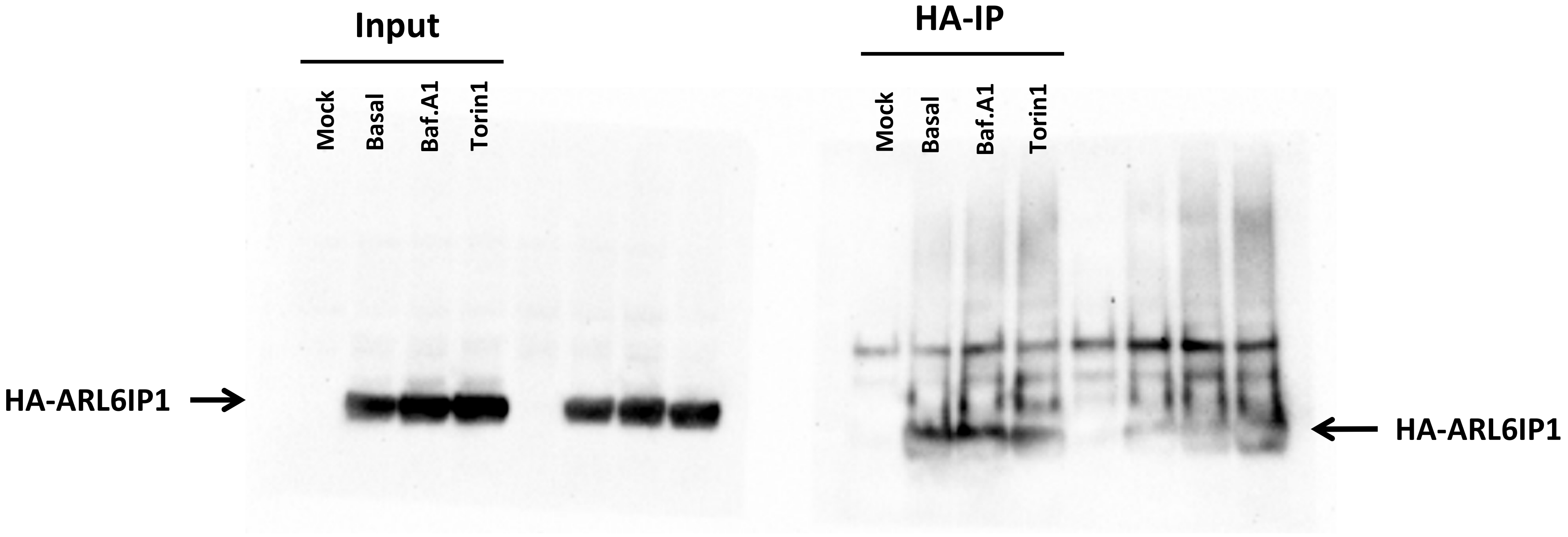

Input
HA-IP
Mock
Basal
Baf.A1
Torin1
Mock
Basal
Baf.A1
Torin1
HA-ARL6IP1
HA-ARL6IP1

EXT. FIG 3. E

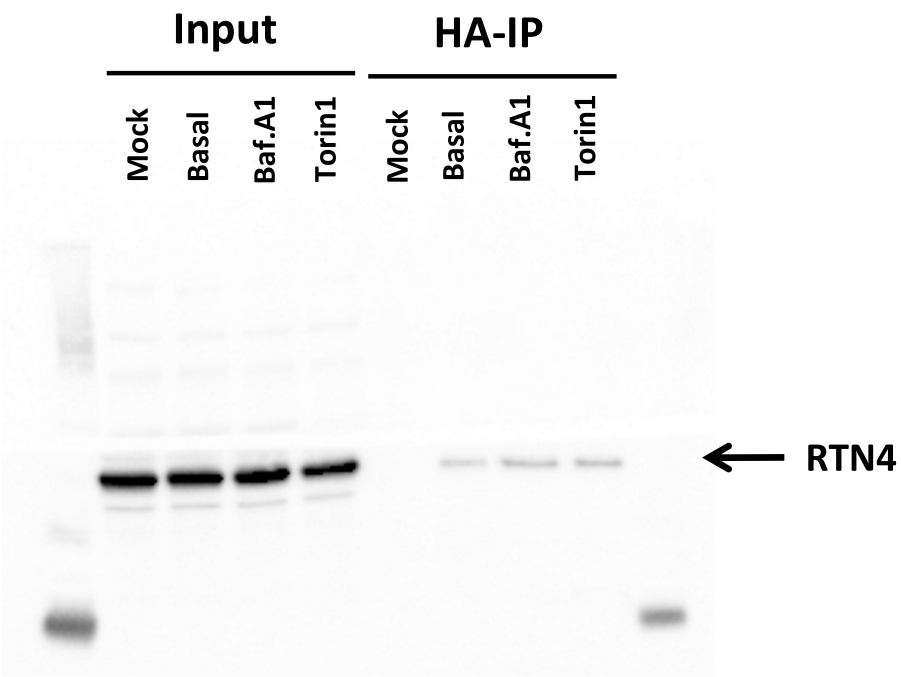
Input
HA-IP
Mock
Basal
Baf.A1
Torin1
Mock
Basal
Baf.A1
Torin1
RTN4

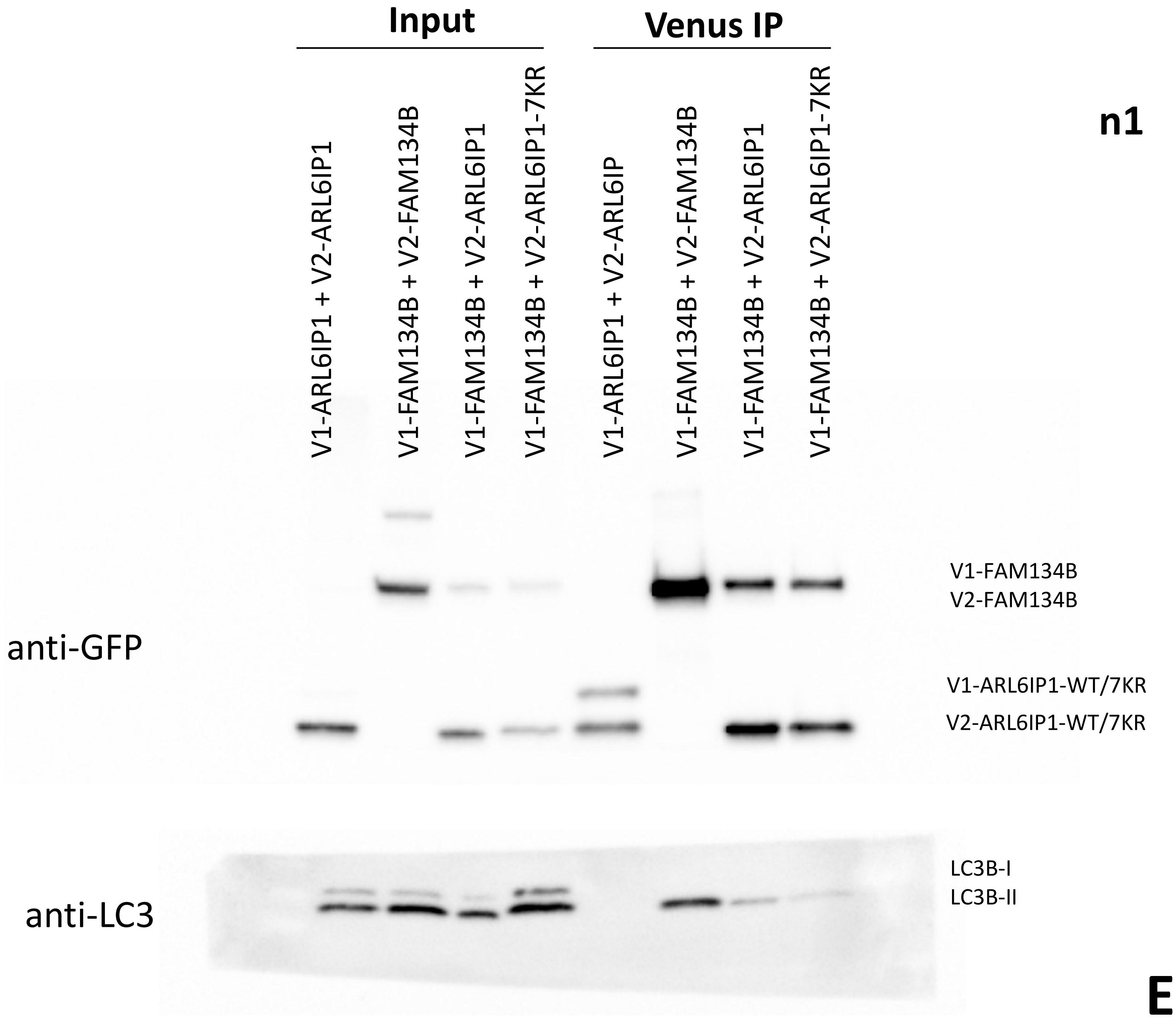
Input
Venus IP
V1-ARL6IP1 + V2-ARL6IP1
V1-FAM134B + V2-FAM134B
V1-FAM134B + V2-ARL6IP1
V1-FAM134B + V2-ARL6IP1-7KR
V1-ARL6IP1 + V2-ARL6IP
V1-FAM134B + V2-FAM134B
V1-FAM134B + V2-ARL6IP1
V1-FAM134B + V2-ARL6IP1-7KR
n1
V1-FAM134B
V2-FAM134B
anti-GFP
V1-ARL6IP1-WT/7KR
V2-ARL6IP1-WT/7KR
LC3B-I
LC3B-II
anti-LC3

EXT. FIG 5. D

n2

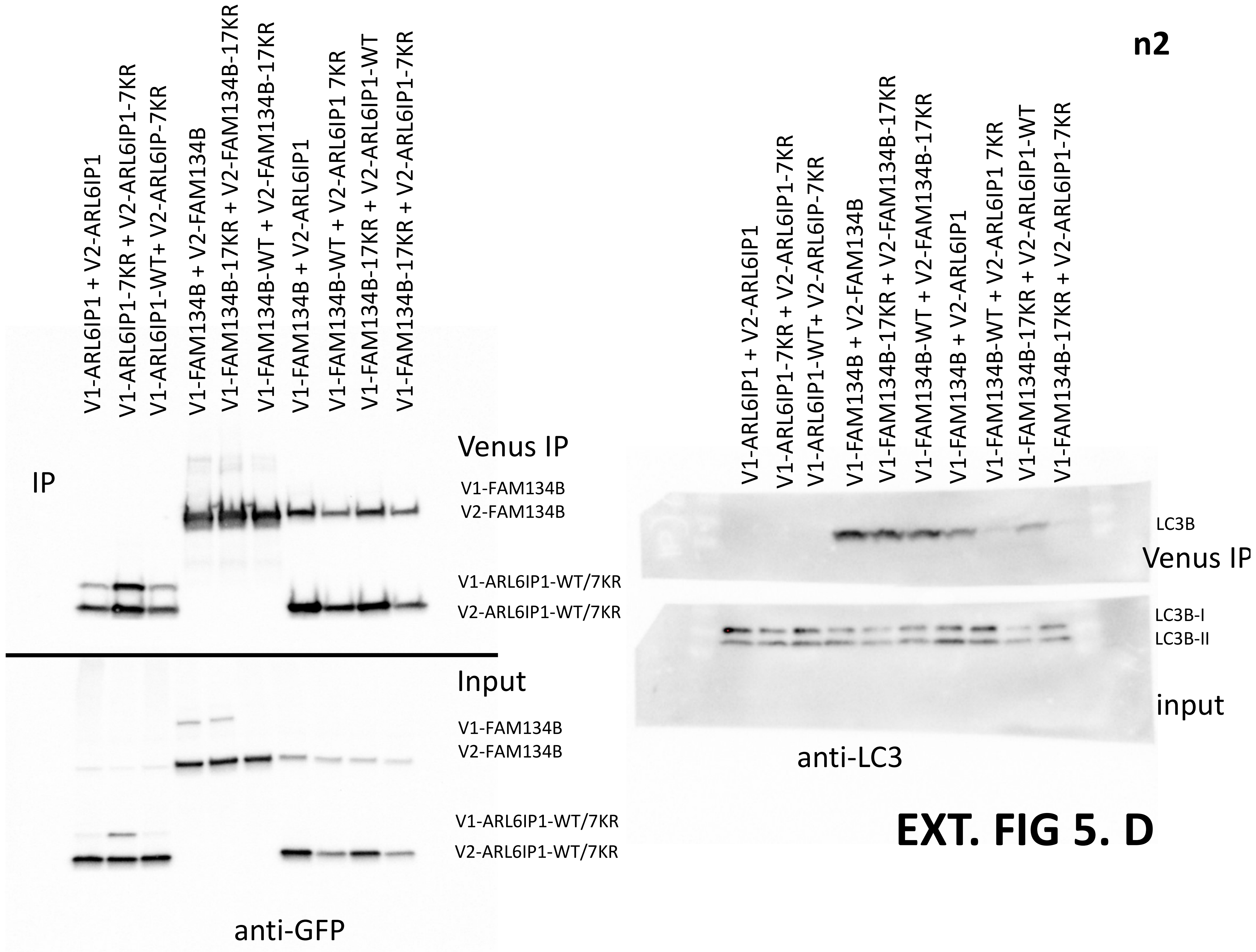
V1-ARL6IP1 + V2-ARL6IP1
V1-ARL6IP1-7KR + V2-ARL6IP1-7KR
V1-ARL6IP1-WT+ V2-ARL6IP-7KR
V1-FAM134B + V2-FAM134B
V1-FAM134B-17KR + V2-FAM134B-17KR
V1-FAM134B-WT + V2-FAM134B-17KR
V1-FAM134B + V2-ARL6IP1
V1-FAM134B-WT + V2-ARL6IP1 7KR
V1-FAM134B-17KR + V2-ARL6IP1-WT
V1-FAM134B-17KR + V2-ARL6IP1-7KR
IP
Venus IP
V1-FAM134B
V2-FAM134B
V1-ARL6IP1-WT/7KR
V2-ARL6IP1-WT/7KR
Input
V1-FAM134B
V2-FAM134B
V1-ARL6IP1-WT/7KR
V2-ARL6IP1-WT/7KR
anti-GFP
LC3B
Venus IP
LC3B-I
LC3B-II
input
anti-LC3

EXT. FIG 5. D

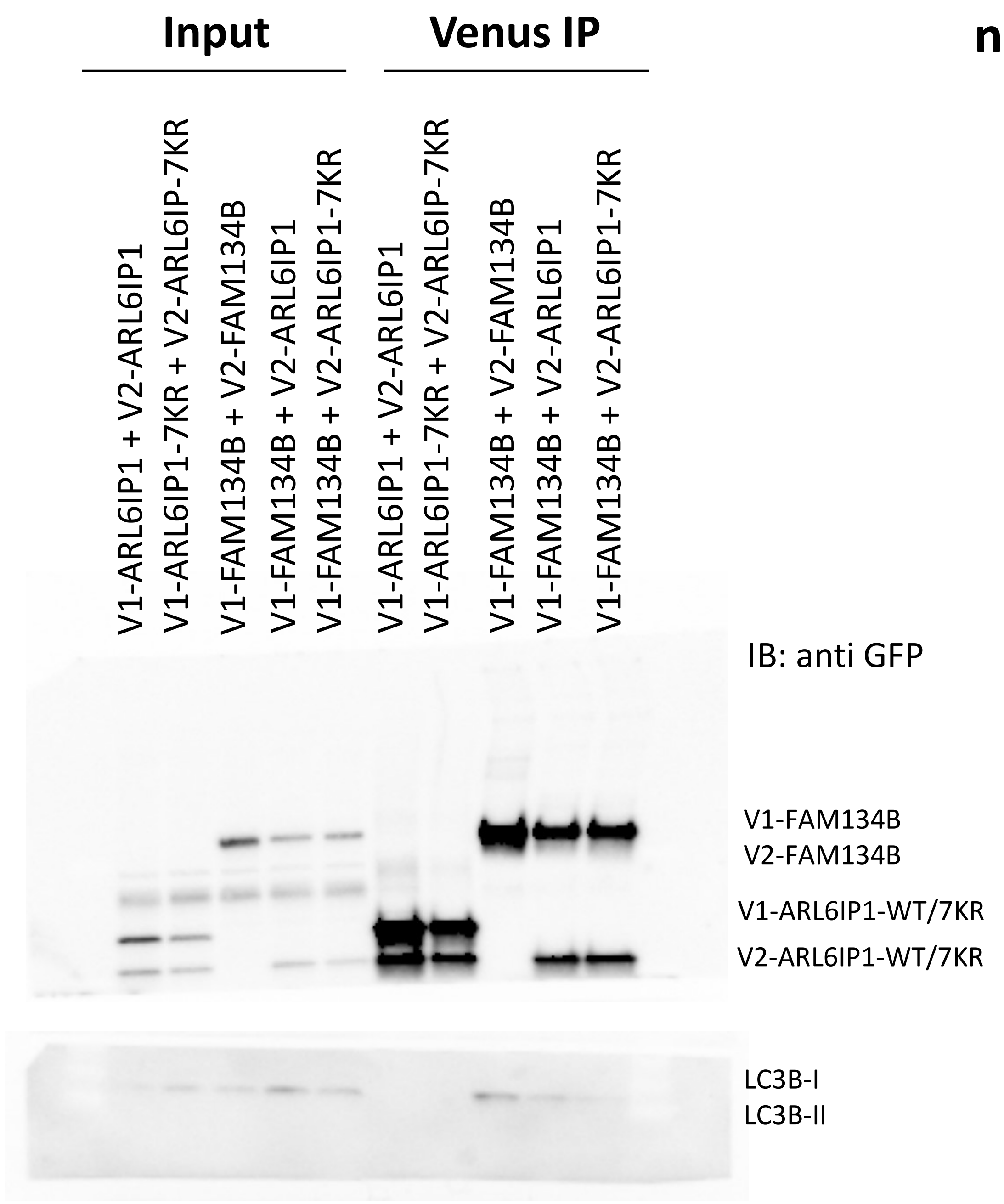

n3 shown in the figure.

EXT. FIG 5. D

EXT. FIG 5. F

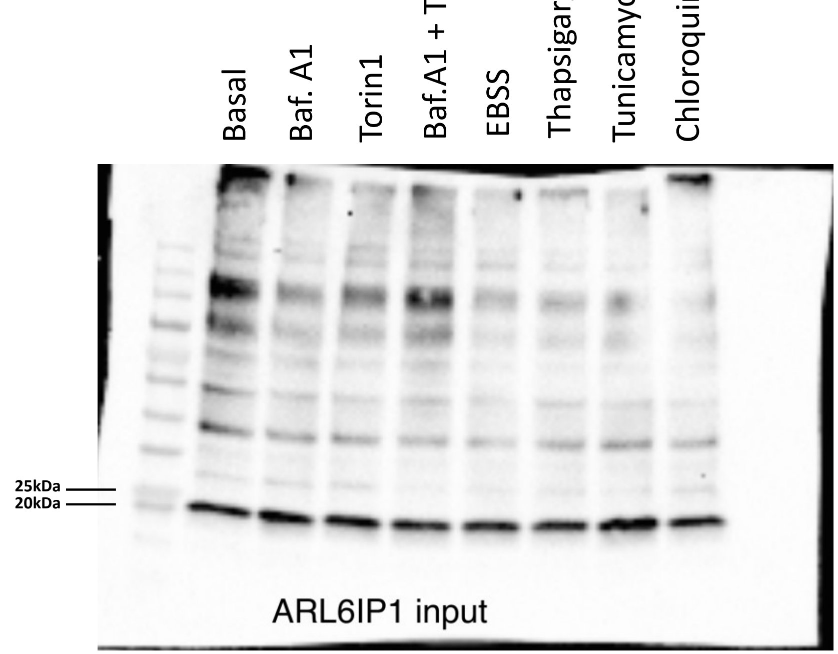
Basal
Baf. A1
Torin1
Baf.A1 + Torin1
EBSS
Thapsigargin
Tunicamycin
Chloroquin
25kDa
20kDa
ARL6IP1 input

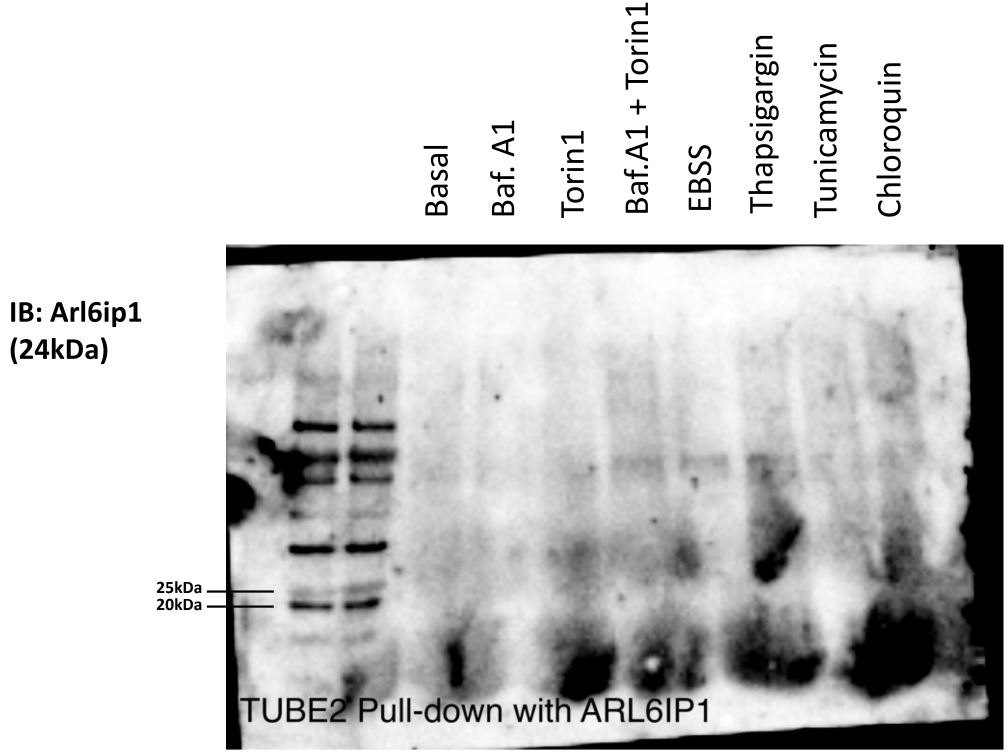
IB: Arl6ip1
(24kDa)
Basal
Baf. A1
Torin1
Baf.A1 + Torin1
EBSS
Thapsigargin
Tunicamycin
Chloroquin
25kDa
20kDa
TUBE2 Pull-down with ARL6IP1

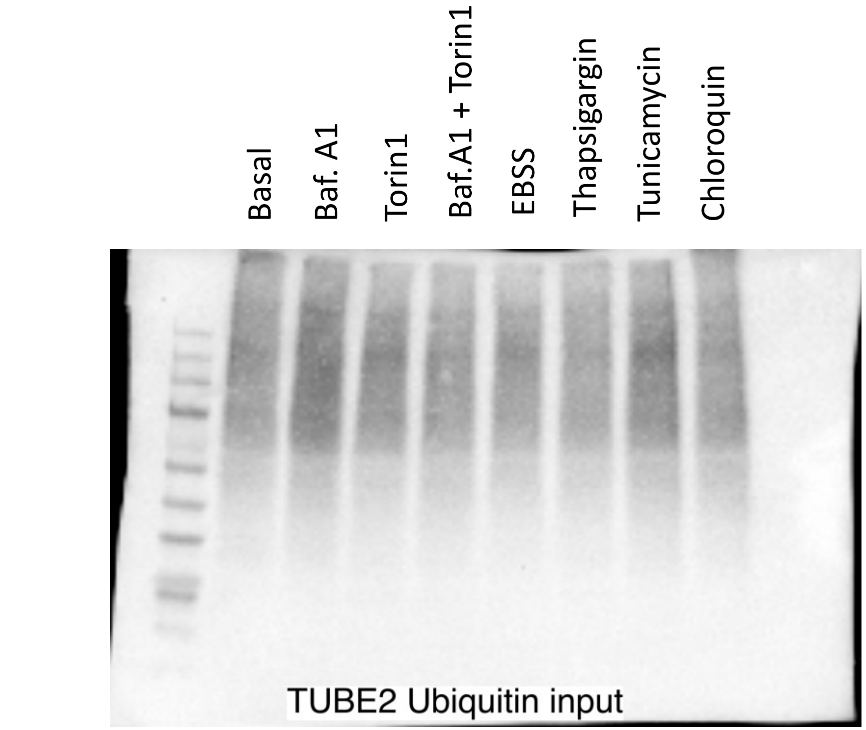
Basal
Baf. A1
Torin1
Baf.A1 + Torin1
EBSS
Thapsigargin
Tunicamycin
Chloroquin
TUBE2 Ubiquitin input

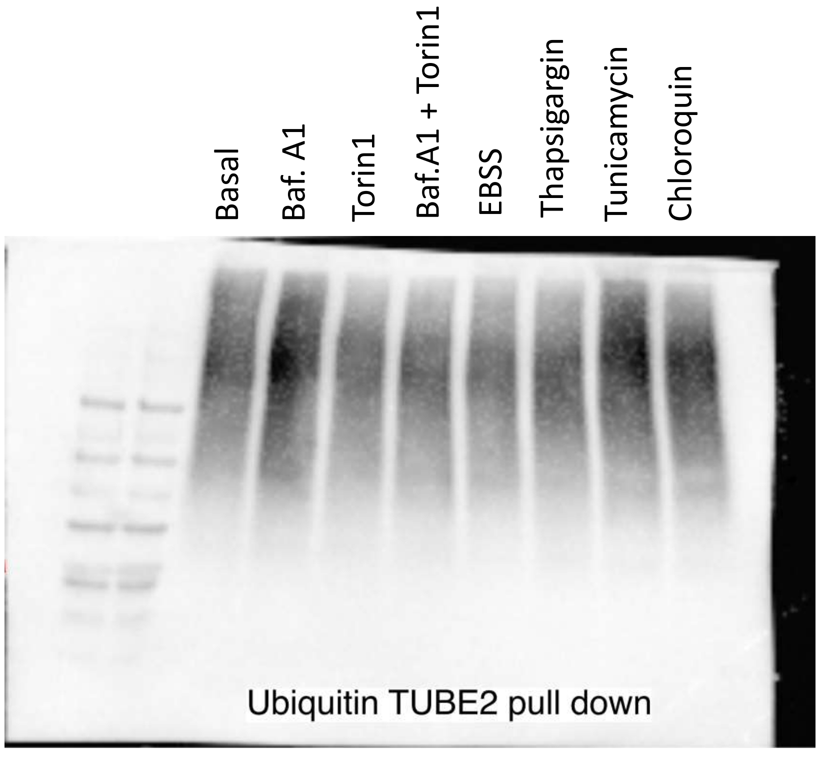
Basal
Baf. A1
Torin1
Baf.A1 + Torin1
EBSS
Thapsigargin
Tunicamycin
Chloroquin
Ubiquitin TUBE2 pull down

EXT. FIG 5. G

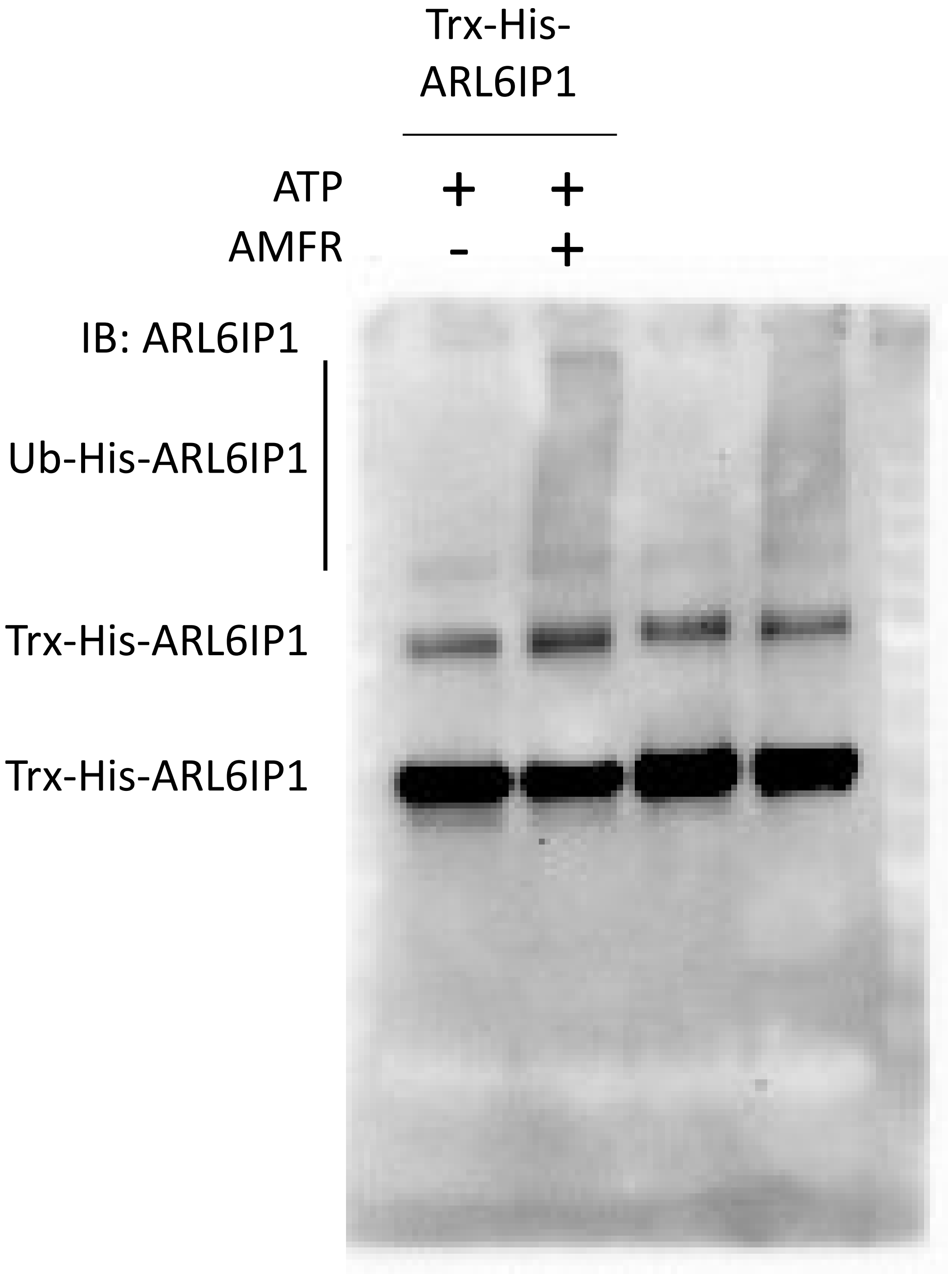

Trx-His-
ARL6IP1
ATP + +
AMFR - +
IB: ARL6IP1
Ub-His-ARL6IP1
Trx-His-ARL6IP1
Trx-His-ARL6IP1

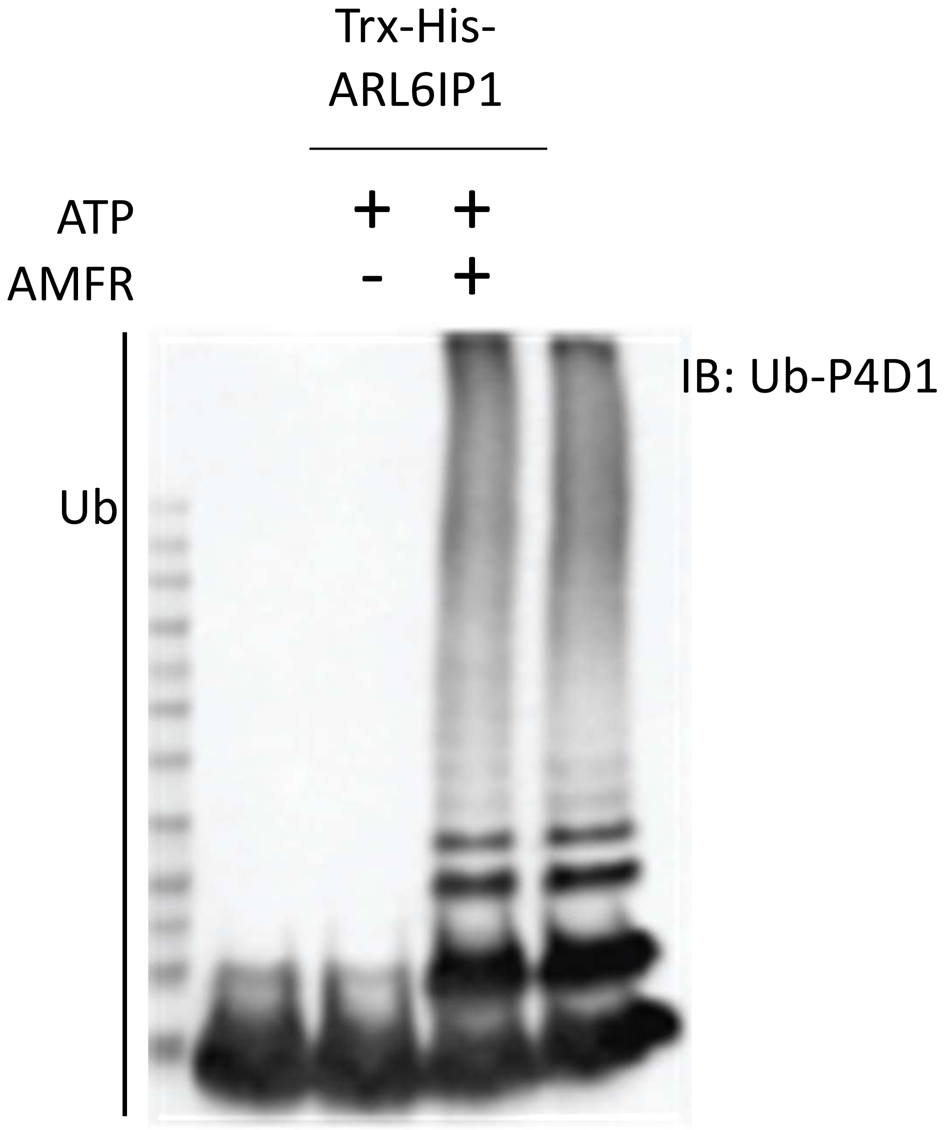

Trx-His-
ARL6IP1
ATP + +
AMFR - +
IB: Ub-P4D1
Ub

EXT. FIG 6. B

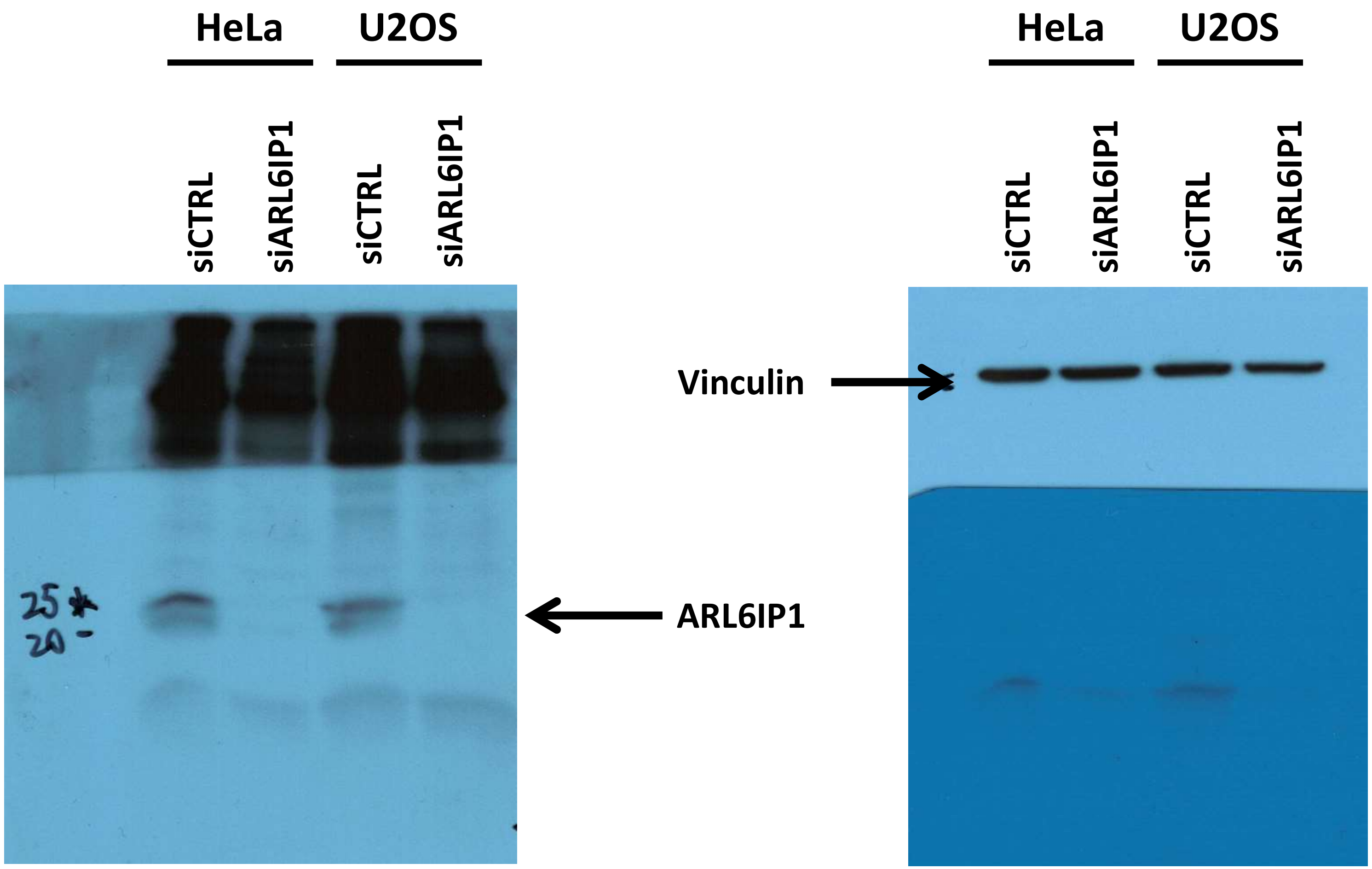

HeLa
U2OS
siCTRL
siARL6IP1
siCTRL
siARL6IP1
25
20
ARL6IP1
Vinculin
HeLa
U2OS
siCTRL
siARL6IP1
siCTRL
siARL6IP1

EXT. FIG 7. A

N1

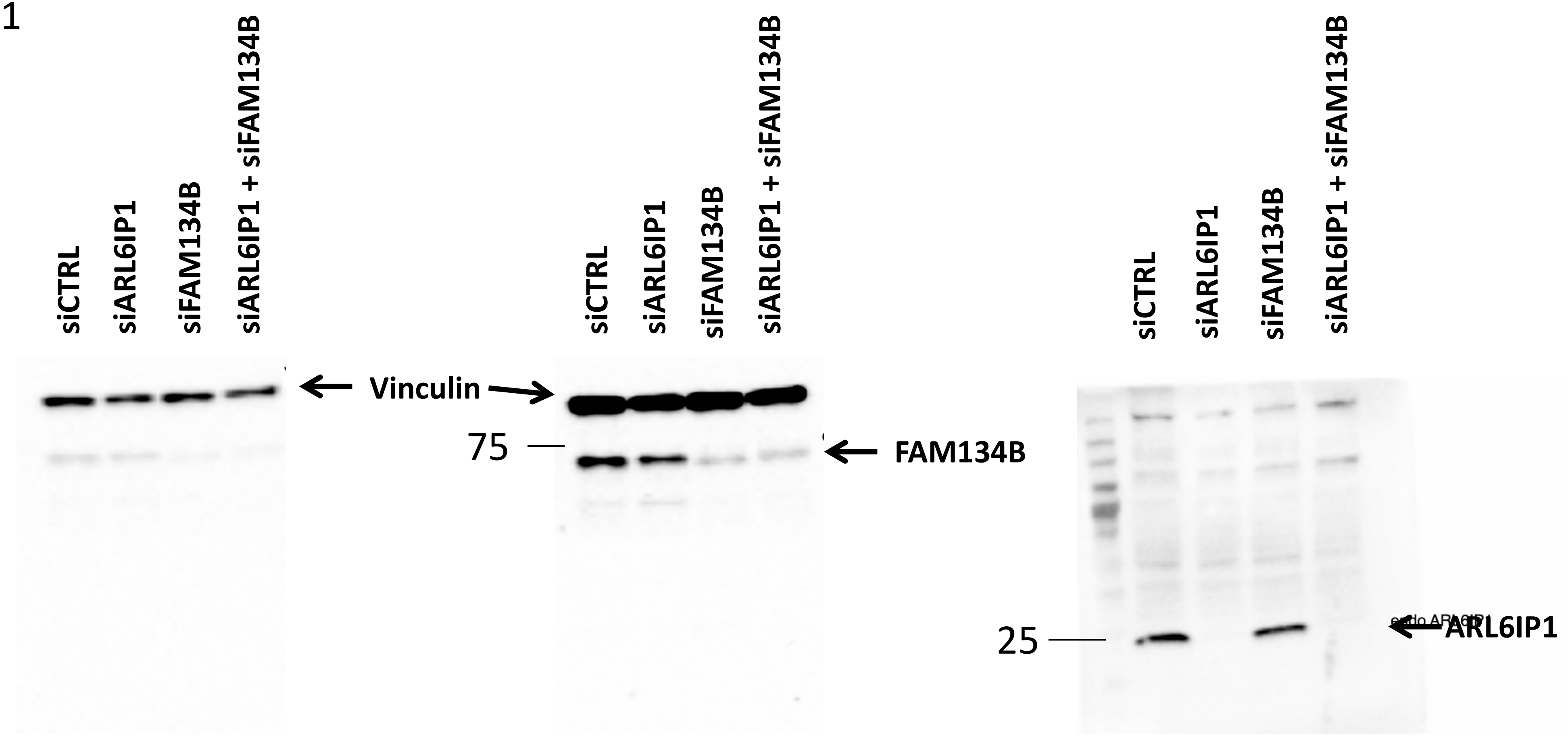

siCTRL
siARL6IP1
siFAM134B
siARL6IP1 + siFAM134B
Vinculin
75
FAM134B
siCTRL
siARL6IP1
siFAM134B
siARL6IP1 + siFAM134B
siCTRL
siARL6IP1
siFAM134B
siARL6IP1 + siFAM134B
25
ARL6IP1

EXT. FIG 7. A

N2 & N3

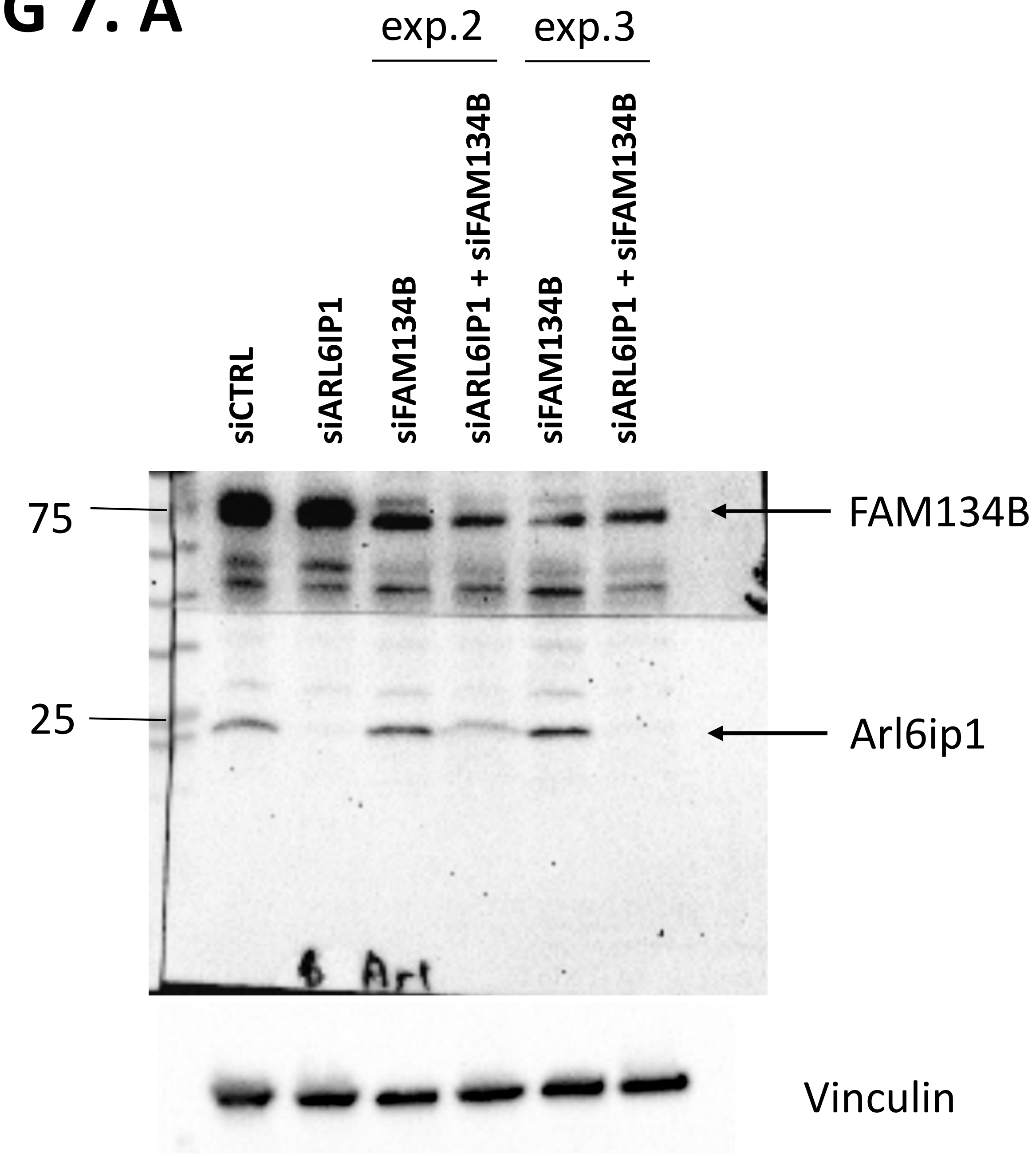

EXT. FIG 7. D

U2OS
mCherry-GFP-LC3

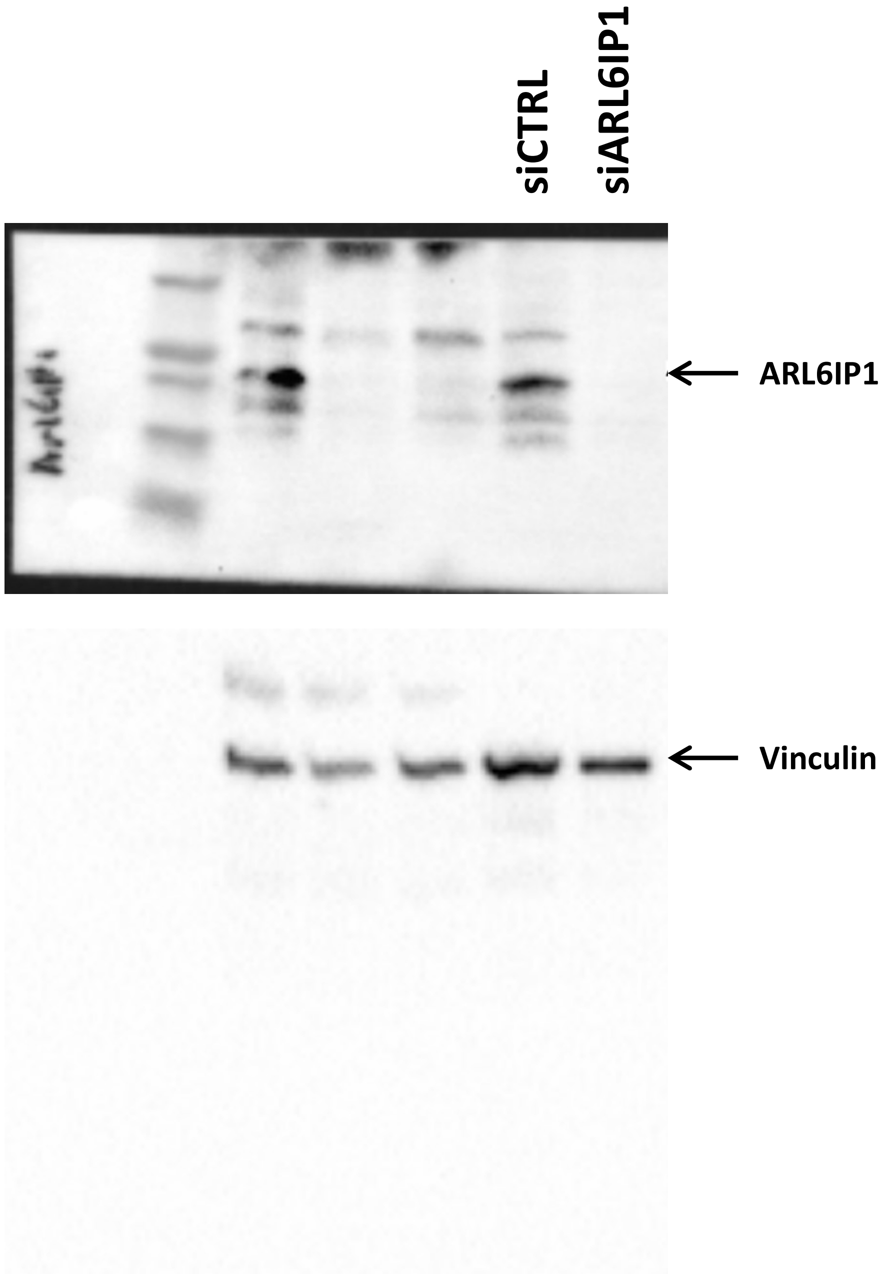

EXT. FIG 9. E

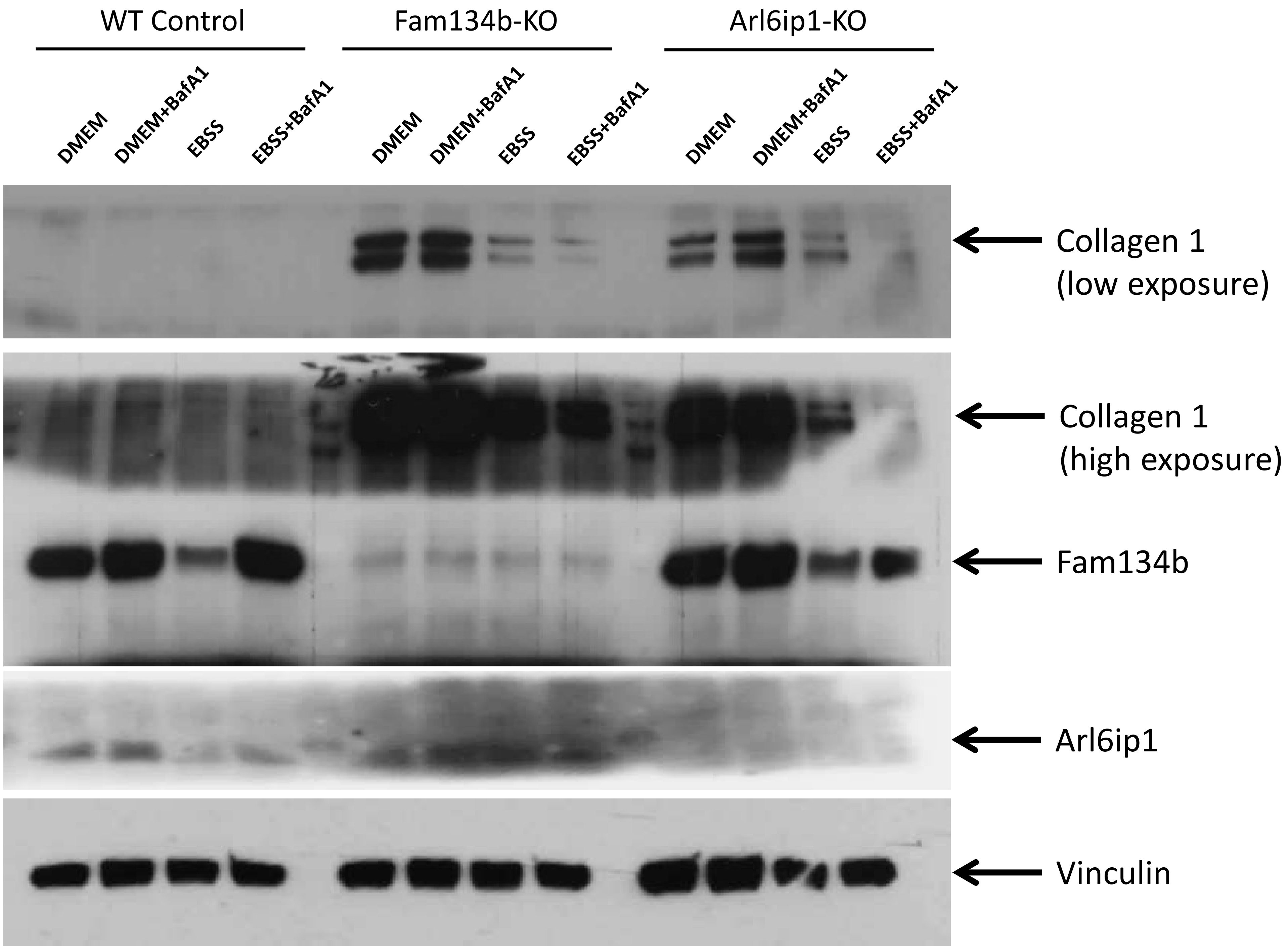
WT Control
Fam134b-KO
Arl6ip1-KO
DMEM
DMEM+BafA1
EBSS
EBSS+BafA1
DMEM
DMEM+BafA1
EBSS
EBSS+BafA1
DMEM
DMEM+BafA1
EBSS
EBSS+BafA1
Collagen 1 (low exposure)
Collagen 1 (high exposure)
Fam134b
Arl6ip1
Vinculin

Experiment 1

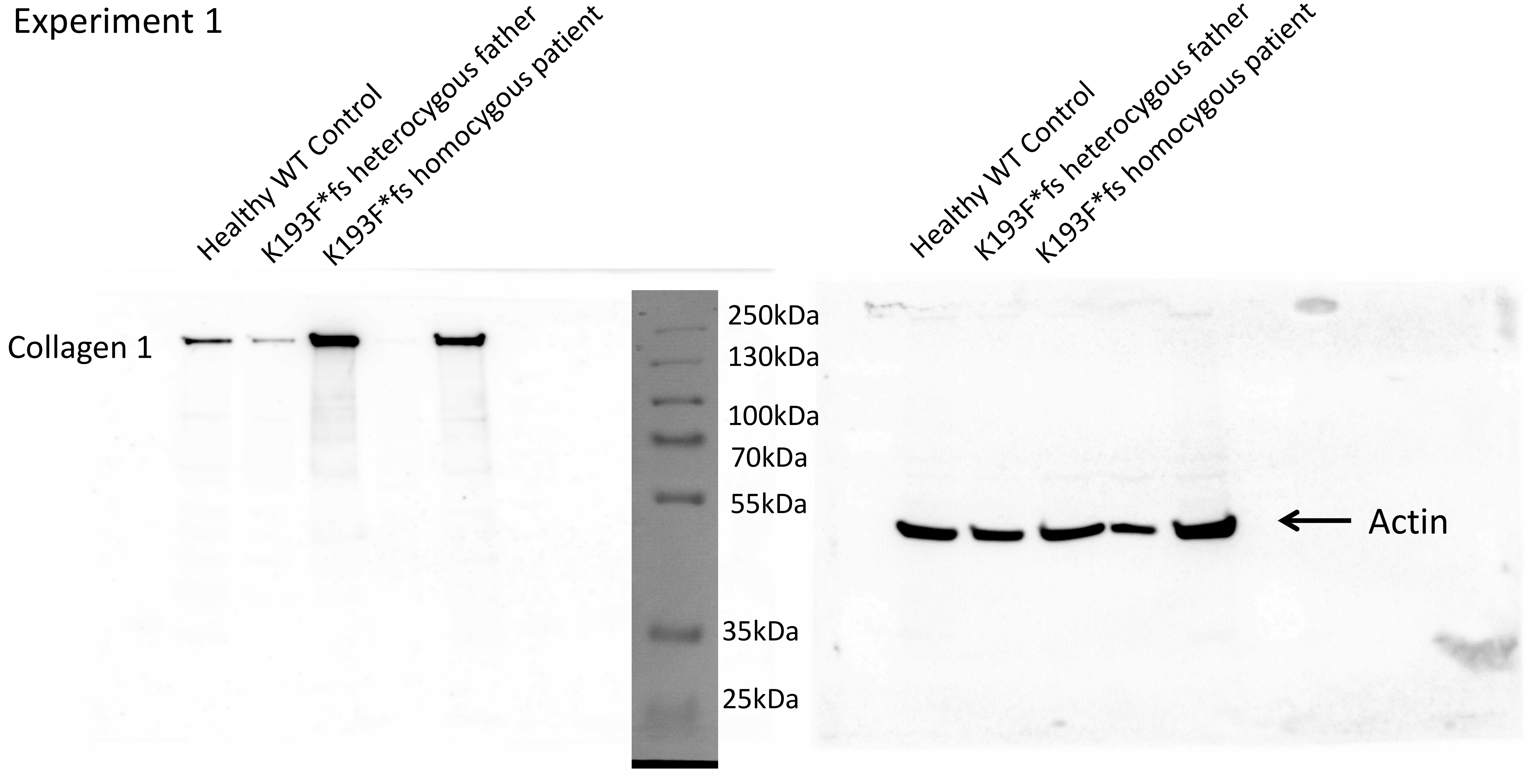

EXT. FIG 9. F

Experiment 2

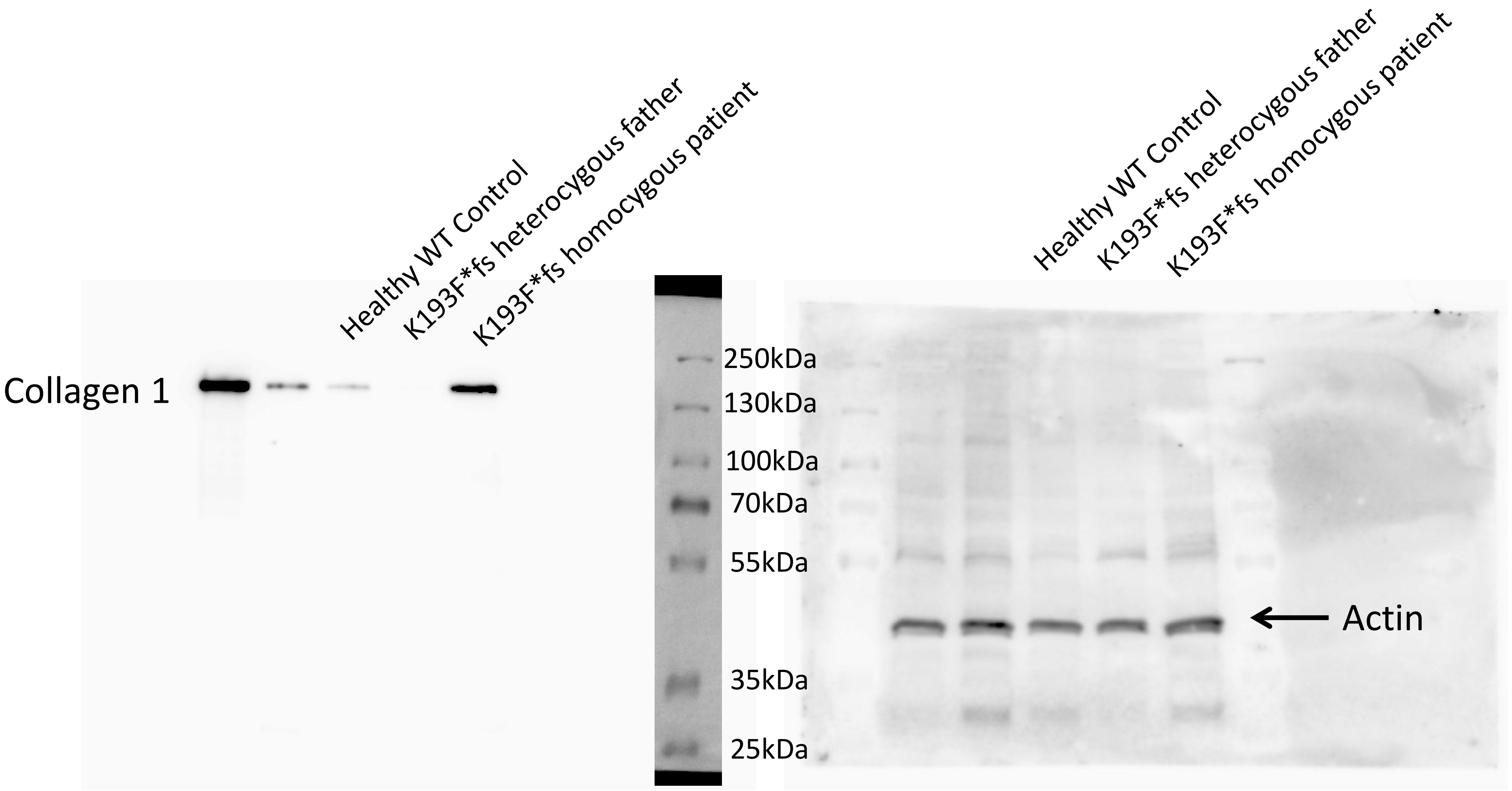

EXT. FIG 9. F

Experiment 3

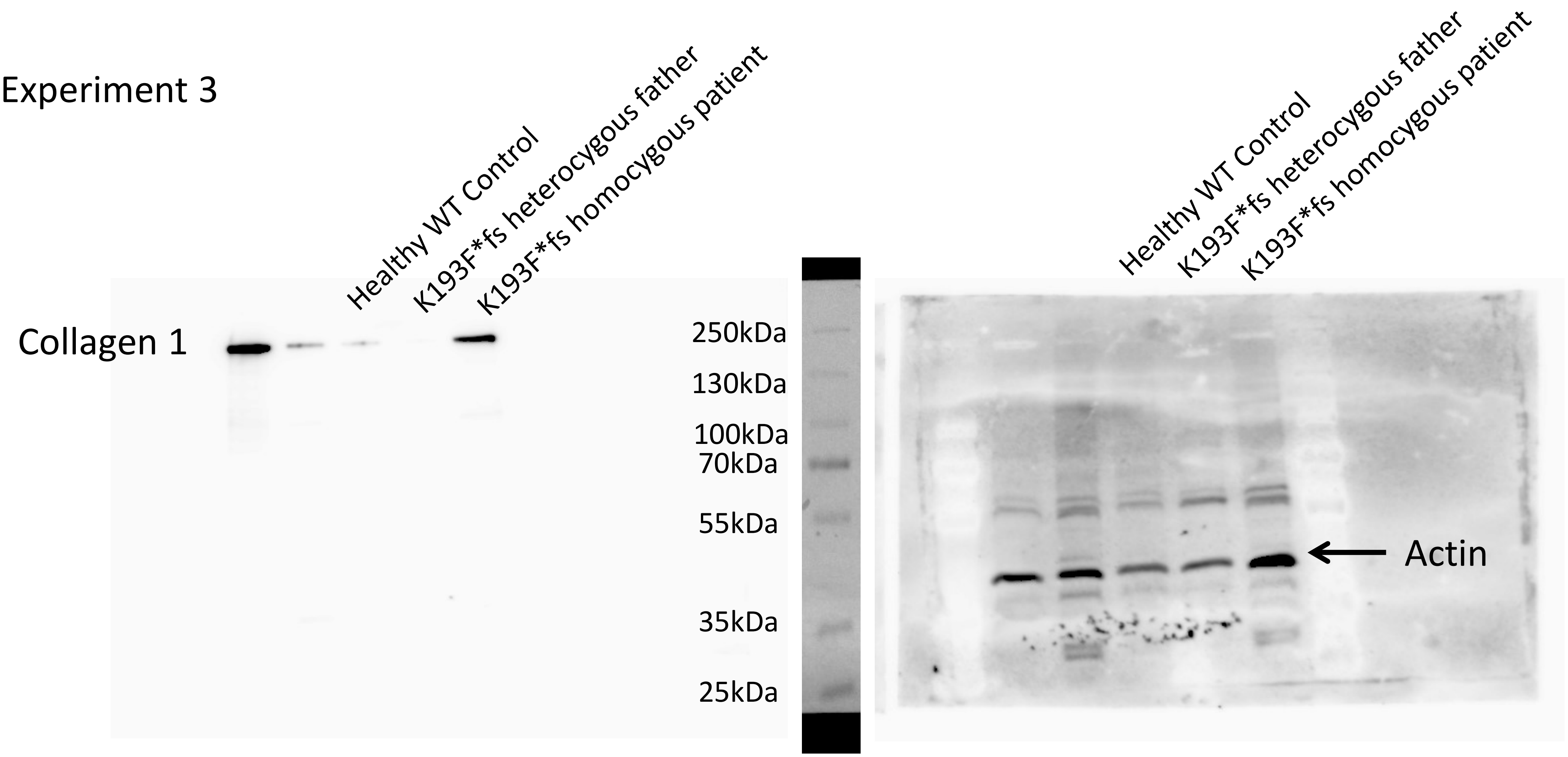

EXT. FIG 9. F
