# Supplementary material for: Heteromeric clusters of ubiquitinated ER-shaping proteins drive ER-phagy
Source: Nature. 2023 May 24;618(7964):402–10. doi: 10.1038/s41586-023-06090-9 (PMC10247384; doi:10.1038/s41586-023-06090-9)
Supplement: Supplementary file 2 — Reporting Summary [file 41586_2023_6090_MOESM2_ESM.pdf]

nature portfolio

Corresponding author(s): Christian A. Hübner and Ivan Dikic

Last updated by author(s): 30th of March 2023

Reporting Summary

Nature Portfolio wishes to improve the reproducibility of the work that we publish. This form provides structure for consistency and transparency in reporting. For further information on Nature Portfolio policies, see our Editorial Policies and the Editorial Policy Checklist.

Statistics

For all statistical analyses, confirm that the following items are present in the figure legend, table legend, main text, or Methods section.

n/a	Confirmed	
☐	☒	The exact sample size (n) for each experimental group/condition, given as a discrete number and unit of measurement
☒	☐	A statement on whether measurements were taken from distinct samples or whether the same sample was measured repeatedly
☐	☒	The statistical test(s) used AND whether they are one- or two-sided<br>*Only common tests should be described solely by name; describe more complex techniques in the Methods section.*
☒	☐	A description of all covariates tested
☒	☐	A description of any assumptions or corrections, such as tests of normality and adjustment for multiple comparisons
☐	☒	A full description of the statistical parameters including central tendency (e.g. means) or other basic estimates (e.g. regression coefficient) AND variation (e.g. standard deviation) or associated estimates of uncertainty (e.g. confidence intervals)
☐	☒	For null hypothesis testing, the test statistic (e.g. F, t, r) with confidence intervals, effect sizes, degrees of freedom and P value noted<br>*Give P values as exact values whenever suitable.*
☒	☐	For Bayesian analysis, information on the choice of priors and Markov chain Monte Carlo settings
☒	☐	For hierarchical and complex designs, identification of the appropriate level for tests and full reporting of outcomes
☐	☒	Estimates of effect sizes (e.g. Cohen's d, Pearson's r), indicating how they were calculated

Our web collection on statistics for biologists contains articles on many of the points above.

Software and code

Policy information about availability of computer code

Data collection	1. Fluorescence images were collected with the ZEN 2.3 (blue edition) (Zeiss) or with Leica Application Suite X software (version 2.0.2.15022, Leica SP8 confocal microscope).<br>2. ER-phagy flux assays were acquired with CQ1 software (version 1.04.07.01, high content microscope-Yokogawa CQ1 confocal imaging cytometer).<br>3. Western Blots signal detection was carried out with the LAS ImageQuant LAS 4000 automated detection system (GE Healthcare) or with the Image Lab software (version 6.0.1, ChemiDoc MP imaging system, Bio-Rad).<br>4. MS raw data was processed with MaxQuant (version 1.6.10.43).<br>5. We performed coarse-grained MD simulations using the MARTINI model (version 2.2)<br>6. TEM data were acquired with the ImageSP (SYSPROG).<br>11. Freeze-fractured liposomes were examined by systematic grid exploration using a transmission electron microscopy (TEM), EM900 electron microscope (Zeiss) at 80kV. Images were acquired using a wide-angle dual speed 2K CCD camera (Tröndle). Diameters of liposomes were determined using ImageJ software.
Data analysis	1. Densitometric quantification of western blot bands was carried out using ImageJ (Fiji version 1.53t) or Image Lab (version 6.0.1, Bio-Rad) for Mac.<br>2. Quantitative image analysis was carried out using the ComDet v.0.5.5 plugin ImageJ (https://github.com/ekatrukha/ComDet), cell counter v. 3.0.0 plugin (https://imagej.net/plugins/cell-counter) for Fiji v. 2.0.0-rc-68/1.52h (https://imagej.net/software/fiji/#publication), or manually.<br>3. Colocalization analysis was carried out using Coloc_2 v. 3.0.5 plugin for Fiji v. 2.0.0-rc-68/1.52h (https://imagej.net/software/fiji/#publication).<br>4. ER-phagy flux analysis was carried out using the HDD analysis software built in the CQ1 Yokogawa microscope (version 1.04.07.01).<br>5. MS raw data was processed with MaxQuant (version 1.6.10.43). Protein quantification and data normalization relied on the MaxLFQ algorithm implemented in MaxQuant (version 1.6.10.43)<br>6. For protein assignment, spectra were correlated with the Uniprot human database (version 2019) including a list of common contaminants.

7. The Perseus software (version 2.0.7.0) was used and first filtered for contaminants and reverse entries as well as proteins that were only identified by a modified peptide.
8. The data analysis and graphs were generated with GraphPad Prism 8.2.1 and 9.4.1
9. Diameters of liposomes were determined using ImageJ (version 1.53t).
10. The predicted structural model of ARL6IP1 was obtained with AlphaFold (https://alphafold.ebi.ac.uk)
11. Helical wheel representation was obtained with Heliquest (https://heliquest.ipmc.cnrs.fr)
12. The alignment of FAM134B and ARL6IP1 was carried out with the BioPython implementation of BLAST (https://biopython.org)
13. Modelling and simulations were performed using Pymol v2.54 (https://pymol.org/2) and
gromacs (v.2019.3) (https://www.gromacs.or

For manuscripts utilizing custom algorithms or software that are central to the research but not yet described in published literature, software must be made available to editors and reviewers. We strongly encourage code deposition in a community repository (e.g. GitHub). See the Nature Portfolio guidelines for submitting code & software for further information.

Data

Policy information about availability of data

All manuscripts must include a data availability statement. This statement should provide the following information, where applicable:

- Accession codes, unique identifiers, or web links for publicly available datasets
- A description of any restrictions on data availability
- For clinical datasets or third party data, please ensure that the statement adheres to our policy

The mass spectrometry proteomics data have been deposited to the ProteomeXchange Consortium via the PRIDE partner repository with the dataset identifiers pxd032718, pxd032720 and pxd039184. All source data in main and extended data figures are provided as supplementary information. This also includes gels and blots. Materials and associated protocols are available upon request without undue qualifications.

Field-specific reporting

Please select the one below that is the best fit for your research. If you are not sure, read the appropriate sections before making your selection.

☒ Life sciences ☐ Behavioural & social sciences ☐ Ecological, evolutionary & environmental sciences

For a reference copy of the document with all sections, see nature.com/documents/nr-reporting-summary-flat.pdf

Life sciences study design

All studies must disclose on these points even when the disclosure is negative.

Sample size

A sample size calculation was not done. Sample size was determined based on similar studies in this field:
e.g. Khaminets, A. et al. Regulation of endoplasmic reticulum turnover by selective autophagy. Nature 522, 354-358, doi:10.1038/nature14498 (2015); Beetz, C. et al. A spastic paraplegia mouse model reveals REEP1-dependent ER shaping. J Clin Invest 123, 4273-4282, doi:10.1172/JCI65665 (2013)

Data exclusions

No data were excluded from analysis.

Replication

To ensure reproducibility all data presented in this manuscript was repeated three times as far as possible or confirmed by different experimental approaches. E.g. ubiquitination of FAM134B was validated in different cell lines by mass spectrometry and by biochemical approaches. Single cell analysis included at least three replicates and representative images are presented (confocal and TEM images). Results from all technical-and biological replicates were consistent.

Randomization

Mass spectrometry samples were grouped as specified in the manuscript. Every data set was analyzed together (between group same experiment) to determine ubiquitination status.
Littermates of the correct genotype were randomly assigned to the respective experimental cohorts.
Cells for image analysis were selected randomly.

Blinding

The experimenter or the analyzing person was always blinded to the genotypes.

Behavioural & social sciences study design

All studies must disclose on these points even when the disclosure is negative.

Study description

Briefly describe the study type including whether data are quantitative, qualitative, or mixed-methods (e.g. qualitative cross-sectional, quantitative experimental, mixed-methods case study).

Research sample

State the research sample (e.g. Harvard university undergraduates, villagers in rural India) and provide relevant demographic information (e.g. age, sex) and indicate whether the sample is representative. Provide a rationale for the study sample chosen. For studies involving existing datasets, please describe the dataset and source.

Sampling strategy	*Describe the sampling procedure (e.g. random, snowball, stratified, convenience). Describe the statistical methods that were used to predetermine sample size OR if no sample-size calculation was performed, describe how sample sizes were chosen and provide a rationale for why these sample sizes are sufficient. For qualitative data, please indicate whether data saturation was considered, and what criteria were used to decide that no further sampling was needed.*
Data collection	*Provide details about the data collection procedure, including the instruments or devices used to record the data (e.g. pen and paper, computer, eye tracker, video or audio equipment) whether anyone was present besides the participant(s) and the researcher, and whether the researcher was blind to experimental condition and/or the study hypothesis during data collection.*
Timing	*Indicate the start and stop dates of data collection. If there is a gap between collection periods, state the dates for each sample cohort.*
Data exclusions	*If no data were excluded from the analyses, state so OR if data were excluded, provide the exact number of exclusions and the rationale behind them, indicating whether exclusion criteria were pre-established.*
Non-participation	*State how many participants dropped out/declined participation and the reason(s) given OR provide response rate OR state that no participants dropped out/declined participation.*
Randomization	*If participants were not allocated into experimental groups, state so OR describe how participants were allocated to groups, and if allocation was not random, describe how covariates were controlled.*

Ecological, evolutionary & environmental sciences study design

All studies must disclose on these points even when the disclosure is negative.

Study description	*Briefly describe the study. For quantitative data include treatment factors and interactions, design structure (e.g. factorial, nested, hierarchical), nature and number of experimental units and replicates.*
Research sample	*Describe the research sample (e.g. a group of tagged Passer domesticus, all Stenocereus thurberi within Organ Pipe Cactus National Monument), and provide a rationale for the sample choice. When relevant, describe the organism taxa, source, sex, age range and any manipulations. State what population the sample is meant to represent when applicable. For studies involving existing datasets, describe the data and its source.*
Sampling strategy	*Note the sampling procedure. Describe the statistical methods that were used to predetermine sample size OR if no sample-size calculation was performed, describe how sample sizes were chosen and provide a rationale for why these sample sizes are sufficient.*
Data collection	*Describe the data collection procedure, including who recorded the data and how.*
Timing and spatial scale	*Indicate the start and stop dates of data collection, noting the frequency and periodicity of sampling and providing a rationale for these choices. If there is a gap between collection periods, state the dates for each sample cohort. Specify the spatial scale from which the data are taken*
Data exclusions	*If no data were excluded from the analyses, state so OR if data were excluded, describe the exclusions and the rationale behind them, indicating whether exclusion criteria were pre-established.*
Reproducibility	*Describe the measures taken to verify the reproducibility of experimental findings. For each experiment, note whether any attempts to repeat the experiment failed OR state that all attempts to repeat the experiment were successful.*
Randomization	*Describe how samples/organisms/participants were allocated into groups. If allocation was not random, describe how covariates were controlled. If this is not relevant to your study, explain why.*
Blinding	*Describe the extent of blinding used during data acquisition and analysis. If blinding was not possible, describe why OR explain why blinding was not relevant to your study.*

Did the study involve field work? ☐ Yes ☒ No

Reporting for specific materials, systems and methods

We require information from authors about some types of materials, experimental systems and methods used in many studies. Here, indicate whether each material, system or method listed is relevant to your study. If you are not sure if a list item applies to your research, read the appropriate section before selecting a response.

Materials & experimental systems

n/a	Involved in the study	
☐	☒	Antibodies
☐	☒	Eukaryotic cell lines
☒	☐	Palaeontology and archaeology
☐	☒	Animals and other organisms
☒	☐	Human research participants
☒	☐	Clinical data
☒	☐	Dual use research of concern

Methods

n/a	Involved in the study	
☒	☐	ChIP-seq
☒	☐	Flow cytometry
☒	☐	MRI-based neuroimaging

Antibodies

Antibodies used

Primary:
Actin Sigma (A-5441, Lot 064M4789V)
AMFR Proteintech (16675-AP, Lot: 00046373)
ARL6IP1 Sigma (PRS3305, Lot 33050404)
ARL6IP1 Atlas Antibodies (HPA045307, Lot B118670)
ATL2 Proteintech (16688-1-AP, Lot 00053330
ATL3 Proteintech (16921-1-AP, Lot 00008332)
CCPG1 polyclonal rabbit, affinity purified with N-term peptide, gift from Simon Wilkinson
CLIMP63 (CKAP4) Proteintech (16686-1-AP, Lot 00045668)
CLIMP63 (CKAP4) R&D Systems (AF7355, Lot CGDG0118071)
FAM134B Proteintech (21537-I-AP, Lot 00014408)
FAM134B Genscript. Please, request ID and LOT number to Dikic laboratory.
FLAG (M2) Sigma (F3165, Lot SLBQ7119V) - monoclonal
FLAG Sigma (F7425, Lot 0000131574)
GABARAP Abcam (ab109364, Lot:GR3232141-2)
GAPDH Cell signaling (2118, Lot:14)
GFP Clontech (632460, Lot 2007065)
GFP Roche (11814460001)
GFP Santa Cruz (sc-9996, Lot K1616)
GFP Proteintech (3H9, Lot 60706001AB) - monoclonal
GST Santa Cruz (sc-138, Lot K1814)
HA-Tag Roche (11867423001, Lot: 60789700)
RGS.His Qiagen (34650) - monoclonal
dsRED Clontech (632496)
Collagen I Abcam (ab138492, Lot GR247379-65)
Collagen I Abcam (ab21286, Lot GR3273324-1)
Collagen I DSHB (SP1.D8, Lot 2ea 11/1/18) - monoclonal
LAMP1 Abcam (Ab24170, Lot GR3235361-1)
LAMP1 DSHB (1D4B, Lot 2ea 5/19/11) - monoclonal
LAMP1 DSHB (H4A3, Lot 4ea 2/12/15) - monoclonal
LAMP2 DSHB (ABL-93-c, Lot 1ea 1/23/20) - monoclonal
LC3B Cell Signaling (2775S, Lot 10)
LC3B MBL (M152-3, Lot: 057) - monoclonal
LC3B 5F10 Nano tools (0231-100, Lot: 0260S0603) - monoclonal
LC3B MBL (PM036, Lot: 035)
Mono-polyubiquitin FK2 Biomol (BML-PW8810, Lot 08072015)
Myc-Tag (9B11) Cell Signaling (2276S, Lot 24) - monoclonal
Myc-Tag Sigma (M5546, Lot 0000090421) - monoclonal
NeuN Millipore (MAB377, Lot 3519281) - monoclonal
REEP1 Proteintech (17988-1-AP, Lot 00017226)
REEP2 Proteintech (15684-1-AP, Lot 00053153)
REEP5 Proteintech (14643-1-AP, Lot: 00042892)
REEP5 Santa Cruz BT (sc-393508, Lot K0317)
RTN1 Abcam (ab9274, Lot GR3451813-1)
RTN2 Proteintech (11168-1-AP, Lot 00014465)
RTN3 Proteintech (12055-2-AP, Lot 00045087)
RTN4 Abcam (ab47085, Lot GR259948-1)
Sec62 Novusbio (NBP1-84045, Lot B118889)
Ubiquitin-P4D1 Cell Signalling (3936, Lot 19)
Vinculin Sigma (V4505/V913, Lot 000013524)

Secondary:
HRP-conjugated anti-rat Cell Signaling (#7077S, Lot 13)
HRP-conjugated anti-rabbit GE Healthcare (NA9340)
HRP-conjugated anti-rabbit Dako (P0448) Lot: 20053537
HRP-conjugated anti-mouse GE Healthcare (NA9310)
HRP-conjugated anti-mouse IgG BioRad (#1706516, Lot 64482134)
HRP-conjugated anti-rat Abcam (ab97057)

Anti-guinea pig IRDye680 LICOR Bioscience (925-32411)
Anti-guinea pig IRDye800 LICOR Bioscience (926-32411)
Anti-rabbit Alexa 405 Invitrogen (A31556, Lot 799246)
Anti-rabbit Alexa 488 Life Technology (A21206, Lot 2256732)
Anti-rabbit Alexa 532 Invitrogen (A11008, Lot 1719682)
Anti-rabbit Alexa 488 Invitrogen (A11008, Lot 2284595)
Anti-rabbit Alexa 647 Life Technology (A21244, Lot 1696456)
Anti-rabbit Alexa 680 Thermo Fischer (A-21109)
Anti-rabbit DyLight800 Thermo Fischer (A-35571)
Anti-rabbit Cy5 Invitrogen (A10523, Lot 2286294)
Anti-mouse Alexa 488 Life Technology (A21202, Lot 2428531)
Anti-mouse Alexa 546 Invitrogen (A11003, Lot 2155294)
Anti-mouse Alexa 647 Invitrogen (A31571, Lot 2136787)
Anti-mouse Cy3 MerckMillipore (#AP124C, Lot 3067473)
Anti-rat Alexa 488 Life Technology (A21208, Lot 2092264)
Anti-rat Cy3 MerckMillipore (#AP189C, Lot 3028089)
Anti-rat Cy5 Invitrogen (A10525, Lot 1902490)
Anti-sheep Alexa 555 Invitrogen (A21436, Lot 54811A)

Validation

All commercial antibodies were used as indicated by the supplying company and for the recommended species. Antibodies against FAM134B and ARL6IP1 were validated with KO tissues or by Western blot. If available, the specificity of primary antibodies was further controlled by co-stainings with alternative markers. In Western blots we also verified the appropriate size of the targeted protein. The specificity of secondary antibodies in immunostainings were controlled by immunostainings with omission of primary antibodies.

Eukaryotic cell lines

Policy information about cell lines

Cell line source(s)

HEK293T, U2OS and HeLa cells were obtained from ATCC. U2OS TRex cells were provided by Prof. Stephen Blacklow (Brigham and Women's Hospital and Harvard Medical School), which are also based on stocks provided by ATCC. Human cells (father and patient) were obtained from Joe Gleeson (University of California). Mouse embronic fibroblasts lines were established in the lab of CAH.

Authentication

Cell line authentication was initially performed by ATCC. Cell lines were further confirmed by genotyping and by microscopy, as all cell lines used in this study (HEK293T, HeLa or U2OS Trex) have quite distinct morphologies.

Mycoplasma contamination

We tested for contamination every month. No contamination was found.

Commonly misidentified lines
(See ICLAC register)

No commonly misidentified cell lines were used in this study.

Animals and other organisms

Policy information about studies involving animals; ARRIVE guidelines recommended for reporting animal research

Laboratory animals

ARL6IP1 KO mice were generated in house with clone HEPD0752_7_D11 (EUCOMM).
All studies were performed in mice, which had been backcrossed for at least 4 generations.
All animal experiments with animals were done with mixed sexes with balanced male and female numbers in WT and KO cohorts.
In detail: Fig. 1f and g: WT 3 males and 3 females, KO 2 males 3 females; Figure 1i and l: WT 3 males and 3 females, KO 3 males 3 females. Ext. Data Figure 1a: WT 5 males and 4 females, KO 5 males and 6 females; Ext. Data Figure 1b: WT 1 male and 2 females, KO 1 male and 2 females; Ext. Data Figure 1c: 2m and 22m WT 1 male and 2 females, KO 1 male and 2 females; Ext. Data Figure 1e: WT 1 male and 2 females, KO 1 males and 2 females; Ext. Data Figure 1f and h: WT 3 females, KO 3 females; Ext. Data Figure 11: WT 2 males and 3 females, KO 2 males and 3 females.
The age at analysis is indicated in the legends and varied between 2 and 22 months of age.
15- to 30-week-old F1 female offspring from C57BL/6J and CBA/J matings served as foster mice.
Conditions of maintenance: 21°C±2°C, air humidity min. 45%, 15 fold air exchange,14h/10h day/night cycle, max. 500lx. Standard mouse chow and water ad libitum.

Wild animals

No wild animals were used in this study.

Field-collected samples

No field collected samples were used in the study.

Ethics oversight

All animal experiments were performed within existing licenses supplied by the „Thüringer Landesamt für Lebensmittelsicherheit und Verbraucherschutz (TLLV)" registration numbers 02-055/14 and UKJ-17-006.

Note that full information on the approval of the study protocol must also be provided in the manuscript.
