# Supplementary material for: Heteromeric clusters of ubiquitinated ER-shaping proteins drive ER-phagy
Source: Nature. 2023 May 24;618(7964):402–10. doi: 10.1038/s41586-023-06090-9 (PMC10247384; doi:10.1038/s41586-023-06090-9)
Supplement: Supplementary file 3 — Plasmids used in the study. [file 41586_2023_6090_MOESM3_ESM.docx]

**Supplementary Table 1. Plasmids used in the study.**

| **Plasmid** | **Mutation / Tagging** | **Usage** | **Source** |
| --- | --- | --- | --- |
| pClneo-ARL6IP1-Myc | C-Myc | Transient transfection | This study |
| pClneo-ARL6IP1-FLAG | C-FLAG | Transient transfection | This study |
| pClneo-ARL6IP1 K193Ffs-FLAG | Deletion of 576-579 AAAC/C-FLAG | Transient transfection | This study |
| pCIneo-ATL3 |  | Transient transfection | This study |
| pEGFP-N2-FAM134A |  | Transient transfection | This study |
| pEGFP-N2-FAM134B |  | Transient transfection | This study |
| pEGFP-N2-FAM134C |  | Transient transfection | This study |
| CD3-RFP | YFP replaced by RFP | Transient transfection | Modified  from^1^ |
| RFP-KDEL | pcDNA3.1 | Transient transfection | Addgene #138660 |
| pCDNA3.1-ARL6IP1-7KR-HA | Substitution of K35, K96, K114, K124, K130, K188 and K193 by R | Transient transfection | This study |
| pET32a-Trx-His-ARL6IP1 | N terminal Trx-His | Transformation of *E. coli* | This study |
| pET32a-Trx-His-ARL6IP1-7KR | N terminal Trx-His | Transformation of *E. coli* | This study |
| pcDNA3.1-FLAG-ARL6IP1 | N-FLAG | Transient transfection | This study |
| pDONR233-ARL6IP1 (without C-STOP codon) |  | GATEWAY entry vector for pDEST plasmids | This study |
| pDONR233-ARL6IP1  (with C-STOP codon) |  | GATEWAY entry vector for pDEST plasmids | This study |
| pDONR233-ARL6IP1- ΔAHC | Deletion of C-terminal amphipathic helix (ΔLys182-Lys200) | GATEWAY entry vector for pDEST plasmids. | This study |
| pDONR233-ARL6IP1- ΔAHL | Deletion of amphipathic helix within the central loop (ΔGlu100-Lys130) | GATEWAY entry vector for pDEST plasmids. | This study |
| pDONR233-ARL6IP1- Δ(AHL+AHC) | Deletion of both amphipathic helices (ΔGlu100-Lys130 and ΔLys182-Lys200) | GATEWAY entry vector for pDEST plasmids. | This study |
| pDONR233-ARL6IP1- Δ(TM1+2) | Deletion of transmembrane domains 1+2 (ΔAla42-Leu83) | GATEWAY entry vector for pDEST plasmids. | This study |
| pDONR233-ARL6IP1- Δ(TM3+4) | Deletion of transmembrane domains 3+4 (ΔMet136-Gly178) | GATEWAY entry vector for pDEST plasmids. | This study |
| pDONR233-ARL6IP1- Δ(TM1-4) | Deletion of the transmembrane domains 1-4, loop preserved (ΔAla42-Leu83 and ΔMet136-Gly178) | GATEWAY entry vector for pDEST plasmids. | This study |
| pDONR233-ARL6IP1- ΔAHC+KKNE | Deletion of C-terminal amphipathic helices, KKNE preserved (ΔLys182-Glu199) | GATEWAY entry vector for pDEST plasmids. | This study |
| pDONR233-ARL6IP1- Δ(AHL+AHC)+KKNE | Deletion of both amphipathic helices, KKNE preserved (ΔGlu100-Lys130 and ΔLys182- Glu199) | GATEWAY entry vector for pDEST plasmids. | This study |
| pcDNA3.1-SBP-FLAG-ARL6IP1 | N-SBP-FLAG | GATEWAY enter vector | This study |
| pcDNA3.1-HA-ARL6IP1 | N-HA | GATEWAY enter vector | This study |
| pcDNA3.1-HA-ARL6IP1-7KR | N-HA | GATEWAY enter vector | This study |
| pcDNA3.1-HA-ARL6IP1- ΔAHC | N-HA. Deletion of C-terminal amphipathic helix (ΔLys182-Lys200) | GATEWAY enter vector | This study |
| pcDNA3.1-HA-ARL6IP1- ΔAHL | N-HA. Deletion of amphipathic helix within the central loop (ΔGlu100-Lys130) | GATEWAY enter vector | This study |
| pcDNA3.1-HA-ARL6IP1- Δ(AHL+AHC) | N-HA. Deletion of both amphipathic helices (ΔGlu100-Lys130 and ΔLys182-Lys200) | GATEWAY enter vector | This study |
| pcDNA3.1-HA-ARL6IP1-Δ(TM1+2) | N-HA. Deletion of transmembrane domains 1+2 (ΔAla42-Leu83) | GATEWAY enter vector | This study |
| pcDNA3.1-HA-ARL6IP1- Δ(TM3+4) | N-HA. Deletion of transmembrane domains 3+4 (ΔMet136-Gly178) | GATEWAY enter vector | This study |
| pcDNA3.1-HA-ARL6IP1- Δ(TM1-4) | N-HA. Deletion of transmembrane domains 1-4, loop preserved (ΔAla42-Leu83 and ΔMet136-Gly178) | GATEWAY enter vector | This study |
| pcDNA3.1-HA-ARL6IP1- ΔAHC+KKNE | N-HA. Deletion of C-terminal amphipathic helices, KKNE preserved (ΔLys182-Glu199) | GATEWAY enter vector | This study |
| pcDNA3.1-HA-ARL6IP1- Δ(AHL+AHC)+KKNE | N-HA. Deletion of both amphipathic helices, KKNE preserved (ΔGlu100-Lys130 and ΔLys182- Glu199) | GATEWAY enter vector | This study |
| pcDNA5FRT/TO-N-mCherry-EGFP-FAM134B | N-mCherry-GFP | Transient transfection | This study |
| pcDNA3.1-HA-FAM134B | N-HA | GATEWAY enter vector | ^2^ |
| pcDNA3.1-HA-FAM134B ΔAHC | N-HA, deletion of C-terminal amphipathic helix | GATEWAY enter vector | This study |
| pcDNA3.1-HA-FAM134B ΔAHL | N-HA, deletion of amphipathic helix within the central loop | GATEWAY enter vector | This study |
| pcDNA3.1-HA-FAM134B Δ(AHC+L) | N-HA, deletion of both amphipathic helices | GATEWAY enter vector | This study |
| pcDNA3.1-HA-FAM134B △(TM1+2) | Deletion of transmembrane domains 1+2 | Transient transfection | ^3^ |
| pcDNA3.1-HA-FAM134B △(TM3+4) | Deletion of transmembrane domains 3+4 | Transient transfection | ^3^ |
| pcDNA3.1-HA-FAM134B △(TM1-4) | Deletion of transmembrane domains 1-4 and loops | Transient transfection | ^3^ |
| pcDNA3.1 AMFR-FLAG |  | Transient transfection | Addgene #62370 |
| pcDNA3.1 AMFR RING mut-FLAG | Mutated catalytic RING domain: C356G H361A | Transient transfection | Addgene #61751 |
| pHAGE-GFP-FAM134B | N-GFP | Transient transfection | This study |
| V1-FAM134B | N-terminal V1, which is the nonfluorescent N-terminal part of Venus (Met1-Gln157). | Transient transfection | This study |
| V2-FAM134B | N-terminal V2, which is the nonfluorescent C-terminal part Venus (Lys158-Lys238) | Transient transfection | This study |
| V1-ARL6IP1 | N-terminal V1 | Transient transfection | This study |
| V2-ARL6IP1 | N-terminal V2 | Transient transfection | This study |
| V1-ARL6IP1-7KR | N-terminal V1 | Transient transfection | This study |
| V2-ARL6IP1-7KR | N-terminal V2 | Transient transfection | This study |
| V2-ARL6IP1-Δ(TM1+2) | N-terminal V2 | Transient transfection | This study |
| AMFR-V1 | C-terminal V1 | Transient transfection | This study |
| AMFR-V2 | C-terminal V2 | Transient transfection | This study |
| AMFR-V1-C356G H361A | C-terminal V1 | Transient transfection | This study |
| AMFR-V2-C356G H361A | C-terminal V2 | Transient transfection | This study |
| V1-CCPG WT | N-terminal V1 | Transient transfection | This study |
| mCherry-GFP-FIS1 | Reporter for mitophagy | Transient transfection | ^4^ |
| pcDNA3.1-mCherry-EGFP-LC3 | N-mCherry-EGFP | Constantly expressing cell line generation | ^2^ |
| pPal7-ARL6IP1 | N terminal Profinity eXact affinity tag | Transformation of *E. coli* | This study |
| pGEX6.GST-ARL6IP1 | N-terminal GST-Tag | Bacterial expression for Protein purification | This study |
| pGEX6.GST-ARL6IP1-7KR | N-terminal GST-Tag | Bacterial expression for Protein purification | This study |
| pLentiCRISPR v2-sgRNA1 | Lentiviral plasmid contains sgRNA1 of ARL6IP1 | Generation of ARL6IP1 CRISPR-CAS9 KO cell line | This study |
| pLentiCRISPR v2-sgRNA2 | Lentiviral plasmid contains sgRNA2 of ARL6IP1 | Generation of ARL6IP1 CRISPR-CAS9 KO cell line | This study |
| pLentiCRISPR v2-sgRNA3 | Lentiviral plasmid contains sgRNA3 of ARL6IP1 | Generation of ARL6IP1 CRISPR-CAS9 KO cell line | This study |
