# Supplementary material for: Heteromeric clusters of ubiquitinated ER-shaping proteins drive ER-phagy
Source: Nature. 2023 May 24;618(7964):402–10. doi: 10.1038/s41586-023-06090-9 (PMC10247384; doi:10.1038/s41586-023-06090-9)
Supplement: Supplementary file 4 — Primers used for this study. [file 41586_2023_6090_MOESM4_ESM.docx]

**Table 2. Primers used for this study.**

| **Primer name** | **Primer sequences** |
| --- | --- |
| ARL6IP1-WT GATEWAY forward | 5’-GGGGACAAGTTTGTACAAAAAAGCAGGCTGCatggcggagggagataatcgc -3’ |
| ARL6IP1-WT GATEWAY reverse (with C-term STOP codon) | 5’-GGGGACCACTTTGTACAAGAAAGCTGGGTCTCAttcgtttttcttttctttttgtttgag -3’ |
| ARL6IP1-WT GATEWAY reverse (without C-term STOP codon) | 5’-GGGGACCACTTTGTACAAGAAAGCTGGGTCttcgtttttcttttctttttgtttgag -3’ |
| ARL6IP1-WT, aa1-7 forward  (BamH1) | 5’- CAGGATCCatggcggagggagataatcg -3’ |
| ARL6IP1-WT reverse aa200-203 (with C-term STOP codon) (EcoRI) | 5’- CCACGCGTGaattccctattcgtttttc -3’ |
