# Supplementary material for: Heteromeric clusters of ubiquitinated ER-shaping proteins drive ER-phagy
Source: Nature. 2023 May 24;618(7964):402–10. doi: 10.1038/s41586-023-06090-9 (PMC10247384; doi:10.1038/s41586-023-06090-9)
Supplement: Supplementary file 5 — Primary antibodies and their application related to this study. [file 41586_2023_6090_MOESM5_ESM.docx]

**Supplementary Table 3. Primary antibodies and their application related to this study**

| **Antigen** | **Company and ID** | **Application and dilution** |
| --- | --- | --- |
| ARL6IP1 | Sigma (PRS3305) | WB (1/1000) |
| ARL6IP1 | Atlas Antibodies (HPA045307) | WB (1/500) |
| Actin | Sigma (A-5441) | WB (1/1000) |
| ATL2 | Proteintech (16688-1-AP) | WB (1/500) |
| ATL3 | Proteintech (16921-1-AP) | WB (1/500) |
| AMFR | Proteintech (16675-AP) | WB (1/1000) IF (1/300) |
| LC3B 5F10 | Nano tools (0231-100) | WB (1/1000) |
| LC3B | MBL (M152-3) | IF (1/500) |
| LC3B | MBL (PM036) | IF (1/500) |
| REEP5 | Proteintech (14643-1-AP) | WB, IF (1/1000) |
| REEP5 | Santa Cruz BT (sc-393508) | IF (1/200) |
| CCPG1 | polyclonal rabbit, affinity purified with N-term peptide, gift from Simon Wilkinson | IF (1/200) |
| CLIMP63 (CKAP4) | Proteintech (16686-1-AP) | WB (1/1000) |
| CLIMP63 (CKAP4) | R&D Systems (AF7355) | IF (1:250) |
| FLAG (M2) | Sigma (F3165-5MG) | WB (1/5000), IF (1/1000) |
| FLAG | Sigma (F7425-2MG) | WB (1/500), IF (1/100) |
| FAM134B | Genscript | WB (1/1000) |
| FAM134B | Proteintech (21537-I-AP) | WB (1/2000) |
| GABARAP | Abcam (ab109364) | WB (1/1000) |
| GAPDH | Cell signaling (2118) | WB (1/5000) |
| GFP | Clontech (Cat. 632460) | WB (1/1000) |
| GFP | Roche (11814460001) | WB (1/2000) |
| GFP | Santa Cruz (sc-9996) | IF (1/200) |
| GFP | Proteintech (3H9) | IF (1/500) |
| GST | Santa Cruz (sc-138) | WB (1/1000) |
| HA-Tag | Roche (11867423001) | IF (1/250), WB (1/1000) |
| RGS.His | Qiagen (34650) | WB (1/1000) |
| dsRED | Clontech (632496) | WB (1/1000), IF (1/500) |
| Collagen I | Abcam (ab138492) | WB (1/1000), IF (1/300) |
| Collagen I | Abcam (ab21286) | WB (1/500), IF (1/300) |
| Collagen I | DSHB (SP1.D8) | WB (1/1000), IF (1/50) |
| LAMP1 | Abcam (Ab24170) | WB (1/1000), IF (1/500) |
| LAMP2 | DSHB (ABL-93-c) | IF (1/500) |
| LC3B | Cell Signaling | IF (1/250), WB (1/1000) |
| LC3B | Nano tools (0231-100) | WB (1/1000) |
| LC3B | MBL (M152-3) | IF (1/500) |
| LAMP1 | DSHB (H4A3) | IF (1/500) |
| LAMP1 | DSHB (1D4B) | IF (1/500) |
| Myc-Tag (9B11) | Cell Signaling (2276S) | WB (1/2000), IF (1/4000) |
| Myc-Tag | Sigma (M5546) | IF (1/250) WB (1/1000) |
| NeuN | Millipore (MAB377) | IF (1/500) |
| REEP1 | Proteintech (17988-1-AP) | WB (1/1000) |
| REEP2 | Proteintech (15684-1-AP) | WB (1/1000) |
| RTN1 | Abcam (ab9274)) | WB (1/1000) |
| RTN2 | Proteintech (11168-1-AP) | WB (1/1000) |
| RTN3 | Proteintech (12055-2-AP) | WB (1/1000) |
| RTN4 | Abcam (ab47085) | IF (1/250), WB (1/1000) |
| Sec62 | Novusbio NBP1-84045 | IF (1:250) |
| Mono-polyubiquitin FK2 | Biomol (BML-PW8810) | IF (1/500) |
| Ubiquitin-P4D1 | Cell Signalling (3936) | WB (1/1000) |
| Vinculin | Sigma (V4505) | WB (1/5000) |
