# Supplementary material for: Heteromeric clusters of ubiquitinated ER-shaping proteins drive ER-phagy
Source: Nature. 2023 May 24;618(7964):402–10. doi: 10.1038/s41586-023-06090-9 (PMC10247384; doi:10.1038/s41586-023-06090-9)
Supplement: Supplementary file 6 — Secondary antibodies and their application related to this study. [file 41586_2023_6090_MOESM6_ESM.docx]

**Supplementary Table 4. Secondary antibodies and their application related to this study**

| **Antigen** | **Company and ID** | **Application and dilution** |
| --- | --- | --- |
| HRP-conjugated anti-rat | Cell Signaling (#7077S) | WB (1/4000) |
| HRP-conjugated anti-rabbit | GE Healthcare (NA9340) | WB (1/4000) |
| HRP-conjugated anti-mouse | GE Healthcare (NA9310) | WB (1/4000) |
| HRP-conjugated anti-rat | Abcam (ab97057) | WB (1/4000) |
| Anti-guinea pig IRDye680 | LICOR Bioscience (925-32411) | WB (1/10000) |
| Anti-guinea pig IRDye800 | LICOR Bioscience (926-32411) | WB (1/10000) |
| Anti-rabbit Alexa 408 | Invitrogen (A31556) | IF (1/500) |
| Anti-rabbit Alexa 488 | Life Technology (A21206) | IF (1/500) |
| Anti-rabbit Alexa 488 | Invitrogen (A11008) | IF (1/1000) |
| Anti-rabbit Alexa 647 | Life Technology (A21244) | IF (1/500) |
| Anti-rabbit Alexa 680 | Thermo Fischer (A-21109) | WB (1/10000) |
| Anti-rabbit DyLight800 | Thermo Fischer (A-35571) | WB (1/8000) |
| Anti-rabbit Cy5 | Invitrogen (A10523) | IF (1/1000) |
| Anti-mouse Alexa 488 | Life Technology (A21202) | IF (1/500) |
| Anti-mouse Alexa 546 | Invitrogen (A11030) | IF (1/1000) |
| Anti-mouse Alexa 647 | Invitrogen (A31571) | IF (1/500) |
| Anti-mouse Cy3 | MerckMillipore (#AP124C) | IF (1/500) |
| Anti-rat Alexa 488 | Life Technology (A21208) | IF (1/500) |
| Anti-rat Cy3 | MerckMillipore (#AP189C) | IF (1/500) |
| Anti-rat Cy5 | Invitrogen (A10525) | IF (1/1000) |
| Anti-sheep Alexa 555 | Invitrogen (A21436) | IF (1/1000) |
