# Supplementary material for: Heteromeric clusters of ubiquitinated ER-shaping proteins drive ER-phagy
Source: Nature. 2023 May 24;618(7964):402–10. doi: 10.1038/s41586-023-06090-9 (PMC10247384; doi:10.1038/s41586-023-06090-9)
Supplement: Supplementary file 7 — sgRNA sequences used to generate the ARL6IP1 KO cell lines. [file 41586_2023_6090_MOESM7_ESM.docx]

**Supplementary Table 5: sgRNA sequences used to generate the ARL6IP1 KO cell lines**

| Gene | sgRNA sequence | Guide Nr. |
| --- | --- | --- |
| ARL6IP1 | 5’- CACCGGGCTGATAAAGTCCTCCGAT -3’ | FW_1 |
|  | 5’- AAACATCGGAGGACTTTATCAGCCC -3’ | RW_1 |
|  | 5’- AAACGCGTTTCCTGTTTTGTTATGC -3’ | FW_2 |
|  | 5’- CACCGCATAACAAAACAGGAAACGC -3’ | RW_2 |
|  | 5’- AAACGCTGGCTGCAGAGACTGCAAC -3’ | FW_3 |
|  | 5’- CACCGTTGCAGTCTCTGCAGCCAGC -3’ | RW_3 |
